# Diversity of Phylogenetic Information According to the Locus and the Taxonomic Level: An Example from a Parasitic Mesostigmatid Mite Genus

**DOI:** 10.3390/ijms11041704

**Published:** 2010-04-13

**Authors:** Lise Roy, Ashley P.G. Dowling, Claude Marie Chauve, Thierry Buronfosse

**Affiliations:** 1 Laboratoire de Parasitologie, Université de Lyon, Ecole Nationale Vétérinaire de Lyon, Marcy-L’Etoile, France; E-Mails: c.chauve@vet-lyon.fr (C.M.C.); thierry.buronfosse@inserm.fr (T.B.); 2 Department of Entomology, University of Arkansas, Fayetteville, USA; E-Mail: adowling@uark.edu

**Keywords:** phylogenetic signal, evolution of specialization, *Dermanyssus*, Acari, Mesostigmata

## Abstract

Molecular markers for cladistic analyses may perform differently according to the taxonomic group considered and the historical level under investigation. Here we evaluate the phylogenetic potential of five different markers for resolving evolutionary relationships within the ectoparasitic genus *Dermanyssus* at the species level, and their ability to address questions about the evolution of specialization. COI provided 9–18% divergence between species (up to 9% within species), 16S rRNA 10–16% (up to 4% within species), ITS1 and 2 2–9% (up to 1% within species) and Tropomyosin intron n 8–20% (up to 6% within species). EF-1α revealed different non-orthologous copies within individuals of *Dermanyssus* and *Ornithonyssus.* Tropomyosin intron n was shown containing consistent phylogenetic signal at the specific level within *Dermanyssus* and represents a promising marker for future prospects in phylogenetics of Acari. Phylogenetic analyses revealed that the generalist condition is apomorphic and *D. gallinae* might represent a complex of hybridized lineages. The split into *hirsutus*-group and *gallinae*-group in *Dermanyssus* does not seem to be appropriate based upon these results and *D. longipes* appears to be composed of two different entities.

## 1. Introduction

The question of the evolution of ecological specialization is a fundamental one that may be partly addressed through phylogenetic analyses. Habitat or resource range may be dictated partly by extrinsic ecological parameters (biotic and/or abiotic environmental factors) and partly by intrinsic characteristics. The specialist/generalist condition may be imposed by ecological factors such as competition and mating success, or simply may be historically acquired. As a result, phylogenetic reconstructions are expected to help assess the evolution of specialization in a given group.

It has been often presumed that specialists evolve from generalists [1], but several recent studies involving host specificity in host-parasite systems [2], food resources in bees [3], or habitat in springtails [4] show generalists in distal positions. In these examples, specialization appears to be plesiomorphic and does not appear to be a “dead end”. Futuyma and Moreno [1] pointed out that the irreversibility of specialization is more likely to occur when phenotypes have been highly modified (reduced, lost characters). In such cases, specialization is likely to strongly restrict evolutionary flexibility. On the other hand, generalization may have a cost [5] also restricting evolutionary plasticity.

It has been commonly assumed that specialized species, because of their narrow tolerance and consequently fragmented distribution, are more prone to allopatric speciation than are generalists [1]. As a result, reduced gene flow between specialized lineages might contribute to fixation of characters which otherwise would have been lost in the framework of extensive outbreeding (which generates an increased effective population size). But Kaci-Chaouch *et al.* [6] showed that generalist species within the genus *Lamellodiscus*, which are more derived diplectanid monogeneans [2], have more genetic and morphological diversity than specialists. This contradicts the hypothesis of a positive relationship between specialization and rate of diversification.

The mite genus *Dermanyssus* is involved in a rather loose host-parasite system and therefore represents an interesting model for testing the trends in the evolution of specialization. Following Kuris and Lafferty [7], *Dermanyssus* species in the *gallinae*-group *sensu* Moss [8] are considered micropredators rather than typical parasites because adult females feed successively on different host individuals like mosquitoes or bed bugs. Blood meals are taken rapidly resulting in engorgement [9] and are limited in number (one per nymphal stage and one before each oviposition in adult females). Members of the *gallinae*-group are nidicolous in nature, laying eggs and spending much time off the host. In contrast, species in the *hirsutus*-group are more typical ectoparasites, living and developing on the host [8].

Despite the loose host relationship in the *gallinae-*group, they exhibit various levels of host specificity. Some species that had previously been considered to have a very broad host range turned out to be more specific than thought, likely due to previous misidentifications [10]. Roy *et al.* [10,11] highlighted an opposition between two main clades, which seemed to be correlated with the level of host specificity. On one hand, there was the *D. gallinae* clade with generalist species (nine different bird orders), including diverse lineages, of which some may by potentially cryptic species. On the second hand there was a clade of four specialist species (*D. hirundinis*, Passeriform hosts, a single bird family in France, *D. longipes*, Passeriform hosts, 2 bird families, *D. carpathicus*, id., and *D. apodis*, Apodiform host, a single bird genus) [10]. Moreover, *D. gallinae* is the only synanthropic species (encountered in bird farms). Although the studies of Roy *et al.* [10,11] reached a solid delineation between species, they did not succeed in resolving relationships between specific entities. This was due to the use of mt DNA data only [10] or to the weakly informative variations in the selected nDNA fragment (ITS1-5.8S-ITS2) [11].

The present study has the following two objectives:

1. Assess the utility of two additional nuclear markers - elongation factor 1-α (EF-1α), Tropomyosin exon n, intron n and exon n + 1(Tpm) - for the exploration of relationships between and within species belonging to *Dermanyssus* and comparing these results to previous studies [10, 11]
2. Address the following questions about the evolution of host specificity in *Dermanyssus*:
  - Are the lineages of the generalist *D. gallinae’s* lineages effectively composed of cryptic species (potentially making them as specialized as any of the four specialist species)?
  - Is the generalist condition derived or ancestral?

For such purposes, we performed phylogenetic analyses based on a combination of different gene fragments. Analyses aim to not only disentangle relationships between species but also to further explore species delineations (*i.e.*, check the reproductive status of some mitochondrial lineages previously noticed within delineated entities). Therefore, the degree of coalescence of some intraspecific clades was compared between mt and nDNA topologies and a comparison of observed intraspecific variability was carried out.

## 2. Material and Methods

Usually, in order to improve phylogenetic analyses, increasing the number of characters or the number of taxa may be helpful. In the present study, we adopt a two-step strategy: first, a multi-gene analysis was performed on a reduced set of isolates, based on five different genes representing six different datasets (**Step 1**). Then a multi-isolate analysis was performed using one mitochondrial and one nuclear marker developed in the first stage (**Step 2**), since less isolated lineages were detected in previous DNA studies [10,11]. This second approach focused on intraspecific and intra-isolate variability, by using sequences of two independent and highly variable loci. Although not expected to fill in taxonomic gaps, this was expected to provide finer resolution of relationships within previously delimited specific entities in order to enable detection of cryptic isolated lineages [11]. The focus is on the five following species: *D. gallinae*, *D. apodis*, *D. carpathicus*, *D. hirundinis*, *D. longipes*.

### 2.1. Biological Material

The location, host species, mite species, accession numbers of mites under test are listed in Appendix 1. Mite isolates have been sampled from wild bird nests or from farms as described in Roy *et al.* [10]. The distribution of samples is rather large and diverse within France, and includes a few samples from other countries (ex. The Netherlands, Poland, USA)(Appendix 1). Nests were analyzed using a method described by De Lillo [12] involving immersion of the nest followed by filtering, except that no sodium hypochlorite was added to the water solution to wash the stack of sieves and that the sieves had a somewhat different mesh width (top to bottom: 2,500 μm, 1,400 μm, 180 μm, 100 μm).

One isolate corresponds to mites of a single *Dermanyssus* species, isolated from an individual nest or from a group of nests closely located to each other in a bird colony (wild avifauna) or from a single building (farms). From each isolate, 1–5 individuals have been separately sequenced. Additionally, for five of these isolates (1 *D. apodis*: GO, 1 *D. carpathicus*: BER7, 2 *D. gallinae s. str.*: SK, IL, 1 *D.gallinae* special lineage L1: 9001; see Roy *et al.* 2009a), 18–24 individuals have been separately sequenced, in order to get an overview of the intrapopulation variation. Moreover, 21 individuals belonging to *D. hirundinis* collected from barn swallows distributed around France were included in the analyses and are referred to as DhirF. These six groups are used specifically for statistical analyses.

### 2.2. DNA Data

Data are composed of DNA sequences obtained following the method described in Roy *et al.* [10,11] and newly obtained DNA sequences following the procedures below. The previously tested DNA regions include partial 18S–28S rRNA, including complete ITS1, ITS2 and 5.8 S (ITS), partial 16S rRNA and partial coding gene for cytochrome oxidase I (COI). The newly developed markers are one intron flanked by two portions of the coding gene for Tropomyosin and a portion of the coding gene for elongation factor 1-alpha (EF-1α).

Sequences of Tropomyosin and EF-1α were obtained as in Roy *et al.* [11], with PCR involving annealing temperatures of 56 °C and 57 °C, respectively. Primer sequences and pairs are provided in Appendix z2.

*D. gallinae* has been shown to be haplodiploid with diploid females developing from fertilized eggs [13,14]. As these authors also observed similar haplodiploidy in a closely related family (Macronyssidae), we assume here that other *Dermanyssus* species reproduce the same way. As a result, only adult females were integrated in the study for standardization, notably to regularly detect allelic variation and to avoid potentially confounding effects of the male haploid genome.

In case of heterozygosity in the Tropomyosin targeted fragment, the two alleles were separated from each other using internal primers (cf. Appendix z2), or in some cases by cloning. As for EF-1α some sequences were cloned to separate double copies found after PCR. Vector cloning was necessary for separation of multi-copies sequences in some cases. PCR products were gel purified (Qiagen, Les Ulis France) and corresponding fragments were cloned into TOPO^®^ TA cloning vector according to manufacturers’ protocols. Transformant clones were checked by restriction enzyme profile and five positive clones were submitted to sequencing.

### 2.3. Datasets

DNA and amino acid alignments were performed using MUSCLE 3.7. Without refinement, MUSCLE has been shown to achieve accuracy statistically indistinguishable from T-Coffee and MAFFT, but overall is the fastest of the tested methods for large numbers of sequences [15]. Seaview 4.0 [16] was used for DNA and amino-acid alignment handling. Six different matrices, composed of DNA sequence alignments, were generated during **Step 1** and three different matrices, two of which are DNA sequence alignments and one is a digital matrix, during **Step 2**.

## Step 1: multi-gene analyses

Six matrices are composed of assumed independent data as defined by Li and Lecointre [17]. The matrix combining the six datasets as labeled in Table 1 is available in Treebase (ID number 10389).

## Step 2: multi-isolate analyses

Haplotype alignments for Tropomyosin and COI include data from 257 individuals, representative of 40 isolates of *D. gallinae* (146 individuals), 6 isolates of *D. carpathicus* (40 individuals), 2 isolates of *D. apodis* (25 individuals), 10 isolates of *D.* hirundinis (22 individuals), 3 isolates of *D. longipes* (8 individuals), plus 1 individual of *D. hirsutus*, 1 of *D. quintus* and 14 of outgroup species (see Appendix 1 and Treebase).

Additionally, in order to test the phylogenetic utility of indels in Tropomyosin intron n, a matrix of indels in the Tropomyosin intron n has been elaborated by encoding as discrete characters the presence/absence and, when present, polymorphism of inserts at points where gaps are noted in the alignment of the whole Tropomyosin dataset. These indels have been encoded as if they were morphological or biochemical characters (character states and matrix are available in Appendix z3).

The haplotype alignments of COI and Tpm are available in Treebase (ID number 10389).

### 2.4. Phylogenetics

#### 2.4.1. Phylogenetic Analyses

Phylogenetic analyses with Maximum parsimony (MP) were run using PAUP* 4.0b10 under the same parameters as in Roy *et al.* [11]. In order to explore phylogenetic information from indels, MP analyses were performed using either the encoded indels matrix or sequence alignments with gaps as missing data or as a fifth state.

In addition to parsimony, Bayesian analyses were run using MrBayes incorporating the most appropriate model of evolution for each dataset as determined in MrModeltest using Akaike information criterion as in Roy *et al.* [11]. Since MrBayes allows each partition to be simultaneously analyzed under a separate model of evolution, each dataset was analyzed with its most appropriate model (COI: GTR + G + I; 16S: GTR + G; 5.8S, ITS, Tropo_int, Tropo_ext: HKY + I). In cases of single gene analyses, only the most appropriate model of evolution for each dataset was used in the respective analysis. Parameters within the model were not specified (or fixed) and MrBayes was left to estimate these independently from the data during analysis. Analyses in MrBayes included two independent runs, each consisting of four chains and 10,000,000 generations for the total combined dataset and 2,000,000 generations for each of the independent datasets. Appropriate burnins were determined based on stationarity being reached through the use of Tracer v1.4 [18].

#### 2.4.2. Outgroups

Trees were rooted using the outgroup method. Outgroup mites sequenced include *Ornithonyssus sylviarum* and *O. bacoti* (Parasitiformes: Mesostigmata: Dermanyssoidea Macronyssidae), *Androlaelaps casalis* (Parasitiformes: Mesostigmata: Dermanyssoidea: Laelapidae), and *Typhlodromus pyri* (Parasitiformes: Mesostigmata: Ascoidea: Phytoseiidae). Additionally, some sequences have been sampled from EMBL database for two other mesostigmatid mites, *Tropilaelaps* (accession numbers: ITS EF02474, COI EF025423) and *Varroa* (accession numbers: ITS EF025470, COI and 16S NC_004454).

Concerning EF-1α, due to apparent paralogy and in order to roughly estimate the divergence point between some of the obtained paralogous copies, more distant outgroups have been included (additional sequences drawn from EMBL bank, other Parasitiformes plus some Acariformes; accession numbers: EU152805, EU152810, EU152811, EU152815, EU152816, EU152823, EU152832, EU152837, EU152840, EU152844, EU152853, U90048, AY624011, AF240836, AF240849, AF240856, AF240851, AF240860, AY624008, AY624009, AAT58070). Note that outgroup method used here for tree rooting is not an ancestry method. The aim of outgroup inclusion is simply to check the target group’s monophyly and explore its internal evolutionary history.

Lastly, although variously distant outgroups have been included into analyses, no very close outgroup have been sampled. Dermanyssidae currently include two genera, *Dermanyssus* and *Liponyssoides.* Despite repeated attempts to collect species of *Liponyssoides* in its typical host mammals, we did not manage to find any individuals of this genus. As a result, in the present study, monophyly of Dermanyssidae is tested, but the monophyly of *Dermanyssus* is not.

#### 2.4.3. Clade Robustness Support Values

Two different methods for estimating clade support have been used here: classical node support values (bootstrap, BPP) in **Steps 1** and **2**, as well as a newly developed index [17] in **Step 1**. Indeed, in order to avoid the problem of stochastic effects of homoplasy in single gene analyses, the method proposed by Li and Lecointre [17] offers the opportunity to simultaneously estimate robustness of both single gene and combined analyses. This consists of observing clades obtained using all partial or total combination possibilities in partitioning schemes which each includes all the elementary datasets and noting their occurrence. A repetition index is drawn from it.

For such a purpose, phylogenetic reconstructions of elementary datasets and of all possible combinations (two to six elementary datasets) were computed using PAUP 4.0 (MP) (with 1,000 random additions instead of 10,000). Notation of clade occurrence based on each 50% majority rule consensus tree, calculation of all possible partitioning schemes and automation of the consecutive estimation of repetition indices following Li and Lecointre [17] were performed using macro functions (VBA) in Excel.

#### 2.4.4. Comparison of Mitochondrial *versus* Nuclear Monophylies

Due to some differences in the effective population size between mitochondrial and nuclear DNA evolution, the monophyly of alleles is expected to appear more quickly in mt-DNA than in nuclear DNA. In most sexually reproducing animals (diplodiploids), cytoplasmic DNA is effectively haploid and maternally inherited, as opposed to nuclear DNA, which is diploid and biparentally transmitted. Consequently, they have a genetically effective population size approximately four times smaller than that of nuclear loci [19]. In the present case, the ratio is reduced to three times due to the haplodiploidy of *D. gallinae* (see above). In order to estimate the degree of structure within the newly developed nuclear marker Tropomyosin, the ratio external/internal branch length in the Bayesian mitochondrial toplogy (**Step 2**) has been observed in delimited specific entities following Roy *et al.* [11] and in some additional intraspecific clades, and correlated with monophylies in the Tropomyosin topologies.

Additionally, in order to detect isolated lineages within species boundaries, the most supported lineages in **Step 2** (both mt and nDNA) have been examined by mapping isolates on the haplotypic topologies and by comparing the amount of shared isolates.

### 2.5. Statistical Analysis of Haplotype Frequencies and Diversity

Some statistical analyses were performed between and within the six focused isolates, representing three species. They were performed using the isolate DNA alignments of COI haplotype sequences on one hand, and individual Tropomyosin alleles (phased alleles in heterozygous individuals and duplicated homozygous sequences, in such a way that sequences represent the diploid state of chromosomes) on the other hand.

Polymorphism in haplotype sequences (COI haplotypes and separated Tropomyosin alleles) within the eight “focused isolates” was examined (gaps excluded and as the fifth state in Tropomyosin) using DnaSP v5 [20]. We estimated the number of segregating sites (S), average number of nucleotide differences (k), and haplotype diversity (Hd). Pairwise genetic distances were computed using Fst (Hudson *et al.* 1992) and statistical significance assessed after 1000 permutations in all cases using Arlequin 3.1 [21] (haplotypic dataset for COI and genotypic dataset for Tropomyosin in order to avoid potential bias due to departure to the Hardy-Weinberg equilibrium).

## 3. Results

### 3.1. DNA Sequences

#### 3.1.1. Alignments of Obtained Gene Fragments

Three DNA alignments are available in Treebase (accession number: 10389): the matrix 6comb combining the six elementary datasets and corresponding to the partitioning scheme psc 203 (Appendix 4) of **Step 1** and the COI and the Tpm haplotypic alignments of **Step 2**.

Obtained sequences of rRNA, internal transcribed spacers and intron show variable lengths: 16S rRNA and 5.8S rRNA, ITS1 and ITS2, Tropomyosin intron n. Other sequences are strictly composed of gene portions coding for proteins and did not include stop codons nor indels: COI, EF-1α, Tropomyosin exons n and n + 1.

In 16S rRNA, a portion corresponding to a stem-loop structure known to be highly variable in some ticks if compared to *Drosophila yakuba* (between positions 200 and 255 in Black and Piesman [22]) shows variability between specific entities including numerous indels. Indels being correlated to the secondary structure in this region, the unambiguity of alignment in this portion is not established. However, excision or not of this portion did not change present results.

ITS1 and 2 are amazingly stable within and between species within genera compared to some other mites (cf. Below, Section 3.2.2). As a result, they are unambiguously aligned within genera.

In Tropomyosin intron n, indels are numerous, but unambiguously aligned as already noted in two EF-1α introns by Sanchis *et al.* [23] and by Kawakita [24].

No ambiguity has been detected in protein coding nucleic sequences.

#### 3.1.2. Molecular Characteristics of Obtained Gene Fragments

Fragments of 16S rRNA, COI and ITS obtained in the present study correspond to those used by Roy *et al.* [10, 11].

The nuclear EF-1α gene fragment corresponds to positions 303–891 of the *Heliconius melpomene* (Insecta:Lepidoptera) EF-1α gene and 102–298 of the *H. melpomene* protein (complete CDS, accession number: GQ452009). All sequences were free of stop codons. Moreover, no intron has been isolated in any mesostigmatid individual under test in the present study.

**Figure 1.**
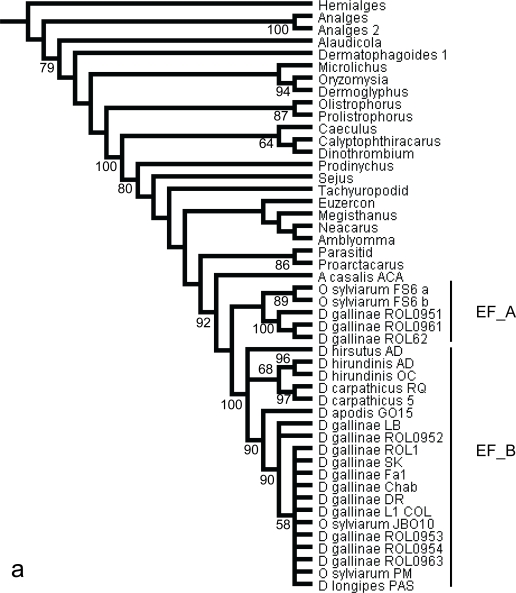
**Strict consensus of 95 equiparsimonious trees** inferred from nucleotide EF-1α sequences. Tree length = 1714, CI = 0.3215, RI = 0.6909. Numbers at nodes correspond to bootstrap values (only values > 50% are indicated). The three following groups of sequences have been isolated in a single individual: *O_sylviarum_*FS6a and b, *D_gallinae*_ROL0951, 2, 3 and 4, *D_gallinae*_ROL0961, 2, 3 and 4. Trees are rooted using *Hemialges* as an outgroup. EF_A and EF_B represent a bipartition apparently generated by gene duplication.

Note: The homology of obtained EF-1α sequences is far from certain. EF-1α copies obtained from *Ornithonyssus* and *Dermanyssus* group into two different clades containing both genera, not only when analysis is processed on nucleic sequences (clades EF-A and EF-B in Figure 1), but also on translated amino acid sequences (results not shown). Multiple copies of EF-1α belonging to the two clades EF_A and EF_B have been detected in single individuals (O. *sylviarum* FS5, FS6, *D. gallinae* ROL09) It is likely that most individuals contain similar multiple copies, although only a few of them amplified multiple copies in a single PCR run, due to primer mismatch or competition as already noted in some spiders by Hedin and Maddison [25]. Moreover, there are sequences shared between some ingroups and outgroups (O. *sylviarum* PM and JBO10, *D. gallinae* - all isolates -, *D. longipes* PAS) in clade EF_B. Consequently, this locus has been discarded from elementary datasets and the matrix 1comb6 has been reduced to Tropomyosin exon n at n +1 alone (Table 1).

The nuclear Tropomyosin gene fragment involved in the whole analysis corresponds to 10 bp of exon n, a 585–664 bp intron n and 15 bp of exon n + 1. Intron n is located between positions 551 and 552 of the coding gene in *Boophilus microplus*, based on the complete CDS published in GenBank (AF124514) and between positions 490 and 491 of the CDS sequence of *D. gallinae* published by Nisbet *et al.* [26] (AM167555). In order to check the homology of aligned introns, larger Tropomyosin fragments from 1–2 individuals of four *Dermanyssus* species were first sequenced (individuals of *D.apodis* GO593 and MAR1, *D. gallinae* 8004b, *D. carpathicus* RQ18, *D. longipes* JBO49DL2; see species and EMBL accession number in Appendix 1). This way, five sequences, including a 62–115 bp portion of exon n, the focused intron and a 53–80 bp portion of exon n + 1 were aligned.

This allowed confirmation of homology. Additionally, an alignment was performed with the above sequences after the intron was removed. The portion of coding regions provided in the present study was exactly the same as the corresponding part in the *D. gallinae* CDS sequence in extended sequences of individuals of *D. gallinae*, *D. carpathicus* and *D. longipes*. In the sequences of the two individuals of *D. apodis*, a single nucleotide polymorphim in exon n and 1 in exon n + 1 was noted (“C” instead of “T” at position 489 (exon n) and at position 498 (exon n + 1) of Nisbet *et al*’s CDS). As for the translated amino acids sequences, they were free of stop codons, identical in all six *Dermanyssus* sequences and very close to *B. microplus* (differing by only three amino acids).

**Figure 2.**
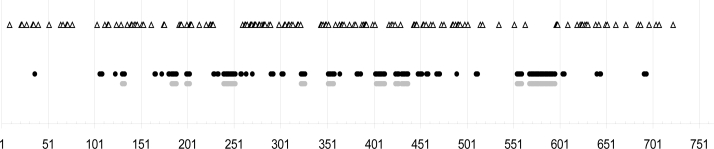
Overview of the distribution of variable sites along the studied Tropomyosin sequence (mutation points and indel regions) on the basis of alignment ISOL_TRO1 (available online as electronic supplementary information (ESI)). Point mutations are figured by open triangles, indels detected in the whole *Dermanyssus* dataset by black dots and indels within *D. gallinae* only by grey dots. Uppercases refer to indel regions within *D. gallinae* as labeled in Appendix z3.

Within the intron, more than 50 sites involve indels, but in many cases a fixed series of 3–5 bp (and even up to 15 bp) is inserted/deleted, resulting in inserted/deleted 35 bp-portions in the whole dataset of *gallinae*-group individuals (see Appendix z3). One region involves some microsatellite motifs, whose number is strongly varying between species, between isolates and within isolates. Sites with indels have been recorded based on alignment ISOL_TRO1 (available online as ESI) and their distribution all along the region under test is located only on intron n and is rather regular when the five focused species are included (Figure 2).The first hundred and the last hundred base pairs are free of indels. Several indels were also detected within *D. gallinae*, which allowed easy separation of alleles in cases of heterozygosity. Important regions with indels have also been noted within *D.longipes* (ID3, ID4, C1, C2, cf. Appendix z3).

Note that indels are found mainly in addition in *D. gallinae* and *D. apodis* (and in subtraction in *D.longipes*, *D. hirundinis*, *D. carpathicus*). As a result, sequences of *D. apodis* and *D. gallinae* populations are longer than in *D. longipes*, *D. hirundinis*, *D. carpathicus* (670–695 bp *vs.* 615–652 bp).

### 3.2. Step 1: Multi-Gene Analyses

The five gene portions have been obtained in six *Dermanyssus* species, two *Ornithonyssus* species and one *Androlaelaps* species. Within *Dermanyssus*, one isolate of *D. hirsutus*, two of *D. apodis*, two of *D. longipes*, three of *D. hirundinis*, three of *D. carpathicus*, nine of *D. gallinae* (of which three of *D. gallinae* special lineage L1, see Roy *et al.* [11]) have been integrated into the analyses. In some cases, two different profiles have been distinguished within some isolates of *D. gallinae* and *D. carpathicus*, due to some intra-isolate variations (mutations and/or indels) in COI and or Tropomyosin sequences. See Appendix 1 for detailed informations about individuals, isolate locations and accession numbers. All genes except Tropomyosin have been found for one additional dermanyssoid genus in EMBL database (*Varroa*) and all except Tropomyosin and 16S for another one (*Tropilaelaps*).

#### 3.2.1. Phylogenetic Interrelationships at the Specific Level

In addition to the six elementary datasets, there were 57 different possible combinations of two to six elementary datasets resulting in 203 different partitioning schemes following Li and Lecointre [17] (see Appendix 4).

On the whole, 24 clades have been observed (see Table 2), and 38 different topologies, of which six exhibit mostly unresolved relationships (see Appendix 5). Bayesian analyses of elementary datasets as well as of the fully combined dataset (psch 203) resulted in similar topologies (results not shown).

The method of Li and Lecointre aims at reducing the stochastic effects of homoplasy, which increases as the dataset’s size decreases, especially in single gene analyses. Nevertheless, the more a clade occurs within partitioning schemes, *i.e.*, the less combination is needed to get it retained, the more it is considered reliable. Two clades were recovered in analysis of each individual dataset, one grouping together all isolates of *Dermanyssus* (π) and the other grouping all isolates of *Ornithonyssus* (σ). Three of the five specific entities with several isolates under test generate clades that occur up to five times per partitioning schemes (*D. apodis*, *D. carpathicus*, *D. hirundinis*), as well as the special lineage L1 of *D. gallinae*. Isolates of *D. gallinae* and of *D. longipes* group together up to 4 times per partitioning scheme.,No contradictory evidence has been noted for *D. gallinae* monophyly (only unresolved relationships lead to unrecovered clade in some cases), whereas contradictions have been noted for *D. longipes* monophyly (clades γ and o in Table 2). Consequently, the analysis of specific level relationships takes separately into account *D. longipes* EN and *D. longipes* PAS, *D. gallinae* L1 and *D. gallinae* non L1. Analyses of elementary datasets 1comb3 (5.8S) and 1comb6 (Tropomyosin exon n and n + 1) did not recover any of the specific entities. The elementary dataset 1comb1 (COI) recovered all species under test. The elementary dataset 1comb2 (16S) recovered all species except *D.gallinae*. The elementary dataset 1comb4 (ITS1 and 2) recovered all species except *D. gallinae* and *D. apodis*. The elementary dataset 1comb5 (Tropomyosin intron n) recovered all species except *D.longipes*.

Finally, relationships between specific entities within *Dermanyssus* are supported by clades with three to four occurrences per partitioning scheme.

By examining the percentage of occurrence of topologies retained based on 50% majority rule consensus trees among the 203 different partitioning schemes and the maximum number of their occurrences, and by considering the level of their resolution, it appears that among the most resolved topologies, Top2, Top17 and Top7 are recurrently encountered with both gaps as missing data and gaps as the fifth state, and they appear up to twice in a single partitioning scheme (see Appendix 4). Of them, the first two are very close to each other. Top2 is fully resolved, with *D. gallinae* L1 as a sister to other *D. gallinae* isolates, whereas Top17 has *D. gallinae* L1 branching from within other isolates of *D. gallinae*. Top7 is close to Top17, except that it presents *D. longipes* as paraphyletic.

**Figure 3.**
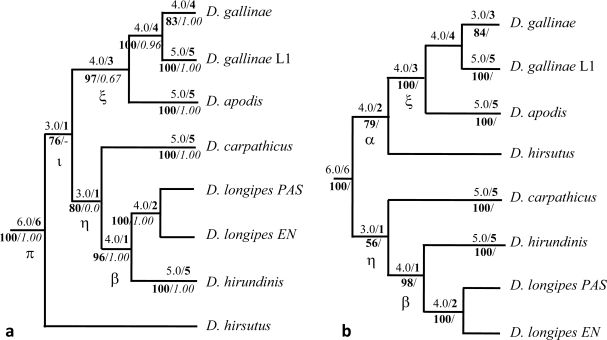
Trees summarizing the most repeated clades encountered in **Step** 1 (multi-gene analyses) at the specific level with the greedy summary method described in Li and Lecointre [17]. Maximum Parsimony criterion, PAUP 4.0. The maximum number of occurrence of the clades and the repetition indices (in bold) are above the branches. The bootstrap supports in the “total evidence” MP analysis are in bold below branches and the Bayesian Posterior Probabilities in the “total evidence” BA analysis are in italic below branches. (**a**) Analyses treating gaps as missing data. (**b**) Analyses treating gaps as the fifth state.

The greedy consensus with gaps as missing data is exactly Top2 (Figure 3). And the greedy consensus obtained with gaps treated as the fifth state is closest to Top 10, *i.e.*, it resembles Top2, with the exception that *D. hirsutus* is transferred from the most basal position to the median position of sister to clade ξ (*D. gallinae* + *D. apodis*). And yet, the total evidence (6comb, partitioning scheme n°203) Bayesian analysis results in Top12 (see Appendix 5), *i.e.*, the strict consensus of both greedy consensuses above.

#### 3.2.2. Specific Characterization Power of Sequences

Among the six elementary datasets, variability and characterization powers strongly differ, depending on the nature of the considered gene and the genomic location (mitochondrial/nuclear genome), mostly following common rules. Observed divergence percentages are presented in Table 3. These elementary datasets may be split into three groups according to their characterization power:

1. COI, 16S and Tropomyosin intron n are very informative at the specific and intraspecific levels, as usually noted in other arthropods.
2. ITS1 and 2 are moderately informative at the specific level and weakly variable within species, contrary to observations of close relatives (Dermanyssoidea: Rhinonyssidae [27,28]), although more similar to other mesostigmatids (Ascoidea: Phytodeiidae [29]).
3. 5.8S and Tropomyosin exon n and n + 1 are insufficiently informative at the specific level and do not show any intraspecific variation, as expected.

### 3.3. Step 2: Multi-Isolate Analyses

Both COI and Tropomyosin portions have been obtained from 211 individuals. Additionally, COI was also obtained in 41 individuals and Tropomyosin in 16 other individuals. This resulted in 56 different COI haplotypes isolated from five species of *Dermanyssus* (three in *D. longipes*, three in *D. hirundinis*, six in *D. carpathicus*, four in *D. apodis*, 35 in *D. gallinae*, one in *D. hirsutus*) and 62 different Tropomyosin alleles (five in *D. longipes*, two in *D. hirundinis*, seven in *D. carpathicus*, two in *D. apodis*, 36 in *D. gallinae*, one in *D. hirsutus*). See Appendix 1 for detailed informations about individuals and isolates.

#### 3.3.1. Additive Information about Phylogenetic Interrelationships

**Figure 4.**
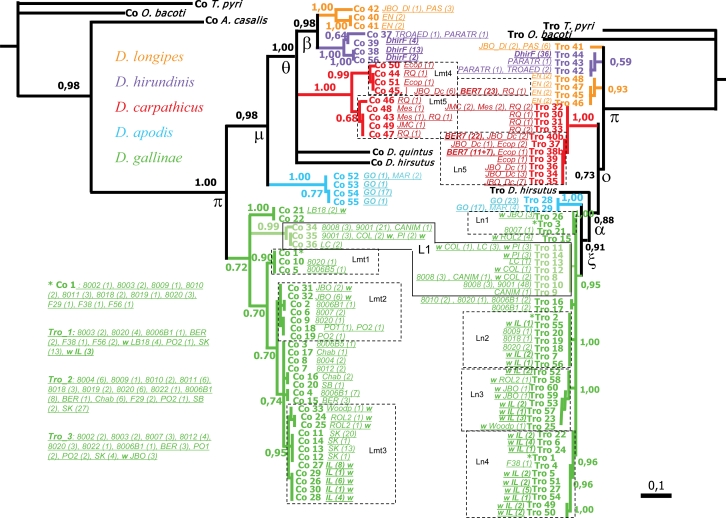
Haplotypic topologies obtained with COI and Tropomyosin sequences and intraspecific variation. COI (*left*) and Tropomyosin (*right*, gaps as missing data) topologies. MrBayes. Bayesian Posterior Probabilities listed at nodes. Mapping of isolates on the topologies is displayed by the names of the simple and focused isolates in italic in front of each haplotype they contain (number of haplotype occurrence in brackets). The comparison of intraspecific variations is above all allowed by mapping the focused isolates sampled in the wild avifauna 
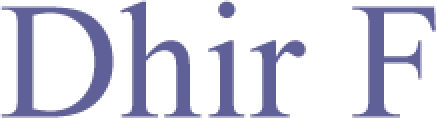
, 
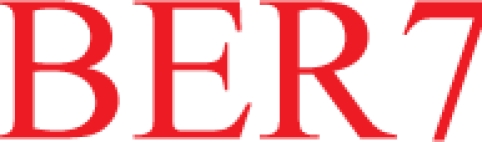
, 
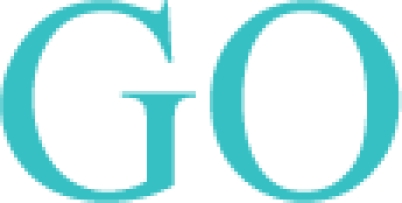
, 
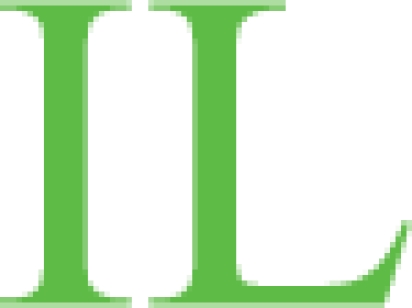
 (names in bold). Within 
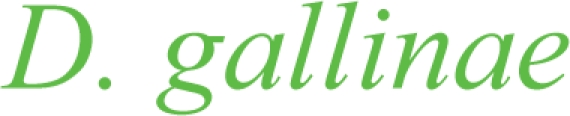
, a w indicates samples from wild avifauna (other species only from the wild avifauna). *Note: here Bayesian topologies are displayed aiming at showing branch length*, *but one must keep in mind that they resulted in interrelationships similar to MP analyses.* Observed host ranges: Passeriformes only: 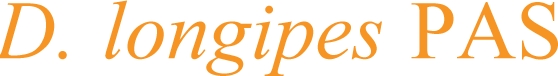 (2 Simple Isolates) on sparrows (Passeridae: *Passer spp.*), 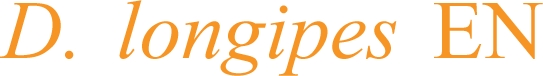 (1 SI) on tits (Paridae: *Parus spp.*), 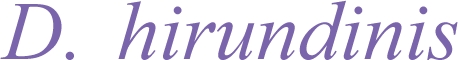 French lineage (6 SI) on swallows (Hirundinidae), 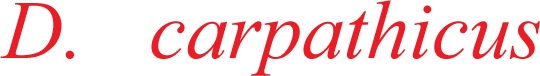 (5 SI) on redstarts (Muscicapidae: *Phoenicurus sp*.) and tits, 
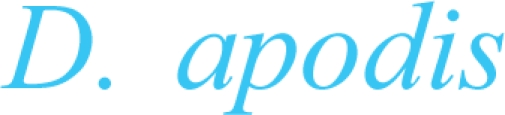
 (1 Focused Isolate, 2 SI) on swifts (Apodidae: *Apus sp.*); Columbiformes: 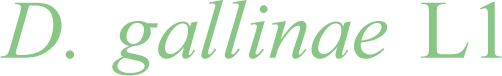 (1 FI, 6 SI) on pigeons (except 2 dead individuals respectively from a swift and an owl nests); Various bird orders: 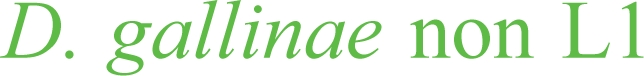 on Coraciiformes, Passeriformes, Galliformes, Apodiformes of which L3 (1 FI) on starlings (Sturnidae: *Sturnus vulgaris*).

Based on COI and Tropomyosin individual matrices and using Maximum parsimony criterion, by treating successively gaps as missing data and as the fifth state for the second one, three different topologies were obtained (50% majority rule consensus trees), one of which is recurrently recovered in multi-gene analyses (Top7, Tropomyosin with gaps as missing data) (Appendix 5) and two of which do not exactly match with any of the 38 topologies retained in **Step** 1 (COI and Tropomyosin with gaps as the fifth state). Indels-only MP analysis reveals a rather strong phylogenetic signal of intronic indels of Tropomyosin, since topologies obtained using indels-only and gaps as missing data generate similar topologies, although slightly less resolved in the former (Appendix 6). Bayesian analyses (Figure 4a) resulted in the same topologies as Maximum parsimony analyses with gaps as missing data, with the exception that one node more is dichotomic with COI (node grouping together non *gallinae* species) and one node is lost with Tropomyosin (MP node grouping together basal haplotypes Tro_41(Tro_42, 43, 44) (see Appendix 6). With gaps as the fifth state, the MP analysis of Tropomyosin matrix results in the same topology as gaps as missing data Bayesian’s with the exception that it contains four more internal dichotomic nodes (Figure 4b). These additional resolved relationships reinforce the scale-like shape of the toplogy. No Bayesian analysis with gaps as the fifth state may be performed using MrBayes.

The monophyly of dermanyssid species under test as well as the monophyly of specific entities *D. carpathicus*, *D. apodis* and *D. hirundinis* are strongly supported. The monophyly of *D. gallinae* is also supported, except in the BA analysis based on COI. *D. gallinae* special lineage L1 is recovered in all analyses, with support, except in Tropomyosin indels-only MP analysis. The monophyly of *D. longipes* is recovered only in COI analyses, not in any of the Tropomyosin-based analyses. In the latter, the two isolates of *D. longipes* under test appear as paraphyletic basal entities (see clade o Table 2, Figure 4).

Pairwise relationships between species of *Dermanyssus* are roughly similar between mitochondrial and nuclear topologies, but an important paraphyly is noted between both loci. Based on both loci, a scale-like topology is retained, in which *D. apodis* appears to be the closest species to *D. gallinae*, with *D. hirsutus*, then *D. carpathicus*, then *D. hirundinis* and *D. longipes* as more distant species. But the common ancestor rooting appears to act between *D. gallinae* and *D. apodis* in mitochondrial analyses, and between *D. longipes* EN and *D. longipes* PAS in nuclear topologies.

#### 3.3.2. Differentiation within Previously Delimited Specific Entities

Intraspecific variability appears very different according to the considered species within *Dermanyssus*. The simple observed number of haplotypes, along with divergence percentages noted within focused isolates show a marked opposition between *D. gallinae* and others (Figure 4a). Indeed, when considering focused isolates sampled from nests of wild birds (populations living in comparable environments, *i.e.*, here non anthropized), *D. gallinae* appears to possess many more Tropomyosin haplotypes, with more divergence than other species under test, (Figure 4a, in bold: focused isolates from wild bird nests in *D. apodis*, *D. carpathicus*, *D. gallinae* and *D. hirundinis*).

The average number of differences K in COI haplotypes for focused isolates (Table 4) is the lowest in all focused isolates (0–1.1), except in *D. hirundinis* pseudo-isolate DhirF (K = 4.6). This exception was expected since this latter group of individuals was sampled from across France, as opposed to the five true focused isolates. This difference highlights a slight differentiation between isolates of various geographical origins at least in French *D. hirundinis*. The differentiation between isolates within *D. gallinae* is much sharper (up to 9% divergence in COI haplotypes between simple and/or focused isolates of *D. gallinae* non L1 from various geographic origins).

The average number of differences K in Tropomyosin haplotypes for focused isolates is completely null in *D. hirundinis* pseudo-isolate DhirF and very low in *D. apodis* and *D. carpathicus* focused isolates (GO 4.51, BER7 1.83 with gaps considered). In contrast, it is important in *D. gallinae* focused isolates under test (SK 14.5, IL 32.5, corresponding to up to 7% divergence), except in *D. gallinae* L1 (nul as in *D. hirundinis*).

Both *Dermanyssus apodis* focused isolate GO and *D. carpathicus* focused isolate BER7 show low diversity in both mitochondrial and nuclear genes. The inter-isolate intraspecific variation within *D. apodis* is not clearly estimable based on the present dataset, as only one focused isolate and one simple isolate were sequenced (France). Anyway, the simple isolate involved here of *D. apodis* (MAR, Center France) provided the same haplotypes as the focused isolate GO in both loci, and three individuals from Corsica provided a COI diverging by only 3–4 nucleotides from GO and MAR (*i.e.*, 0.5–0.7%; acc. n^o^ FN398146, not included in present analyses). These are sizeable insights of the low variability in COI sequences within *D. apodis*, which appears to be independent of geographical location, whereas a slightly higher divergence is noted in Tropomyosin intron n. As for *D. carpathicu*s, five simple isolates were integrated in analyses in addition to the focused isolate BER7, which reveal relatively low inter-isolate variation in Tropomyosin intron n and a slightly larger range of values in COI (see branch lengths in Figure 4a). *D. gallinae* not only possesses by far the highest number of haplotypes in both genes under test in **Step 2**, but also it shows the highest intra-isolate and inter-isolate sequence diversity.

*D. gallinae* IL and SK show high heterozygosity in Tropomyosin sequences, including numerous allelic indels (Table 4). Heterozygosity is much lower in *D. apodis* GO, but involves also allelic indels, whereas it is low in *D. carpathicus* BER7 and does not include any indels (see Table 4). Overall, intraspecific as well as intra-isolate variability in the Tropomyosin locus is very important in *D. gallinae* non L1, in contrast to *D. apodis*, *D. carpathicus*, *D. hirundinis* and *D. gallinae* L1 (see Tables 3 and 4).

The ratio of external branch length / internal branch length in mitochondrial monophyletic groups and the comparison with that of corresponding monophylies shows Tropomyosin is already deeply structured. In the COI gene tree, branch lengths between clades are much longer than intraspecific branch lengths in the species which are strictly encountered in the wild avifauna, but not in the “*gallinae* complex” [10]. All entities with a ratio >2 recovered monophyly in the nuclear topology (e.g., species *D. apodis* 121.0, *D. carpathicus* 5.8, *D. hirundinis* 2.2). On the other hand, none of entities with a ratio <1.5 recovered monophyly in the nuclear topology (e.g., *D. longipes* 1.4), except *D. gallinae* (1.1).

Support values, along with some branch length ratios, draw the attention to some important lineages within *D. gallinae* and *D. carpathicus*. As a result, the examination of the most supported clades led to focus on 3 COI within *D. gallinae* clades, 2 COI within *D. carpathicus* clades, 4 Tpm within *D.gallinae* clades and 1 Tpm within *D. carpathicus* clade (Table 5a).

By mapping isolates on each supported clade and comparing them between mitochondrial and nuclear topologies (Figure 4a, Table 5b), the special lineage L1 in *D. gallinae* appears to be completely isolated, with monophyly rather recently reached (short basal branch length in Tpm Bayesian topology). Isolates of special lineage L1 [11] group together in both mitochondrial and nuclear analyses (both MP and BA). However, they do not group as sister to the remaining *D. gallinae* populations, but arise from within the *D. gallinae* complex. They diverge by 10–13% from other *D.gallinae* lineages in COI (1–2% between each another within L1) and by only two mutational differences (no particular indel) from some other *D. gallinae* isolates in Tropomyosin. They are more differentiated in Tropomyosin from *D. gallinae* focused isolates (9001 *vs. D. gallinae* s. str. Fst 0.44–0.65) than they are between each other (Fst 0.00–0.32), but not as much as from other species (Fst 0.97 against *D. apodis*).

Within species, no other mitochondrial lineages are monophyletic in the nuclear topologies, which are naturally expectable within species and are likely to result from reticulation, as well as recombination, but may also result partly from incomplete lineage sorting. Nevertheless, the mitochondrial clade Lmt3 and the nuclear clade Ln3 share three different isolates sampled in the wild avifauna, in different places and bird host species (two thirds in each clade). This might represent a lineage incompletely isolated. Within *D. carpathicus*, the clades Lmt4 and Ln5 also share an important portion of isolates and could represent an incompletely isolated lineage.

## 4. Discussion

### 4.1. Specific Characterization Power of Sequences

The results integrating the newly developed nuclear marker Tropomyosin confirm reproductive isolation, and consequently specific status of *D. carpathicus*, *D. hirundinis*, *D. apodis* and *D. gallinae. Dermanyssus carpathicus* and *D. hirsutus* appear to be strongly differentiated from each other and from other *Dermanyssus* species in all genes except the small portions composed of Tropomyosin exon n and n + 1 (see Table 3). *Dermanyssus apodis* is strongly differentiated from other *Dermanyssus* species in both mitochondrial datasets and in the nuclear Tropomyosin datasets (both intron and exons), whereas it is very weakly differentiated based on ITS1 and 2 and not differentiated in 5.8S. On the other hand, special lineage L1, although less differentiated than *D. apodis* in other genes, appears more different in the ITS sequences.

Moreover, although the reproductive isolation of *D. longipes* isolates seems to be confirmed, this entity is revealed to be diphyletic. Two contradictory clades against *D. longipes* PAS + EN among the different partitioning schemes tested in **Step** 1 and a paraphyletic position of *D. longipes’* Tpm sequences in two well supported clades in the MP analysis of **Step 2** with gaps as the fifth state shows that this entity is composed of two different species. The characterization of both these lineages remains imprecise, since they are each represented here by one or two isolates from a single site. However, as noted in Roy *et al.* [11], sequences of ITS1 and 2 published by Brännström *et al.* [30] from mites sampled in Sweden on different bird species are exactly identical to ITS in *D. longipes* EN, whereas ITS sequences of *D. longipes* PAS diverge by 2%. Given the very low amount of interspecific divergence in ITS sequences within *Dermanyssus*, it is very likely that the Swedish isolates belong to the same specific entity as present EN isolates. Of the two *D. longipes* lineages, *D. longipes* PAS appears as closer to *D. hirundinis* (Figure 4b). Since this isolate has been sampled near the type locality and in the type host genus, it is to be considered the name bearing species. As a result, the lineage EN will be referred to as *Dermanyssus sp.* EN in the remaining text.

As for *D. gallinae* special lineage L1 [11], it appears as an isolated lineage with strong support. The monophyly of this lineage and the *gallinae* complex is also supported. Nevertheless, its position within the *gallinae* complex or as a sister to it remains unclear.

Multiple functional copies of EF-1α have been found in various Arthropoda [31–33] including in some cases both functional and non functional copies in single species [34]. Moreover, Hedin and Maddison [25] have shown the presence of intronless non functional copies among multiple copies in a portion overlapping the region studied here within the genus *Habronattus* (Aranea: Salticidae) (positions 529–1005 of the *H. melpomene* CDS). Intronless copies seem to be evolving under relaxed functional constraints. Goetze [34] published a similar report on Crustaceans with exons of putative functional / non functional sequences almost undistinguishable from each other.

In the present datasets, it is worth noting that there is no intron in any of the EF-1α portions under test, although several other Arthropoda show introns precisely within this portion, especially some Arachnida: between positions 771–772 (836–837 of the whole published sequence), a 167 bp intron is present in *Mecaphesa* sp. (Arachnida:Araneae:Araneomorphae: Thomisidae, FJ590835), a 124 bp intron in *Habronattus* (Arachnida: Araneae: Salticidae, AF477231) and a 147 bp intron in *Haplochthonius* simplex (Arachnida: Acari: Oribatida: Haplochthoniidae, GQ398254). And this position also possesses an intron in some insects (Coleoptera, Hymenoptera) [35] and some crustaceans (Calanoidea: Eucalanidae) [34]. Not to mention that at least two other positions in the present region possess introns in the same crustaceans and in some Diptera [34].

The intronless nature of presently tested sequences may suggest that they are not functional and evolve as pseudogenes. Of course, there are no stop codons in any of obtained sequences, but there are a few non silent mutations either in sequences of clade EF_A or in clade EF_B. And yet EF-1α has already revealed some difficulties in the application to deeper systematic relationships in Mesostigmata [36], and present inferences show largely unsupported internal relationships within Parasitiformes, including a few incongruences if compared with their phylogenetic relationships robustly established by Klompen *et al.* [37] based on rRNAs. Here, EF-1α copies position in the phylogenetic reconstructions of mites based on this gene (Figure 1) demonstrates that gene duplication has occurred anteriorly to the split between Macronyssidae and Dermanyssidae. The date of the duplication may be posterior to the split between dermanyssoid and other Mesostigmata according to the position of double copies in the large phylogenetic reconstruction represented in Figure 1. But further exploration would be needed in order to check this.

All the more, it is not unlikely that other difficulties are generated by multiple copies, including once more non functional ones at a lower taxonomic level within clade EF_B (Figure 1). Indeed, the structure remains unclear, as some sequences of outgroups and of ingroups group together in a distal position. A potentially more recent duplication event may have occurred, but on which present dataset is not sufficient to get any explanation, once more likely because of some failure in amplifying all copies. In any case, this gene region does not seem to be appropriate for interspecific investigations within *Dermanyssus*, nor for interfamily exploration within Dermanyssoidea. And this is in accordance with results of Goetze [34] in Calanoidea (Crustacea), where paralogous gene copies are likely to be problematic for phylogenetic studies, especially at the species level.

### 4.2. Phylogenetic Interrelationships at the Specific Level

In the present study, the biclade topology described by Roy *et al.* [10] based on mitochondrial DNA, opposing the group of specialist species (*D. carpathicus* + *D. longipes + Dermanyssus* sp. *EN* + *D. hirundinis* + *D. apodis* + *hirsutus*-group) to the synanthropic *gallinae* complex is not supported by most of results (Figures 3 and 4). It appears it is a topology retained solely based on COI alone, only with the enlarged set of isolates in **Step 2** (Top5, see Appendix 5), and with rather low support values at internal nodes.

Topologies retained in present multi-gene analyses show a biclade structure with gaps as the fifth state and a semi-biclade structure with gaps as missing data (clades ξ and η, see Figure 3), but in any cases, the two clades do not oppose non synanthropic species to the synanthropic one: the generalist and synanthropic *D. gallinae* in both greedy consensuses groups with *D. apodis* (clade ξ), which is a swift specialist and is absent from farms, with correct support (max. occurrences per partitioning scheme 4.0, final repetition index 3).

The rooting of the common ancestor is problematic. When using the enlarged set of isolates (**Step 2**, multi-isolates analyses), pairwise specific relationships appear similar to multi-gene topologies, but a noticeable paraphyly between mitochondrial and nuclear analyses arises (see Table 2, Figures 3 and 4). In multi-gene greedy consensuses, the position of *D. hirsutus* remains unclear (basal with gaps as missing data, a sister to the clade ξ, *i.e.*, *D. gallinae* + *D. apodis*) with gaps as the fifth state. At the specific level, in multi-isolate analyses, scale-like topologies are retained, which are mutually and symmetrically paraphyletic, with *D. gallinae* as the basal entity in mt-COI analyses and *D. longipes*, *D.* sp. EN and *D. hirundinis* as the basal entity in nuclear Tpm analyses (Figure 4). In Roy *et al.* [11], a slight incongruence was vaguely suggested between mitochondrial and nuclear topologies, but the nuclear gene region used in this study was not variable enough to establish it firmly (ITS1–5.8S-ITS2).

The species tree is often not identical to the gene tree [38], due to several potential causes. Reduced effective population size (Ne) in mitochondrial DNA compared to nuclear DNA (a quarter in most sexually reproductive organisms, a third in the present case, due to haplodiploidy) often causes a high mutation rate in mitochondrial DNA resulting in less resolved internal relationship in mitochondrial topologies than in nuclear topologies. As a result, in mitochondrial gene trees, the most recurrent bias is due to homoplasy, and inconsistencies in nuclear gene trees are due to the stochastic effects of lineage sorting. Additionally, interspecific hybridization in some cases may induce reticulation [39].

The choice of a more appropriate gene tree for relationships between species requires some attention. Some authors considered more appropriate mitochondrial markers for inferring phylogenies at the specific level [40,41] but they were dealing with organisms with smaller numbers of generations per year (birds, rodents; *D. gallinae in natura*, around 15 gen/y, in farms, >200 gen/y), and thus with likely reduced mutation rates in both mitochondrial and nuclear genomes. McCracken *et al.* [39] recommended a balanced approach, taking into account both advantages and flaws due to different effective population size Ne and considering first whether independent gene trees are adequately resolved and then whether those trees are congruent with the species history.

In the present case, a bias may come from the outgroups: no very close outgroup has been integrated into the analyses, because no individual of *Liponyssoides*, the only other genus within Dermanyssidae, has been found despite an intense screening of potential hosts. As a result, only other Dermanyssoid families are represented as outgroups (Macronyssidae, Laelapidae, Varroidae). Bayesian topologies reveal important distances between in- and outgroups, which might explain some difficulties in trying to clearly locate the ancestral rooting point. The different genera integrated here as outgroups are rather diverse, and provide good coverage across Dermanyssoidea. Since there are currently no published phylogenetic hypotheses for Dermanyssoidea, it is unknown what is the most appropriate outgroup, which is why it was important to have a diverse and *a priori* paraphyletic set of outgroups. Additionally, the results indicate that some clades are better, or more reliable than others, and consequently may help for the choice of the most reliable topology: clade η (recovered in both greedy summaries in multi-gene analyses, see Table 2, Figure 3) is not recovered in any multi-isolate analyses, and yet its maximum number of occurrence in partitioning schemes is 3.0 and its repetition index is 1 in multi-gene analyses (both gaps as missing data and as the fifth state). On the other hand, clade ξ (*D. gallinae + D. apodis*) receives higher support values in both the multi-gene MP analyses (4.0/3). And yet this clade is present in Tpm multi-isolate analyses, whereas absent in mt-COI multi-isolate analysis. Moreover, nuclear results show much less homoplasy (cf. CI and RI in Table 6) than mitochondrial results. And the most supported internal nodes in multi-gene analyses also occur in Tropomyosin-based multi-isolate analyses. Finally, a comparable topology was already suggested based on ITS sequences by Roy *et al.* [11], but the low amount of DNA divergence in this sequence did not provide enough resolution. It was only vaguely suggested and the few resolved internal nodes were not supported.

The basal position of *D. hirsutus* in the greedy consensus for multi-gene analysis treating gaps as missing data may be due to the lack of indel information present in Tropomyosin intron n. Kawakita *et al.* [24] have shown that inclusion of intronic gap characters consistently contribute to phylogenetic reconstruction, at least at lower taxonomic levels. Results of indels alone analysis (Appendix 6) strongly suggest that Tpm intronic indels contains important and consistent phylogenetic information and represents interesting complements to the information present in gap-free corresponding intronic portions. And yet, the basal position of *D. hirsutus* is less supported in gaps as missing data analyses than its median position in gaps as the fifth state (see Figure 3). This strongly suggests that the split between *hirsutus* group and *gallinae* group of Moss [42] is not valid.

### 4.3. Generalist: A Derived or Ancestral Condition?

Non *gallinae* species are more or less specialized, whereas *D. gallinae* has been encountered in nine different bird orders in France by Roy *et al.* [10]. *Dermanyssus apodis* and the French isolates of *D. hirundinis* are rather strict specialists, as exclusively encountered on the genus *Apus* for the former and in the family Hirundinidae for the latter in France in present study as well as in Roy *et al.* [10,11]. Looking like intermediate entities on the host specialization point of view, *Dermanyssus* sp. EN and *D. carpathicus* are known from two to three different bird families, but both within Passeriformes: *Dermanyssus* sp. EN on Paridae (in France, present data), Muscicapidae and Sylviidae (in Sweden [30]), *D. carpathicus* on Paridae and Muscicapidae (present data). The two incompletely isolated lineages within *D. carpathicus* (Lmt4 and Lmt5) do not appear to be restricted to any of the two passeriform families (see Appendix 1). As for *Dermanyssus* sp. EN, it is likely to be a moderate specialist, as is *D. carpathicus*, *i.e.*, parasitizing various bird passeriform families, which are not as conspicuously distant between each other as are *D. gallinae’*s hosts (bird taxonomic considerations follow Peterson’s classification [46]), but additional samples are needed to establish it clearly. Finally, not enough isolates of *D. longipes s. str.* are available here to estimate the host range of this species.

In all topologies except the multi-isolate COI analyses, a derived state of generalist *vs.* specialist condition is noted: *D. gallinae*, the only generalist species in present study [10] is in a distal position. This may indicate, as already shown in the fish ectoparasite *Lamellodiscus* (Trematoda: Monogenea) [2], as well as in some free-living insects such as bees [3] and springtails (Collembola) [4], that, contrary to usual expectations, the specialist condition does not appear as a “dead-end”. *Dermanyssus gallinae*, the generalist species, is, if not the more derived species, at least one of the distal ones with *D. apodis*, whereas basal positions are occupied by specialist species. And it is worth noting that special lineage L1 of *D. gallinae* has been almost solely encountered in pigeon nests: only two individuals have been found, both dead, in other birds nests (JGC1 and GO8 in Roy *et al.* [11]), the one in a bird of prey’s nest, the second, in a black swift’s nest, which is known to be a frequent competitor of pigeons for nest site. Consequently, both these non-pigeon occurrences are likely fortuitous. The precise position of L1 is not absolutely clear, either basal to non L1 *D. gallinae*, or branching from within *D. gallinae*. In any case, it appears to be currently reproductively isolated and seems to represent a species recently isolated (no morphological difference with non L1 *D. gallinae*, low divergence based on nuclear loci Tropomyosin intron n and ITS1 and 2).

The loose relation of *Dermanyssus* micropredator mites to their host/prey should have led one to expect a fundamentally wider host range in *Dermanyssus.* And yet, the case of bees’ ancestral specialist condition as evidenced by Danforth *et al.* [3] is the most comparable: despite nectar and pollen collectors do not live on nor develop on their resource-plant, the placement of a paraphyletic Melittidae s.l. at the base of the phylogeny indicates that host–plant specialization is the primitive state.

### 4.4. Reticulate Evolution or Gradual Specialization/Speciation?

Interestingly, the important difference observed in the haplotype variability between species does not seem to be due to the bird’s ecology. Apparently, an important intermingling involving a large number of Tpm haplotypes in *D. gallinae* is noticeable within a colony of starlings (focused isolate IL, Table 4, Figure 4a). But this does not seem to be solely correlated to the bird’s ecology, as we noted the exact contrary in *D. apodis* individuals from a colony of swiftlets (focused isolate GO, two Tropomyosin haplotypes) and in *D. hirundinis* from 6 separate French colonies of barn swallows (focused pseudo-isolate DhirF, one Tropomyosin haplotype). Thus starlings, swiftlets and swallows reuse nests of other pairs in the same colony from one year to another (O. Caparros, CRBPO, MNHN, pers. comm.). This could suggest that *D. gallinae* would have the opportunity to move from one nest to another by phoresy on the bird host (see above). But the ability of *D. apodis* to get transferred by the birds has also been shown by several adult females directly sampled on (flying) hosts [10, 11]. The conspicuous stability of both genes under test in **Step 2** in *D. apodis*, *D. carpathicus* and *D. hirundinis* strongly contrasts with their variability in *D. gallinae*, inter-isolate in COI and inter- as well as intra-isolate in the nuclear Tropomyosin. This suggests that these represent two types of species that are intrinsically very different, although sister to each other.

This difference between *Dermanyssus* species might be a consequence of interspecific hybridizations within the *D. gallinae* complex. The monophyly of *D. gallinae* is not doubted here as it is recovered in all topologies. Nevertheless, this is supported by few synapomorphies and the ratio external/internal mt branch length is below (1.1) the lower limit of mt clades to which monophyletic nuclear clades correspond (<1.5). This could suggest that the date of this coalescence occurred much later than coalescences for other species. But the distance of the coalescent node for *D. gallinae* to the common ancestor with the closest species *D. apodis* (considering that the right arrangement is recovered by Tropomyosin multi-isolate topologies) is also by far the shortest (see Bayesian topology, Figure 4a). Either the two loci under test in *D. apodis*, *D. hirsutus* and *D. carpathicus* have evolved faster than in *D. gallinae*, or the apparent low rate of evolution within the latter is rather an artifact due to a radiation followed by interbreeding. The latter alternative would appear much more credible since (1) the number of nuclear segregating sites and or indel sites is significantly the highest within *D.gallinae* focused isolates as opposed to focused isolates or even pseudo-isolates in others species, which is suggestive of the assemblage of formerly highly divergent haplotypes (Table 4), (2) many Tpm genotypes of heterozygous individuals show high divergence percentages within *D. gallinae*, (3) the specialist lineage L1 with similar stability in both COI and Tpm sequences to other species’ is branching from within the widely generalist *D. gallinae* in multi-isolates analyses and basal species are at least moderate specialists, (4) clades Lmt3 and Ln3 share a major part of their isolates and are strongly supported in mitochondrial and nuclear analyses (indels alone and gaps not considered for the nuclear clade Ln3), whereas showing important interbreeding. These two clades could provide evidence of a vestigial completely isolated lineage L3, whose speciation has been in reversion due to subsequent interbreeding.

All that suggests that *D. gallinae* appears as a complex of species, which would have interbred soon after speciation, and just before pre- or post-zygotic incompatibility has been installed. Such a reversal of speciation has been shown to occur in various organisms in case of habitat defragmentation, which induce relaxed divergent selection and increased gene flow because of loss of ecological barriers [47]. Naturally, this potential scenario is no more than a hypothesis, which needs some complementary investigations before being clearly established in *D. gallinae*. In such a case, the idea evoked by Futuyma and Moreno [1] that specialization and rate of diversification are positively correlated would not be so clearly contradicted by the differential variability depending on specialization within *Dermanyssus.* The increased molecular diversity in the generalist species would not seem to be due to continuous and intrinsic evolution, but a consequence of the diversification of specialist lineages and consecutive intermingling. It is also worth noting that the only other species with allelic divergence involving some indels in Tropomyosin is *D. apodis*, the sister to *D. gallinae*. This suggests that the clade ξ might be particularly prone to hybridize, more than close groups, as it has been shown in some plant groups [48].

Of course, observed differential divergence levels between lineages within *D. gallinae* complex might suggest a continuum of genetic divergence from sympatric host races to species as recently evidenced in the pea aphid complex [51]. But phylogenetic inferences obtained in present studies, by integrating several sister species and so providing us with a historical scenario, strongly suggests that the gradient of genetic divergence observed here is evolving from weak variability to high variability in the tree of 6 species of *Dermanyssus*, not the reverse pattern. It’s likely the present markers are too slowly evolving to detect new speciation events possibly in process within the *gallinae* complex. But from the present data it is to be concluded that the generalist condition of *D. gallinae* has been arising from a specialist ancestor.

## 5. Conclusion

Although reticulate evolution remains difficult to disentangle using phylogenetic tools, comparisons of multi-gene analyses and further analyses of the differentiation in two optimal markers led us to reach some consistent genealogical information at the specific level as well as some insights on more recent isolations and to evidence different evolutionary processes between species.

Intronic nuclear region in Tropomyosin revealed precious phylogenetic signal, at least within *Dermanyssus* and constitutes a new potentially interesting marker for phylogenetic explorations of Mesostigmata and other arhropods. ITS 1 and 2 do not contain information at intraspecific levels and offer very little interspecific characterization in this genus contrary to some related mite groups. Mitochondrial gene regions are information rich at distal levels, but poorly adapted to resolve internal relationships within *Dermanyssus.* Finally, as already shown in other arthropods, EF-1alpha does not seem to be appropriate for any phylogenetic/diagnostic exploration within this genus due to some duplication events.

The special lineage L1 seems to represent a cryptic species. The position of *D. hirsutus* appears to be within the *gallinae* group of Moss.

The generalist condition revealed to be derived within *Dermanyssus*, and might result from the hybridization of different specialist entities. The specialist condition of L1 among the generalist lineage complex could represent the sign of its vestigial nature (a remaining lineage isolated during a basal radiation).

Among perspectives, a hypothesis to be tested in the future is: the generalist condition of the *gallinae* complex might result from hybridizations between different specialist lineages which were each primarily restricted to different and narrow bird groups. Testing predictions obtained with mt *versus* nDNA by the mean of an Approximate Bayesian Computation (ABC) method should allow us to check the assumption of hybridization within *Dermanyssus*. Moreover, it would be interesting to use faster evolving markers such as microsatellites and population assignment as in Peccoud *et al.* [51] in order to determine whether new host races are not recently isolating from each other within the *gallinae* complex.

## List of Electronic Supplementary Information (ESI)

ISOL_TRO1 (nexus DNA alignment of Tropomyosin partial exon n, complete intron n, partial exon n + 1 upon which the matrix of encoded indels has been established – see Appendix z3).

Appendix 1 (Sampling and EMBL information for the populations under test in present study)

Appendix 2 (Primer sequences)

Appendix 3 (matrix of encoded Indels in Tropomyosin)

Appendix 4 (The different partitioning schemes for the multi-gene analyses and estimation of repetition index)

Appendix 5 (The different topologies obtained at the specific level within *Dermanyssus* in multi-gene analyses and in multi-isolate analyses)

Appendix 6 (MP topologies obtained in **Step 2** not shown in Figure 4)

## Figures and Tables

**Table 1.** Elementary datasets.

	**mtDNA**	**nDNA**
rRNA	1comb2 (16S)	1comb3 (5.8S)
Internal Transcribed Spacers		1comb4 (ITS1 & ITS2)
Protein coding genes	1comb1 (COI)	1comb6 (Tropomyosin exon n & n + 1, EF-1α)
Intron		1comb5 (Tropomyosin intron n)

**Table 2.** List, composition and support values of clades recovered in any analyses in the **Steps 1** and **2**.

Isolate names												multi-gene MP analyses				multi-gene MP COI analysis	multi-gene BA COI analysis	multi-gene MP Tropomyosin analysis			multi-gene BA Tropomyosin analysis

Clade	COL,COL2,LC,LC2,PI	Chab, Chab2, LB18, PO2, ROL1, ROL2, SK, SK2, SK3, Woodp	MAR,GO	JBO59, 5, RQ, RQ2	Adhirun, HR, OC	PAS	ENVL08_3	ADhirs	Tropilaelaps	JBO_Os,FS5,FS6,OSBM	OB	Gaps as missing data		Gaps as the fifth state				Gaps as missing data	Gaps as the fifth state	Gaps alone	Gaps as missing data

												max. no of occurrence per partitioning scheme	Final repetition index	max. no of occurrence per partitioning scheme	Final repetition index	presence/absence of clades	presence/absence of clades	presence/absence of clades	presence/absence of clades	presence/absence of clades	presence/absence of clades
*D. gallinae*	x	x										4	4	4	4	1	1	1	1	1	1
*D. gallinae L1*	x											5	5	5	5	1	1	1	1	1	1
*D. gallinae non L1*		x										4	4	3	3	1	1	0	0	0	0
*D. apodis*			x									5	5	5	5	1	1	1	1	1	1
*D. hirundinis*					x							5	5	5	5	1	1	1	1	1	1
*D. carpathicus*				x								5	5	5	5	1	1	1	1	1	1
*D. longipes*						x	x					4	2	4	3	1	1	0	0	0	0
*O. sylviarum*										x		*5*	5	*5*	*5*	-	-	-	-	-	-
x	x	x	x									4	3	4	3	0	0	1	1	1	1
b					x	x	x					4	1	4	1	0	0	0	0	0	0
g					x	x						2	−2	2	−2	0	0	0	0	0	0
d				x	x							3	−1	3	−1	0	0	0	0	0	0
h				x	x	x	x					3	1	3	1	0	0	0	0	0	0
q				x	x	x	x	x				1	−2	0	−	1	1	0	0	0	0
**a**	x	x	x					x				0	-	4	2	0	0	1	1	1	1
m	x	x	x		x	x	x					1	−1	2	−2	0	0	0	0	0	0
i	x	x	x	x	x	x	x					3	1	2	−2	0	0	0	0	0	0
n	x	x	x		x	x	x	x				2	−1	2	−1	0	0	0	0	0	0
y	x	x		x	x	x	x	x				1	−3	1	−3	0	0	0	0	0	0
z			x	x	x	x	x	x				0	-	0	-	1	1	0	0	0	0
o	x	x	x	x	x	x		x				0	-	1	−1	0	0	0	1	1	0
p	x	x	x	x	x	x	x	x				6	6	6	6	0	0	0	0	0	0
r	x	x	x	x	x	x	x	x	x			2	2	0	-	0	0	0	0	0	0
s										x	x	6	6	5	5	1	1	1	1	1	1
f	x	x	x	x				x				0	-	0	-	0	0	0	1	0	1
m			x	x	x	x	x	x				0	-	0	-	1	1	0	0	0	0

**Table 3.** Percentages of divergence in the six elementary datasets defined in **Step** 1.

	**Between species**	**Within species**	**Between *D. gallinae* L1 and *D. gallinae* non L1**	**Between*D. longipes*EN and*D. longipes*PAS**	**Remarks**
**COI**	9–18%	0–5% (rarely up to 9%)	10–12%	5%	
**16 S**	10–16%	0–4%	6–7%	3%	
**5.8 S**	0–3%	0%	0%	0%	only *D. carpathicus* and *D. hirsutus* differenciated from each other and from others.
**ITS1 and 2**	2–5% (rarely up to 9%)	1%	3%	2%	9% between *D. hirsutus* and other *Dermanyssus* species only - More than a half: 2–3% – 1% in case between *D. apodis* and *D. gallinae* non L1, and between *D. hirundinis* and *D. longipes* EN
**Tropomyosin intron n**	8–20%	0–6%	2–6%	4%	
**Tropomyosin exon n and n + 1**	cf. remarks	0%	0%	0%	Very small portion (25 pb). 2 point mutations in *D. apodis vs.* other *Dermanyssus* species. 1–2 point mutations + 1 indel *Ornithonyssus vs. Dermanyssus*

**Table 4.** Information on sequences variability in focused isolates. Genotypic and heterozygosity variability in focused isolates for Tropomyosin exon n, intron n and exon n + 1 (DnaSP, Arlequin). n refers to the number of sequences under test, G to the genotype number, HET(obs) to the observed % of heterozygozity, Allind to the presence (P)/absence (A) of indels between alleles, D% ind to the observed maximum % of divergence between haplotypes when considering indels, D% no ind to the observed maximum % of divergence between haplotypes whithout considering any indels, S to the number of segregating sites, Sind to the number of polymorphic/indel/missing sites, h to the allele/haplotype number, Hd to the haplotype diversity, K to the average number of differences.

	**Tropomyosin**													**COI**				
**Isolate/population**	**n**	**G**	**HET(observed)**	**Allind**	**D% ind**	**D% no in**	**S(observed)**	**Sind (observed)**	**h**	**h (gap as a fifth state)**	**Hd**	**K (gap as missing data)**	**K (gap as a fifth state)**	**n**	**S**	**h**	**Hd**	**K**
***D. gallinae*SK**	44	4	0.41	P	0.03	0.02	14	41	3	3	0.54	4.60	14.46	24	3	4	0.31	0.33
***D. gallinae*IL**	38	18	0.88	P	0.06	0.03	37	116	18	18	0.94	9.56	31.88	20	4	5	0.74	1.06
***D. gallinae*L1 9001**	48	1	-	-	0.00	0.00	0	0	1	1	0.00	0.00	0.00	24	8	2	0.23	1.83
***D. apodis*GO**	40	3	0.14	P	0.01	0.01	4	9	2	2	0.50	2.01	4.51	20	3	4	0.28	0.39
***D. carpathicus*BER7**	40	5	0.70	A	0.01	0.01	4	4	3	4	0.61	1.83	1.83	24	0	1	0.00	0
***D. hirundinis*DhirF**	36	1	-	-	0.00	0.00	0	0	1	1	0.00	0.00	0.00	21	15	4	0.53	4.59
***D. gallinae*SK**	44	4	0.41	P	0.03	0.02	14	41	3	3	0.54	4.60	14.46	24	3	4	0.31	0.33
***D. gallinae*IL**	38	18	0.88	P	0.06	0.03	37	116	18	18	0.94	9.56	31.88	20	4	5	0.74	1.06
***D. gallinae*L1 9001**	48	1	-	-	0.00	0.00	0	0	1	1	0.00	0.00	0.00	24	8	2	0.23	1.83
***D. apodis*GO**	40	3	0.14	P	0.01	0.01	4	9	2	2	0.50	2.01	4.51	20	3	4	0.28	0.39
***D. carpathicus*BER7**	40	5	0.70	A	0.01	0.01	4	4	3	4	0.61	1.83	1.83	24	0	1	0.00	0
***D. hirundinis*DhirF**	36	1	-	-	0.00	0.00	0	0	1	1	0.00	0.00	0.00	21	15	4	0.53	4.59

**Table 5.** Mapping isolates in the most supported intraspecific clades. (**a**) Information on clades. (**b**) Percentage of common isolates in mt clade/n clade.

**(a)**		**Ratio external/internal mt branch length**	**Number of haplotypes**	**Number of isolates**	**Number of occurrences (COI haploid, Tpm diploid)**	**Bootstrap (MP gap 5th state)**	**Relative Bremer index (MP gap 5th state)**	**BPP**
COI	*D. gallinae* L1	7.3	3	6	34	100	100	0.99
	*D. gallinae* Lmt1	7.5	3	12	20	99	100	0.9
	*D. gallinae* Lmt2	0.3	7	6	15	61	100	0.69
	*D. gallinae* Lmt3	0.3	12	4	58	23	50	0.95
	*D. carpathicus* Lmt4	4.5	4	4	33	100	100	0.99
	*D. carpathicus* Lmt5	1.0	5	3	6	99	100	0.68
Tpm	*D. gallinae* L1		7	6	70	96	92	0.97
	*D. gallinae* Ln1		3	13	38	100	88	1
	*D. gallinae* Ln2		7	16	81	97	85	1
	*D. gallinae* Ln3		8	4	12	92	94	1
	*D. gallinae* Ln4		11	11	55	81	83	0.96
	*D. carpathicus* Ln5		7	3	58	68	100	0.79
COI	*D. gallinae* L1	7.3	3	6	34	100	100	0.99
	*D. gallinae* Lmt1	7.5	3	12	20	99	100	0.9
	*D. gallinae* Lmt2	0.3	7	6	15	61	100	0.69
	*D. gallinae* Lmt3	0.3	12	4	58	23	50	0.95
	*D. carpathicus* Lmt4	4.5	4	4	33	100	100	0.99
	*D. carpathicus* Lmt5	1.0	5	3	6	99	100	0.68
Tpm	*D. gallinae* L1		7	6	70	96	92	0.97
	*D. gallinae* Ln1		3	13	38	100	88	1
	*D. gallinae* Ln2		7	16	81	97	85	1
	*D. gallinae* Ln3		8	4	12	92	94	1
	*D. gallinae* Ln4		11	11	55	81	83	0.96
	*D. carpathicus* Ln5		7	3	58	68	100	0.79

**(b)**						
	***L1***	***Ln1***	***Ln2***	***Ln3***	***Ln4***	***Ln5***
**L1**	*100/100*	*0/0*	*0/0*	*0/0*	*0/0*	*0/0*
**Lmt1**	*0/0*	*25/23*	*58/44*	*0/0*	*33/36*	*0/0*
**Lmt2**	*0/0*	*100/46*	*50/19*	*17/17*	*50/27*	*0/0*
**Lmt3**	*0/0*	*50/15*	*50/13*	*75/75*	*50/18*	*0/0*
**Lmt4**	*0/0*	*0/0*	*0/0*	*0/0*	*0/0*	*75/100*
**Lmt5**	*0/0*	*0/0*	*0/0*	*0/0*	*0/0*	*0/0*

**Table 6.** Consistency Index (as such and excluding uninformative characters) and Retention Index information for “total evidence” and individual MP analyses in **Step 1** and for MP analyses in **Step 2**.

		**gapmode**	**No of trees**	**Tree length**	**CI**	**CI excluding uninformative characters**	**RI**
multi-gene analyses	6comb	missing	1	1911	0.6787	0.6375	0.8529
	6comb	5^th^ state	1	2685	0.6685	0.6421	0.8623
	COI (1comb1)	-	7	738	0.5068	0.4793	0.7776
	16S (1comb2)	missing	5	296	0.6926	0.6527	0.8551
	16S (1comb2)	5^th^ state	5	352	0.7131	0.6863	0.8758
	5.8S (1comb3)	missing	>1000	48	0.875	0.8235	0.9155
	ITS1_2 (1comb4)	missing	4	283	0.8375	0.784	0.891
	ITS1_2 (1comb4)	5^th^ state	8	376	0.8457	0.7986	0.8854
	TropoINTR (1comb5)	missing	18	492	0.8882	0.8721	0.9608
	TropoINTR (1comb5)	5^th^ state	126	911	0.8804	0.8685	0.9635
	TropoEX (1comb6)	-	1	3	1		1
	EF-1α	-	140	165	0.8848	0.7738	0.8545
multi-isolate analyses	Tropo	missing	352	726	0.8278	0.7845	0.9432
	Tropo	5^th^ state	>1000	1371	0.8228	0.8016	0.9494
	COI	-	868	666	0.536	0.5118	0.8657

## References

[1] Futuyma D Moreno G The Evolution of ecological specialization Ann. Rev. Ecolog. Syst 1988 19 207 233

[2] Desdevises Y Morand S Legendre P Evolution and determinants of host specificity in the genus *Lamellodiscus* (Monogenea) Biol. J. Linn. Soc 2002 77 431 443

[3] Danforth BN Sipes S Fang J Brady SG The history of early bee diversification based on five genes plus morphology Proc. Natl. Acad. Sci. USA 2006 103 15118 15123 17015826 10.1073/pnas.0604033103 PMC1586180

[4] D’Haese CA Were the first springtails semi-aquatic? A phylogenetic approach by means of 28S rDNA and optimization alignment Proc. Royal Soc.:Biol. Sci 2002 269 1143 1151 10.1098/rspb.2002.1981 PMC1691003 12061958

[5] Zhong S Miller SP Dykhuizen DE Dean AM Transcription, Translation, and the Evolution of Specialists and Generalists Mol. Biol. Evol 2009 26 2661 2678 19706726 10.1093/molbev/msp187 PMC2782325

[6] Kaci-Chaouch T Verneau O Desdevises Y Host specificity is linked to intraspecific variability in the genus *Lamellodiscus* (Monogenea) Parasitology 2008 135 607 616 18394221 10.1017/S003118200800437X

[7] Kuris AM Lafferty KD Parasite-host modelling meets reality: Adaptive peaks and their ecological attributes Evolutionary Biology of Host-Parasite Relationships: Theory Meets Reality Poulin R Skorping A Elsevier Amsterdam, The Netherlands 2000 9 26

[8] Moss WW The mite genus *Dermanyssus*: A survey, with description of *Dermanyssus trochilinis*, n. sp., and a revised key to the species (Acari: Mesostigmata: Dermanyssidae) J. Med. Entomol 1978 14 627 640

[9] Radovsky FJ The evolution of parasitism and the distribution of some Dermanyssoid Mites (Mesostigmata) on vertebrate hosts Mites Ecological and Evolutionary Analyses of Life-History Patterns Houck MA Chapman & Hall New York, NY, USA 1994

[10] Roy L Dowling AP Chauve CM Lesna I Sabelis MW Buronfosse T Molecular phylogenetic assessment of host range in five *Dermanyssus* species Exp. Appl. Acarol 2009 48 115 142 19160062 10.1007/s10493-008-9231-1

[11] Roy L Dowling AP Chauve CM Buronfosse T Delimiting species boundaries within *Dermanyssus* Duges, 1834 (Acari: Dermanyssidae) using a total evidence approach Mol. Phylogenet. Evol 2009 50 446 470 19059487 10.1016/j.ympev.2008.11.012

[12] De Lillo E A modified method for Eriophyoid mite extraction (Acari: Eriophyoidea) Internat. J. Acarol 2001 27 67 70

[13] Oliver JH Jr Notes on reproductive behavior in the Dermanyssidae (Acarina Mesostigmata) J. Med. Entomol 1966 3 29 35 5941562 10.1093/jmedent/3.1.29

[14] Hutcheson HJ Oliver JH Jr Spermiogenesis and reproductive biology of *Dermanyssus gallinae* (DeGeer) (Parasitiformes: Dermanyssidae) J. Med. Entomol 1988 25 321 330 3193424 10.1093/jmedent/25.5.321

[15] Edgar R MUSCLE: Multiple sequence alignment with high accuracy and high throughput Nucleic Acids Res 2004 32 1792 1797 15034147 10.1093/nar/gkh340 PMC390337

[16] Galtier N Gouy M Gautier C SEAVIEW and PHYLO_WIN: Two graphic tools for sequence alignment and molecular phylogeny Comput. Appl. Biosci 1996 12 543 548 9021275 10.1093/bioinformatics/12.6.543

[17] Li B Lecointre G Formalizing reliability in the taxonomic congruence approach Zool. Scr 2009 38 101 112

[18] Rambaut A Drummond, Tracer v1.4 2009 Available at: http://tree.bio.ed.ac.uk/software/tracer (Accessed on 15 December 2007).

[19] Birky CW Fuerst P Maruyama T Organelle gene diversity under migration, mutation and drift: Equilibrium expectations, approach to equilibrium, effects of heteroplasmic cells, and comparison to nuclear genes Genetics 1989 121 613 627 2714640 10.1093/genetics/121.3.613 PMC1203645

[20] Rozas J Rozas R DnaSP, DNA sequence polymorphism: An interactive program for estimating population genetics parameters from DNA sequence data Comput. Appl. Biosci 1995 11 621 625 8808578 10.1093/bioinformatics/11.6.621

[21] Excoffier L Laval G Schneider S Arlequin (version 3.0): An integrated software package for population genetics data analysis Evol. Bioinf. Online 2005 1 47 50 PMC2658868 19325852

[22] Black WC Piesman J Phylogeny of hard- and soft-tick taxa (Acari: Ixodida) based on mitochondrial 16S rDNA sequences Proc. Natl. Acad. Sci. USA 1994 91 10034 10038 7937832 10.1073/pnas.91.21.10034 PMC44952

[23] Sanchis A Michelena JM Latorre A Quicke DL Gardenfors U Belshaw R The phylogenetic analysis of variable-length sequence data: Elongation factor-1alpha introns in European populations of the parasitoid wasp genus *Pauesia* (Hymenoptera: Braconidae: Aphidiinae) Mol. Biol. Evol 2001 18 1117 1131 11371599 10.1093/oxfordjournals.molbev.a003882

[24] Kawakita A Sota T Ascher JS Ito M Tanaka H Kato M Evolution and phylogenetic utility of alignment gaps within intron sequences of three nuclear genes in bumble bees (*Bombus*) Mol. Biol. Evol 2003 20 87 92 12519910 10.1093/molbev/msg007

[25] Hedin MC Maddison WP Phylogenetic utility and evidence for multiple copies of elongation factor-1alpha in the spider genus *Habronattus* (Araneae: Salticidae) Mol. Biol. Evol 2001 18 1512 1521 11470842 10.1093/oxfordjournals.molbev.a003937

[26] Nisbet AJ Huntley JF Mackellar A Sparks N McDevitt R A house dust mite allergen homologue from poultry red mite *Dermanyssus gallinae* (De Geer) Parasite Immunol 2006 28 401 405 16879312 10.1111/j.1365-3024.2006.00862.x

[27] De Rojas M Ubeda JM Cutillas C Mora MD Ariza C Guevara D Utility of ITS1–5.8s-ITS2 and 16S mitochondrial DNA sequences for species identification and phylogenetic inference within the *Rhinonyssus coniventris* species complex (Acari: Rhinonyssidae) Parasitol. Res 2007 100 1041 1046 17096140 10.1007/s00436-006-0356-z

[28] De Rojas M Mora MD Ubeda JM Cutillas C Navajas M Guevara DC Phylogenetic relationships in rhinonyssid mites (Acari: Rhinonyssidae) based on ribosomal DNA sequences: Insights for the discrimination of closely related species Parasitol. Res 2002 88 675 681 12107461 10.1007/s00436-002-0647-y

[29] Navajas M Lagnel J Fauvel G De Moraes G Sequence variation of ribosomal internal transcribed spacers (ITS) in commercially important Phytoseiidae mites Exp. Appl. Acarol 1999 23 851 859 10668860 10.1023/a:1006251220052

[30] Brännström S Morrison DA Mattsson JG Chirico J Genetic differences in internal transcribed spacer 1 between *Dermanyssus gallinae* from wild birds and domestic chickens Med. Vet. Entomol 2008 22 152 155 18498615 10.1111/j.1365-2915.2008.00722.x

[31] Hovemann B Richter S Walldorf U Cziepluch C Two genes encode related cytoplasmic elongation factor 1-alpha (EF-1alpha) in *Drosophila melanogaster* with continuous and stage specific expression Nucleic Acids Res 1988 16 3175 3194 3131735 10.1093/nar/16.8.3175 PMC336487

[32] Danforth BN Ji S Elongation factor-1 alpha occurs as two copies in bees: Implications for phylogenetic analysis of EF-1 alpha sequences in insects Mol. Biol. Evol 1998 15 225 235 9501490 10.1093/oxfordjournals.molbev.a025920

[33] Williams ST Knowlton N Weigt LA Jara JA Evidence for three major clades within the snapping shrimp genus *Alpheus* inferred from nuclear and mitochondrial gene sequence data Mol. Phylogenet. Evol 2001 20 375 389 11527465 10.1006/mpev.2001.0976

[34] Goetze E Elongation factor 1-alpha in marine copepods (Calanoida: Eucalanidae): Phylogenetic utility and unique intron structure Mol. Phylogenet. Evol 2006 40 880 886 16725351 10.1016/j.ympev.2006.04.009

[35] Brady SG Danforth BN Recent intron gain in elongation factor-1alpha of colletid bees (Hymenoptera: Colletidae) Mol. Biol. Evol 2004 21 691 696 14739243 10.1093/molbev/msh062

[36] Klompen H Preliminary Assessment of the utility of elongation factor-1alpha in elucidating relationships among basal Mesostigmata Exp. Appl. Acarol 2000 24 805 820 11345317 10.1023/a:1006432017638

[37] Klompen H Lekveishvili M Black WC Phylogeny of parasitiform mites (Acari) based on rRNA Mol. Phylogenet. Evol 2007 43 936 951 17197202 10.1016/j.ympev.2006.10.024

[38] Nichols R Gene trees and species trees are not the same Trends Ecol. Evol 2000 16 358 364 11403868 10.1016/s0169-5347(01)02203-0

[39] McCracken K Sorenson M Is homoplasy or lineage sorting the source of incongruent mtDNA and nuclear gene trees in the stiff-tailed ducks (*Nomonyx-oxyura*)? Syst. Biol 2005 54 35 55 15805009 10.1080/10635150590910249

[40] Moore WS Inferring phylogenies from the mt-DNA variation: Mitochondrial-gene trees *versus* nuclear-gene trees Evolution 1995 49 718 726 10.1111/j.1558-5646.1995.tb02308.x 28565131

[41] Michaux JR Chevret P Filippucci MG Macholan M Phylogeny of the genus *Apodemus* with a special emphasis on the subgenus *Sylvaemus* using the nuclear IRBP gene and two mitochondrial markers: Cytochrome b and 12SrRNA Mol. Phylogenet. Evol 2002 23 123 136 12069545 10.1016/S1055-7903(02)00007-6

[42] Moss WW An Illustrated Key to the Species of the Acarine Genus *Dermanyssus* (Mesostigmata: Laelapoidea: Dermanyssidae) J. Med. Entomol 1968 1 67 84 5644463 10.1093/jmedent/5.1.67

[43] Peterson A Zoonomen Nomenclatural Data, Version 8.07Available at: http://www.zoonomen.net/avtax/frame.html (Accessed on 24 November 2009).

[44] Seehausen O Takimoto G Roy D Jokela J Speciation reversal and biodiversity dynamics with hybridization in changing environments Mol. Ecol 2007 17 30 44 18034800 10.1111/j.1365-294X.2007.03529.x

[45] Whitney KD Ahern JR Campbell LG Hybridization-prone plant families do not generate more invasive species Biol. Invas 2009 11 1205 1215

[46] Peccoud J Ollivier A Plantegenest M Simon JC A continuum of genetic divergence from sympatric host races to species in the pea aphid complex Proc. Natl. Acad. Sci. USA 2009 106 7495 7500 19380742 10.1073/pnas.0811117106 PMC2678636

