# Supplementary material for: Diversity of Phylogenetic Information According to the Locus and the Taxonomic Level: An Example from a Parasitic Mesostigmatid Mite Genus
Source: Int J Mol Sci. 2010 Apr 13;11(4):1704–34. doi: 10.3390/ijms11041704 (PMC2871134; doi:10.3390/ijms11041704)
Supplement: Supplementary file 2 — Appendix 2 (Primer sequences) [file app2.pdf]

Appendix 2. Primer sequences.

Gene	Primer name	Primer sequence 5'-3'	Comments
partial 16S rRNA		as in Roy *et al.* (2009a)	
partial COI		as in Roy *et al.* (2009a and b)	
ITS1-5.8S-ITS2		as in Roy *et al.* (2009a)	
Tropomyosin partial exon n, complete intron n,	TropoF5bis-F	TCGAGCACAGGAACATCACTG	
partial exon n+1	TropoF5bis-R	AGTCTCGGCACGGTCTTCA	
Tropomyosin partial intron n, partial exon n + 1	HET184A9M-F	TAGAGAAGAAGCTAGCATTGT	indel B1 absent
	HET184A9P-F	GAATTGTCTCTAGCATTGTT	indel B1 present
	HET184P2-F	GAGAAGAATTGTCTCTAGCATTGT	indel B1 present
	HET420M-F	CAAAGCACAGCCAGGCTCAC	indel G absent
	HET420P-F	CAAACCGCACAGCCAGGC	indel G present
	HET240M-F	GAGGATGGCGTGAATTCCAAT	indel C1 absent
	HET240P-F	GTGAACCGGAAAATGTGAATTC	indel C1 present
	HET403M-F	TAAGCTAATCGAACCGCACA	indel F absent
	HET403P-F	TAAGCTAATCGGACCATCGA	indel F present
	HET1660CT-F	ACTAGGAAAGACTAGCTGGA	indel ID3bis absent
	HET166AGCT-F	ACTAGAGCTAGGAAAGACTA	indel ID3bis present
Tropomyosin partial exon n,	HET403M-R	TGTGCGGTTCGATTAGCTTA	indel F absent
partial intron n	HET403P-R	TCGATGGTCCGATTAGCTTA	indel F present
	HETDC401a-R	GTTGAGCCTTCACAGCGTGAT	point mutation A/G
	HETDC401b-R	GTTGGGCCTTCACAGCGTGAT	point mutation A/G
partial EF-1alpha	AcEF1-F	CTGTGGAAGTTCGAGACGCC	
	AcEF1-R	CTCGTGGTGCATTTCGACCGACTTC	

Primer pairs : 16S, COI and ITS as in Roy *et al.* (2009a, b); Tropomyosin: TropoF5bis-F + TropoF5bis-R, AcEF1-F+ AcEF1-R.
