# Supplementary material for: Diversity of Phylogenetic Information According to the Locus and the Taxonomic Level: An Example from a Parasitic Mesostigmatid Mite Genus
Source: Int J Mol Sci. 2010 Apr 13;11(4):1704–34. doi: 10.3390/ijms11041704 (PMC2871134; doi:10.3390/ijms11041704)
Supplement: Supplementary file 3 — Appendix 3 (matrix of encoded Indels in Tropomyosin) [file app3.pdf]

Appendix 3. List of discrete characters encoded from indels recorded on Tropomyosin alignments

Appendix 3-1. On the whole dataset, 12 different regions with indels variable intraspecifically have been identified based on all Tropomyosin gene copies obtained from individuals belonging to *D. gallinae* (named using the first letters of alphabet, followed in some cases by a number). Additionnally, insertions/deletions which were not potentially discriminant between populations of *D. gallinae* were named using ID (In/Del) followed by a number.

Character name	Presence of intraspecific variation according to presence/absence of the considered insert	Position of insert (based on alignment ISOL-TRO1)	Character state	Code	Character state species of Dermanyssus concerned	Comments
ID1		37	c	1	*D. carpathicus*	
			absent	0	*all others*	
ID2		106-108	ttc	2	*D. gallinae*	
			gtc	1	*D. apodis*	
			absent	0	*D. carpathicus, D. hirundinis, D. longipes*	
A	x	130-132	gtg	1	*D. gallinae*	
			absent	0	*D. gallinae, D. apodis, D. carpathicus, D. hirundinis, D. longipes*	
ID3	x	170	g	1	*D. longipes (population ENVL08)*	
			absent	0	*D. gallinae, D. apodis, D. carpathicus, D. hirundinis, D. longipes*	
B1	x	179-184	ttgtct	1	*D. gallinae*	
			g	0	*D. gallinae, D. apodis, D. hirundinis, D. longipes*	
			tag	2	*D. carpathicus*	

Appendix 3-1. *Cont.*

Character name	Presence of intraspecific variation according to presence/absence of the considered insert	Position of insert (based on alignment ISOL-TRO1)	Character state	Code	Character state species of Dermanyssus concerned	Comments
B2	x	195-199	ctttg	1	*D. gallinae, D. apodis*	
			ttttt	2	*D. gallinae*	
			cttta	3	*D. gallinae*	
			tttcg	4	*D. carpathicus, D. hirundinis, D. longipes*	
			ttttg	5	*D. gallinae*	
			absent	0	*D. gallinae*	
ID4	x	220-223	aaag	1	*D. hirundinis, D. longipes (+ 1 D. gallinae individual 8018)*	
			aaaa	2	*D. carpathicus*	
			gaag	3	*D. gallinae, D. apodis*	
			absent	0	*D. longipes (population ENVL08)*	
ID5		228-229	tt	1	*D. carpathicus*	
			absent	0	*D. gallinae, D. apodis, D. hirundinis, D. longipes*	
ID6-C1 complex	x	230-254	absent	0	*D. longipes (population ENVL08)*	
			c/tggtttgaaccgaa/ gtttgaatt	2	*D. carpathicus, D. hirundinis, D. longipes*	
			tggtttgaaccgaaaa agtttag	3	*D. apodis*	numerous nucleotide mutations
			tggc/tg/ttgaaccg gaaaat/ag/ttgaa	3	*D. gallinae*	
			tggcgtgaa	1	*D. gallinae*	

Appendix 3-1. *Cont.*

Character name	Presence of intraspecific variation according to presence/absence of the considered insert	Position of insert (based on alignment ISOL-TRO1)	Character state	Code	Character state	species of Dermanyssus concerned	Comments
ID7-C2 complex	x	258-267	a/ctttttaaaa	2	*D. gallinae*		
			atttttttta	2	*D. gallinae*		
			absent	0	*D. gallinae*		
			atgtttaaaa	2	*D. apodis*		
			gttttttaaa	2	*D. longipes (population ENVL08)*		
			gtttttaaa	1	*D. longipes (population ENVL08)*		
			gttttttaaaa	2	*D. hirundinis, D. longipes*		
			gttttaaatt	2	*D. carpathicus*		
ID8	x	273	c	1	*D. carpathicus*		
			absent	0	*D. gallinae, D. apodis, D. carpathicus, D. hirundinis, D. longipes*		
ID9		286-288	cta	2	*D. gallinae, D. apodis*		
			cca	1	*D. gallinae*		
			absent	0	*D. carpathicus, D. hirundinis, D. longipes*		
ID10		297-299	ttc	1	*D. hirundinis*		
			absent	0	*D. gallinae, D. apodis, D. carpathicus, D. longipes*		
D	x	318-322	tagta	1	*D. gallinae, D. apodis, D. carpathicus, D. hirundinis, D. longipes*		
			absent	0	*D. gallinae*		
E	x	347-353	cgctcga	1	*D. gallinae, D. apodis, D. hirundinis, D. longipes*		
			tgctcga	2	*D. carpathicus*		
			absent	0	*D. gallinae*		

Appendix 3-1. *Cont.*

Character name	Presence of intraspecific variation according to presence/absence of the considered insert	Position of insert (based on alignment ISOL-TRO1)	Character state	Code	Character state species of Dermanyssus concerned	Comments
ID11		354-355	aa	1	*D. gallinae, D. carpathicus, D. hirundinis, D. longipes*	
			ga	2	*D. apodis*	
			absent	0	*D. longipes (population ENVL08)*	
ID12		377-380	atac	2	*D. apodis*	
			a	1	*D. gallinae, D. carpathicus*	
			absent	0	*D. hirundinis, D. longipes*	
F	x	398-408	attggacc	1	*D. gallinae, D. apodis*	
			attggact	5	*D. longipes*	
			atcggat	2	*D. carpathicus*	
			attggaccgac	4	*D. hirundinis*	
			attggaccgc	3	*D. longipes*	
			absent	0	*D. gallinae*	
ID13	x	415-416	cc	1	*D. gallinae, D. apodis, D. carpathicus, D. hirundinis, D. longipes*	
			c	2	*D. hirundinis*	
			absent	0	*D. carpathicus*	
G	x	422-425	gtca	1	*D. gallinae, D. apodis, D. carpathicus, D. hirundinis, D. longipes*	
			gcca	2	*D. gallinae*	
			gtcc	3	*D. gallinae*	
			gcct	4	*D. gallinae*	
			absent	0	*D. gallinae*	

Appendix 3-1. *Cont.*

Character name	Presence of intraspecific variation according to presence/absence of the considered insert	Position of insert (based on alignment ISOL-TRO1)	Character state	Code	Character state species of Dermanyssus concerned	Comments
H	x	426-433	ggcggc	1	*D. gallinae*	
			ggcggctc	2	*D. apodis*	
			absent	0	*D. gallinae, D. carpathicus, D. hirundinis, D. longipes*	
ID14	x	445-449	tgaag	1	*D. carpathicus*	
			tgaaa	2	*D. carpathicus*	
			c	3	*D. gallinae (one individual)*	
			absent	0	*D. gallinae, D. apodis, D. hirundinis, D. longipes*	
ID15		453-455	ctg	1	*D. gallinae, D. apodis*	
			absent	0	*D. carpathicus, D. hirundinis, D. longipes*	
ID16		464-467	agct	1	*D. apodis*	
			absent	0	*D. gallinae, D. carpathicus, D. hirundinis, D. longipes*	
ID17		483	g	1	*D. gallinae, D. apodis, D. carpathicus, D. hirundinis, D. longipes*	
			absent	0	*D. hirundinis*	
ID18		506-508	atg	1	*D. gallinae, D. apodis*	
			absent	0	*D. carpathicus, D. hirundinis, D. longipes*	
J	x	564-582	TGAx2	1	*D. gallinae*	TGC instead of TGA in some cases
			TGAx3	2	*D. gallinae*	TGC instead of TGA in some cases
			TGAx4	3	*D. gallinae*	TGC instead of TGA in some cases
			TGAx5	4	*D. gallinae*	TGC instead of TGA in some cases
			TGAx6	5	*D. gallinae*	TGC instead of TGA in some cases

Appendix 3-1. *Cont.*

Character name	Presence of intraspecific variation according to presence/absence of the considered insert	Position of insert (based on alignment ISOL-TRO1)	Character state	Code	Character state	species of Dermanyssus concerned	Comments
			TGAx3 + CGGA	6	*D. apodis*		
			absent	0	*D. carpathicus, D. hirundinis, D. longipes*		
ID19		599-601	tcg	1	*D. gallinae, D. apodis*		
			absent	0	*D. carpathicus, D. hirundinis, D. longipes*		
ID20		636	g	1	*D. gallinae*		
			absent	0	*D. apodis, D. carpathicus, D. hirundinis, D. longipes*		
ID21		639-340	tg	1	*D. gallinae, D. apodis, D. longipes*		
			cg	2	*D. hirundinis*		
			absent	0	*D. carpathicus*		
ID22	x	684	a	1	*D. gallinae*		
			absent	0	*D. gallinae, D. apodis, D. carpathicus, D. hirundinis, D. longipes*		
ID23		688-690	gca	1	*D. gallinae, D. apodis*		
			absent	0	*D. carpathicus, D. hirundinis, D. longipes*		

Appendix 3-2. *Cont.*

**Character n^{o}**	**111111111122222222223333**
**Haplotype n^{o}**	**123456789012345678901234567890123**
Tro_1	010011103201011111110101011411101
Tro_2	010001103201011111011101011311101
Tro_3	010011103201011111011101011311101
Tro_4	010011103201011111111101011411101
Tro_5	011011103201011111111101011411101
Tro_6	011011103201011111111101011411101
Tro_7	010000103201011111011101011311101
Tro_8	010011103201011111111101011311101
Tro_9	010011103201011111111101011311101
Tro_10	010011103201011111111101011311101
Tro_11	010011103201011111111101011311101
Tro_12	010011103201011111111101011311101
Tro_13	010011103201011111111101011311101
Tro_14	010011103201011111111101011311101
Tro_15	010011103201011111111101011311101
Tro_16	010011103201011111111101011211101
Tro_17	010011103201011111111101011311101
Tro_18	010001103201011111111101011311101
Tro_19	010001103201011111111101011311101
Tro_20	010001103201011111111101011311101
Tro_21	010011103201011111110101011311101
Tro_22	011011103201010111111101011311101
Tro_23	010001103201001111112001011211101
Tro_24	011011103201011111111101011411101
Tro_25	010001103201001111112001011211101
Tro_26	010011103201011110101010111311101
Tro_27	011011101201011111111101011511101
Tro_28	020001103211011212111301111610101
Tro_29	020001103211011212111201111610101
Tro_30	100021112200012101111010010000000
Tro_31	100021112200012101111010010000000
Tro_32	100021112200012101111010010000000
Tro_33	100021112200012101111010010000000
Tro_34	100021112200012101111010010000000
Tro_35	100021112200012101111010010000000
Tro_36	100021112200012101111010010000000
Tro_37	100021112200012101111010010000000
Tro_38	100021112200012101111010010000000
Tro_39	100021112200012101111010010000000
Tro_40	100021112200012101101010010000000
Tro_41	000001102220011110113000010000100
Tro_42	000001102220111110121000010000200
Tro_43	000001102220111110121000010000200
Tro_44	000001102220111110111000000000200
Tro_45	000101000120011010111000010000100

Appendix 3-2. Matrix of encoded In/Del.

**Character nº**	**111111111122222222223333**
**Haplotype nº**	**123456789012345678901234567890123**
Tro_46	000101000120011010111000010000100
Tro_47	000101000120011010111000010000100
Tro_48	000101000220011010111000010000100
Tro_49	011011103201011111111101011411101
Tro_50	011011103201011111111101011411101
Tro_51	011011103201011111111101011411101
Tro_52	011011103201001111112001011111101
Tro_53	010001103201001111114001011111101
Tro_54	011011103201011111111101011511101
Tro_55	010000103201011111011101011311101
Tro_56	010001103001011111011101011311101
Tro_57	010000103201001111112001011211101
Tro_58	011011103201001111112001011111101
Tro_59	010011103201001111112001011211111
Tro_60	011011103201001111112031011111101
Tro_D._hirsutus	100001102221011110111300011100101
Tro_T._pyri	000131?000200101100000000000100????
Tro_O._bacoti	100031?0040002111??1?01001000????
