# Supplementary material for: Diversity of Phylogenetic Information According to the Locus and the Taxonomic Level: An Example from a Parasitic Mesostigmatid Mite Genus
Source: Int J Mol Sci. 2010 Apr 13;11(4):1704–34. doi: 10.3390/ijms11041704 (PMC2871134; doi:10.3390/ijms11041704)
Supplement: Supplementary file 4 — Appendix 4 (The different partitioning schemes for the multi-gene analyses and estimation of repetition index) [file app4.pdf]

Appendix 4

Appendix 4-1. Components of partitioning schemes: Elementary datasets (1combn) and of all possible combinations of them (2combn to 6comb).

	COI	16S	5.8S	ITS1_2	Tropo intron n	Tropo exons n & n + 1
1comb1	x					
1comb2		x				
1comb3			x			
1comb4				x		
1comb5					x	
1comb6						x
2comb1	x	x				
2comb2	x		x			
2comb3		x	x			
2comb4	x			x		
2comb5		x		x		
2comb6			x	x		
2comb7	x				x	
2comb8		x			x	
2comb9			x		x	
2comb10				x	x	
2comb11	x					x
2comb12		x				x
2comb13			x			x
2comb14				x		x
2comb15					x	x
3comb1	x	x	x			
3comb2	x	x		x		
3comb3	x	x			x	
3comb4	x	x				x
3comb5	x		x	x		
3comb6	x		x		x	
3comb7	x		x			x
3comb8	x			x	x	
3comb9	x			x		x
3comb10	x				x	x
3comb11		x	x	x		
3comb12		x	x		x	
3comb13		x	x			x
3comb14		x		x	x	
3comb15		x		x		x
3comb16		x			x	x

Appendix 4-1. *Cont.*

	COI	16S	5.8S	ITS1_2	Tropo intron n	Tropo exons n & n + 1
**3comb17**			x	x	x	
**3comb18**			x	x		x
**3comb19**			x		x	x
**3comb20**				x	x	x
**4comb1**	x	x	x	x		
**4comb2**	x	x	x		x	
**4comb3**	x	x	x			x
**4comb4**	x	x		x	x	
**4comb5**	x	x		x		x
**4comb6**	x	x			x	x
**4comb7**	x		x	x	x	
**4comb8**	x		x	x		x
**4comb9**	x		x		x	x
**4comb10**	x			x	x	x
**4comb11**		x	x	x	x	
**4comb12**		x	x	x		x
**4comb13**		x	x		x	x
**4comb14**		x		x	x	x
**4comb15**			x	x	x	x
**5comb1**	x	x	x	x	x	
**5comb2**	x	x	x	x		x
**5comb3**	x	x		x	x	x
**5comb4**	x		x	x	x	x
**5comb5**		x	x	x	x	x
**5comb6**	x	x	x		x	x
**6comb**	x	x	x	x	x	x

Appendix 4-2. Partitioning schemes (pschn).

psch1	1comb1	1comb2	1comb3	1comb4	1comb5	1comb6
psch2	2comb1	1comb3	1comb4	1comb5	1comb6	
psch3	2comb2	1comb2	1comb4	1comb5	1comb6	
psch4	2comb3	1comb2	1comb3	1comb5	1comb6	
psch5	2comb4	1comb2	1comb3	1comb4	1comb6	
psch6	2comb5	1comb2	1comb3	1comb4	1comb5	
psch7	2comb6	1comb1	1comb4	1comb5	1comb6	
psch8	2comb7	1comb1	1comb3	1comb5	1comb6	
psch9	2comb8	1comb1	1comb3	1comb4	1comb6	
psch10	2comb9	1comb1	1comb3	1comb4	1comb5	
psch11	2comb10	1comb1	1comb2	1comb5	1comb6	
psch12	2comb11	1comb1	1comb2	1comb4	1comb6	
psch13	2comb12	1comb1	1comb2	1comb4	1comb5	
psch14	2comb13	1comb1	1comb2	1comb3	1comb6	

Appendix 4-2. *Cont.*

psch15	2comb14	1comb1	1comb2	1comb3	1comb5	
psch16	2comb15	1comb1	1comb2	1comb3	1comb4	
psch17	2comb10	2comb15	1comb1	1comb2		
psch18	2comb11	2comb14	1comb1	1comb2		
psch19	2comb12	2comb13	1comb1	1comb2		
psch20	2comb7	2comb15	1comb1	1comb3		
psch21	2comb8	2comb14	1comb1	1comb3		
psch22	2comb9	2comb13	1comb1	1comb3		
psch23	2comb6	2comb15	1comb1	1comb4		
psch24	2comb8	2comb12	1comb1	1comb4		
psch25	2comb9	2comb11	1comb1	1comb4		
psch26	2comb6	2comb14	1comb1	1comb5		
psch27	2comb7	2comb12	1comb1	1comb5		
psch28	2comb9	2comb10	1comb1	1comb5		
psch29	2comb6	2comb13	1comb1	1comb6		
psch30	2comb7	2comb11	1comb1	1comb6		
psch31	2comb8	2comb10	1comb1	1comb6		
psch32	2comb3	2comb15	1comb2	1comb3		
psch33	2comb4	2comb14	1comb2	1comb3		
psch34	2comb5	2comb13	1comb2	1comb3		
psch35	2comb2	2comb15	1comb2	1comb4		
psch36	2comb4	2comb12	1comb2	1comb4		
psch37	2comb5	2comb11	1comb2	1comb4		
psch38	2comb2	2comb14	1comb2	1comb5		
psch39	2comb3	2comb12	1comb2	1comb5		
psch40	2comb5	2comb10	1comb2	1comb5		
psch41	2comb2	2comb13	1comb2	1comb6		
psch42	2comb3	2comb11	1comb2	1comb6		
psch43	2comb4	2comb10	1comb2	1comb6		
psch44	2comb1	2comb15	1comb3	1comb4		
psch45	2comb4	2comb9	1comb3	1comb4		
psch46	2comb5	2comb8	1comb3	1comb4		
psch47	2comb1	2comb14	1comb3	1comb5		
psch48	2comb3	2comb9	1comb3	1comb5		
psch49	2comb5	2comb7	1comb3	1comb5		
psch50	2comb1	2comb13	1comb3	1comb6		
psch51	2comb3	2comb8	1comb3	1comb6		
psch52	2comb4	2comb7	1comb3	1comb6		
psch53	2comb1	2comb12	1comb4	1comb5		
psch54	2comb2	2comb9	1comb4	1comb5		
psch55	2comb5	2comb6	1comb4	1comb5		
psch56	2comb1	2comb11	1comb4	1comb6		
psch57	2comb2	2comb8	1comb4	1comb6		
psch58	2comb4	2comb6	1comb4	1comb6		
psch59	2comb1	2comb10	1comb5	1comb6		
psch60	2comb2	2comb7	1comb5	1comb6		
psch61	2comb3	2comb6	1comb5	1comb6		
psch62	2comb1	2comb10	2comb15			
psch63	2comb1	2comb11	2comb14			
psch64	2comb1	2comb12	2comb13			

Appendix 4-2. *Cont.*

psch65	2comb2	2comb7	2comb15			
psch66	2comb2	2comb8	2comb14			
psch67	2comb2	2comb9	2comb13			
psch68	2comb3	2comb6	2comb15			
psch69	2comb3	2comb8	2comb12			
psch70	2comb3	2comb9	2comb11			
psch71	2comb4	2comb6	2comb14			
psch72	2comb4	2comb7	2comb12			
psch73	2comb4	2comb9	2comb10			
psch74	2comb5	2comb6	2comb13			
psch75	2comb5	2comb7	2comb11			
psch76	2comb5	2comb8	2comb10			
psch77	3comb1	1comb4	1comb5	1comb6		
psch78	3comb2	1comb3	1comb5	1comb6		
psch79	3comb3	1comb3	1comb4	1comb6		
psch80	3comb4	1comb3	1comb4	1comb5		
psch81	3comb5	1comb2	1comb5	1comb6		
psch82	3comb6	1comb2	1comb4	1comb6		
psch83	3comb7	1comb2	1comb4	1comb5		
psch84	3comb8	1comb2	1comb3	1comb6		
psch85	3comb9	1comb2	1comb3	1comb5		
psch86	3comb10	1comb2	1comb3	1comb4		
psch87	3comb11	1comb1	1comb5	1comb6		
psch88	3comb12	1comb1	1comb4	1comb6		
psch89	3comb13	1comb1	1comb4	1comb5		
psch90	3comb14	1comb1	1comb3	1comb6		
psch91	3comb15	1comb1	1comb3	1comb5		
psch92	3comb16	1comb1	1comb3	1comb4		
psch93	3comb17	1comb1	1comb2	1comb6		
psch94	3comb18	1comb1	1comb2	1comb5		
psch95	3comb19	1comb1	1comb2	1comb4		
psch96	3comb20	1comb1	1comb2	1comb3		
psch97	3comb1	2comb13	1comb6			
psch98	3comb1	2comb14	1comb5			
psch99	3comb1	2comb15	1comb4			
psch100	3comb2	2comb11	1comb6			
psch101	3comb2	2comb12	1comb5			
psch102	3comb2	2comb15	1comb3			
psch103	3comb3	2comb10	1comb6			
psch104	3comb3	2comb12	1comb4			
psch105	3comb3	2comb14	1comb3			
psch106	3comb4	2comb10	1comb5			
psch107	3comb4	2comb11	1comb4			
psch108	3comb4	2comb13	1comb3			
psch109	3comb5	2comb8	1comb6			
psch110	3comb5	2comb9	1comb5			
psch111	3comb5	2comb15	1comb2			
psch112	3comb6	2comb7	1comb6			
psch113	3comb6	2comb9	1comb4			
psch114	3comb6	2comb14	1comb2			

Appendix 4-2. *Cont.*

psch115	3comb7	2comb7	1comb5			
psch116	3comb7	2comb8	1comb4			
psch117	3comb7	2comb13	1comb2			
psch118	3comb8	2comb6	1comb6			
psch119	3comb8	2comb9	1comb3			
psch120	3comb8	2comb12	1comb2			
psch121	3comb9	2comb6	1comb5			
psch122	3comb9	2comb8	1comb3			
psch123	3comb9	2comb11	1comb2			
psch124	3comb10	2comb6	1comb4			
psch125	3comb10	2comb7	1comb3			
psch126	3comb10	2comb10	1comb2			
psch127	3comb11	2comb4	1comb6			
psch128	3comb11	2comb5	1comb5			
psch129	3comb11	2comb15	1comb1			
psch130	3comb12	2comb3	1comb6			
psch131	3comb12	2comb5	1comb4			
psch132	3comb12	2comb14	1comb1			
psch133	3comb13	2comb3	1comb5			
psch134	3comb13	2comb4	1comb4			
psch135	3comb13	2comb13	1comb1			
psch136	3comb14	2comb2	1comb6			
psch137	3comb14	2comb5	1comb3			
psch138	3comb14	2comb12	1comb1			
psch139	3comb15	2comb2	1comb5			
psch140	3comb15	2comb4	1comb3			
psch141	3comb15	2comb11	1comb1			
psch142	3comb16	2comb2	1comb4			
psch143	3comb16	2comb3	1comb3			
psch144	3comb16	2comb10	1comb1			
psch145	3comb17	2comb1	1comb6			
psch146	3comb17	2comb5	1comb2			
psch147	3comb17	2comb9	1comb1			
psch148	3comb18	2comb1	1comb5			
psch149	3comb18	2comb4	1comb2			
psch150	3comb18	2comb8	1comb1			
psch151	3comb19	2comb1	1comb4			
psch152	3comb19	2comb3	1comb2			
psch153	3comb19	2comb7	1comb1			
psch154	3comb20	2comb1	1comb3			
psch155	3comb20	2comb2	1comb2			
psch156	3comb20	2comb6	1comb1			
psch157	3comb1	3comb20				
psch158	3comb2	3comb19				
psch159	3comb3	3comb18				
psch160	3comb4	3comb17				
psch161	3comb5	3comb16				
psch162	3comb6	3comb15				
psch163	3comb7	3comb14				
psch164	3comb8	3comb13				

Appendix 4-2. *Cont.*

psch165	3comb9	3comb12				
psch166	3comb10	3comb11				
psch167	4comb1	1comb1	1comb2			
psch168	4comb2	1comb1	1comb3			
psch169	4comb3	1comb1	1comb4			
psch170	4comb4	1comb1	1comb5			
psch171	4comb5	1comb1	1comb6			
psch172	4comb6	1comb2	1comb3			
psch173	4comb7	1comb2	1comb4			
psch174	4comb8	1comb2	1comb5			
psch175	4comb9	1comb2	1comb6			
psch176	4comb10	1comb3	1comb4			
psch177	4comb11	1comb3	1comb5			
psch178	4comb12	1comb3	1comb6			
psch179	4comb13	1comb4	1comb5			
psch180	4comb14	1comb4	1comb6			
psch181	4comb15	1comb5	1comb6			
psch182	4comb1	2comb1				
psch183	4comb2	2comb2				
psch184	4comb3	2comb3				
psch185	4comb4	2comb4				
psch186	4comb5	2comb5				
psch187	4comb6	2comb6				
psch188	4comb7	2comb7				
psch189	4comb8	2comb8				
psch190	4comb9	2comb9				
psch191	4comb10	2comb10				
psch192	4comb11	2comb11				
psch193	4comb12	2comb12				
psch194	4comb13	2comb13				
psch195	4comb14	2comb14				
psch196	4comb15	2comb15				
psch197	5comb1	1comb1				
psch198	5comb2	1comb2				
psch199	5comb3	1comb3				
psch200	5comb4	1comb4				
psch201	5comb5	1comb5				
psch202	5comb6	1comb6				
psch203	6comb					
