# Supplementary material for: Diversity of Phylogenetic Information According to the Locus and the Taxonomic Level: An Example from a Parasitic Mesostigmatid Mite Genus
Source: Int J Mol Sci. 2010 Apr 13;11(4):1704–34. doi: 10.3390/ijms11041704 (PMC2871134; doi:10.3390/ijms11041704)

Appendix 5

The different topologies obtained at the specific level within *Dermanyssus* in multi-gene analyses and in multi-isolate analyses.

A triangle followed by *D. gallinae* + *D. gallinae* L1 represents a *D. gallinae* clade from within which the clade of L1 isolates is branching.

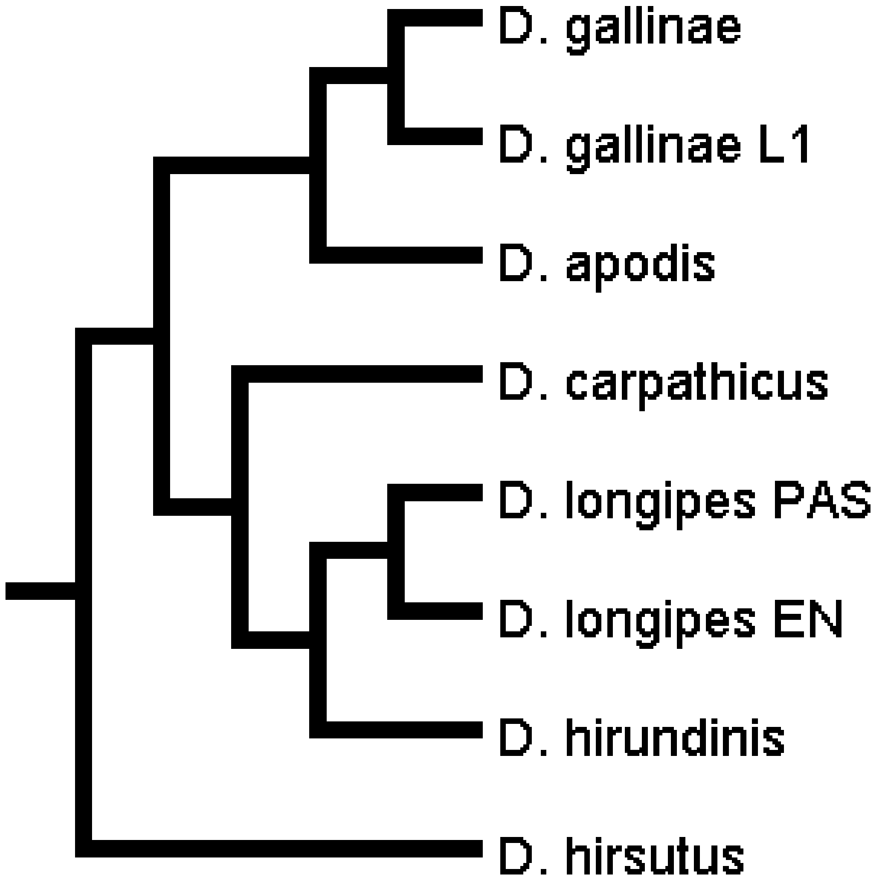

D. gallinae
D. gallinae L1
D. apodis
D. carpathicus
D. longipes PAS
D. longipes EN
D. hirundinis
D. hirsutus

Top2

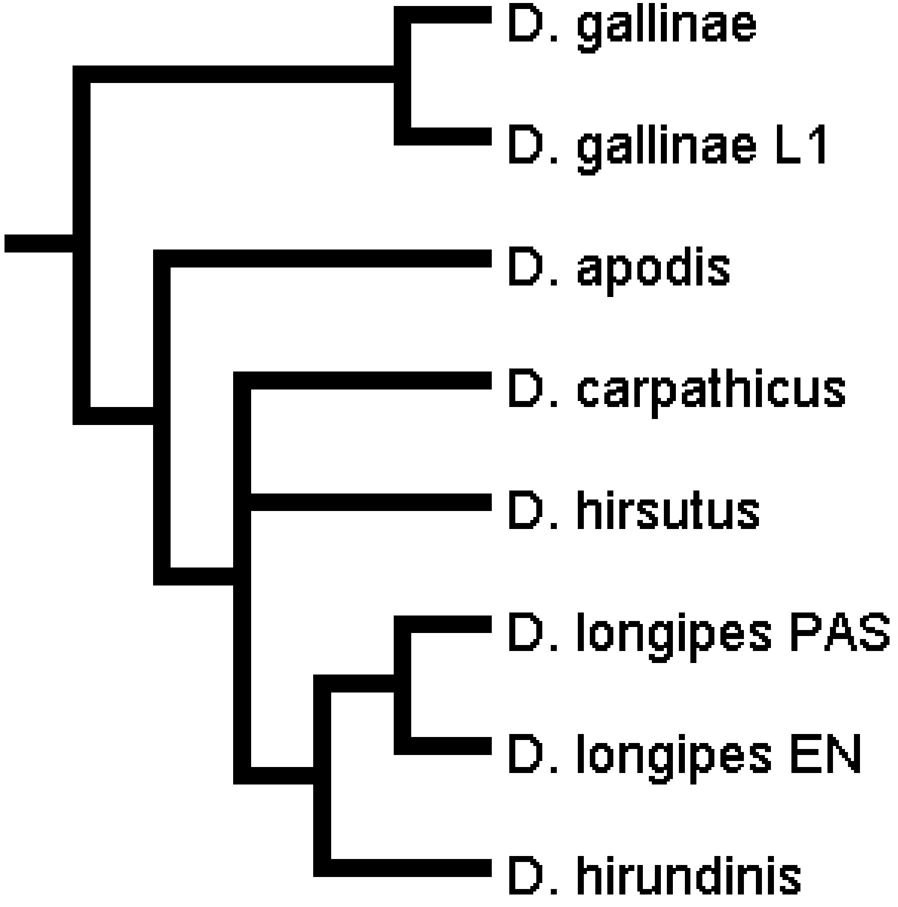
D. gallinae
D. gallinae L1
D. apodis
D. carpathicus
D. hirsutus
D. longipes PAS
D. longipes EN
D. hirundinis

Top5

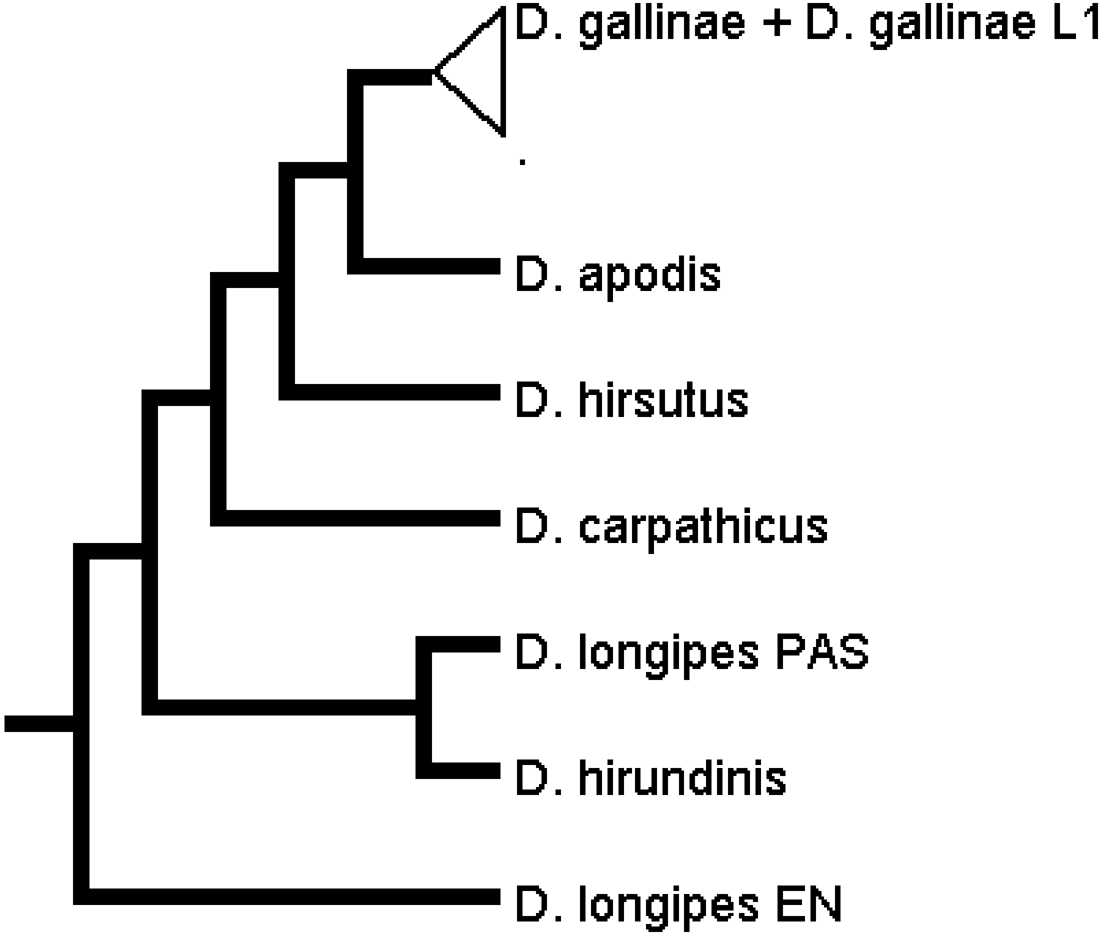

D. gallinae + D. gallinae L1
D. apodis
D. hirsutus
D. carpathicus
D. longipes PAS
D. hirundinis
D. longipes EN

Top6

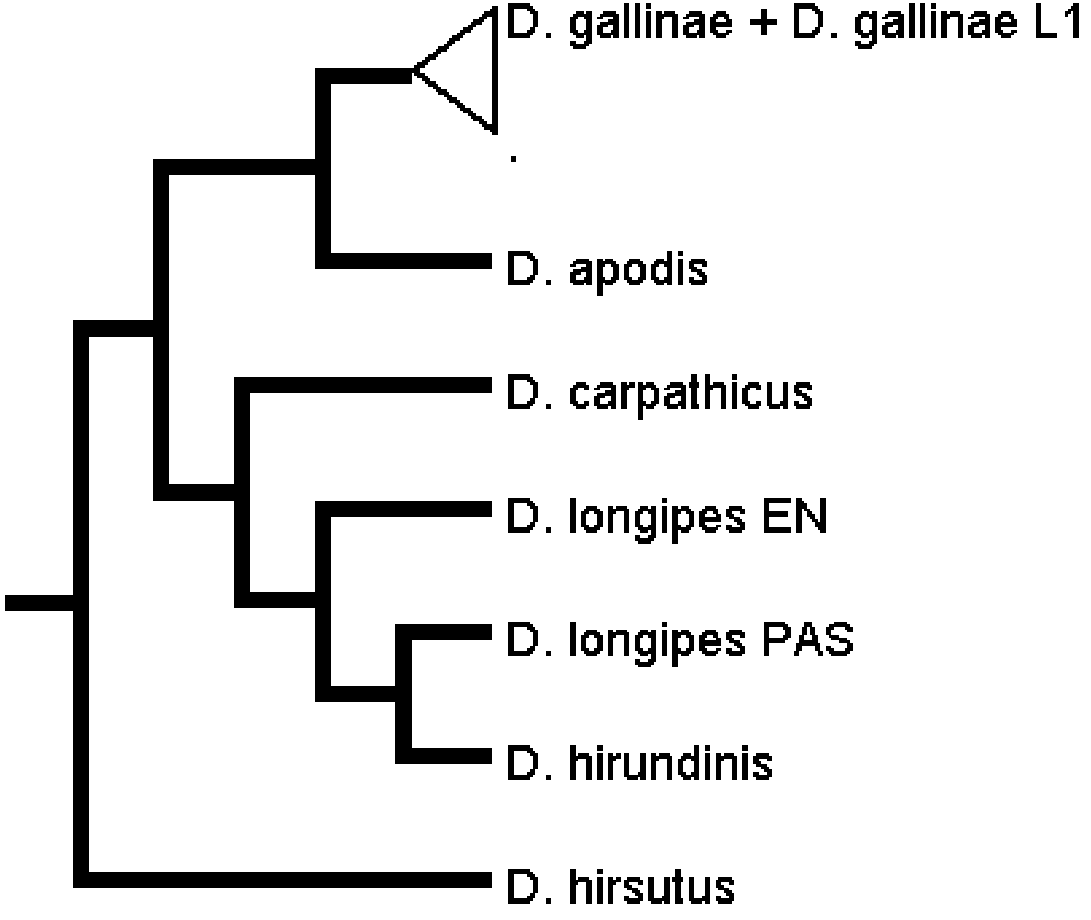
D. gallinae + D. gallinae L1
D. apodis
D. carpathicus
D. longipes EN
D. longipes PAS
D. hirundinis
D. hirsutus

Top7

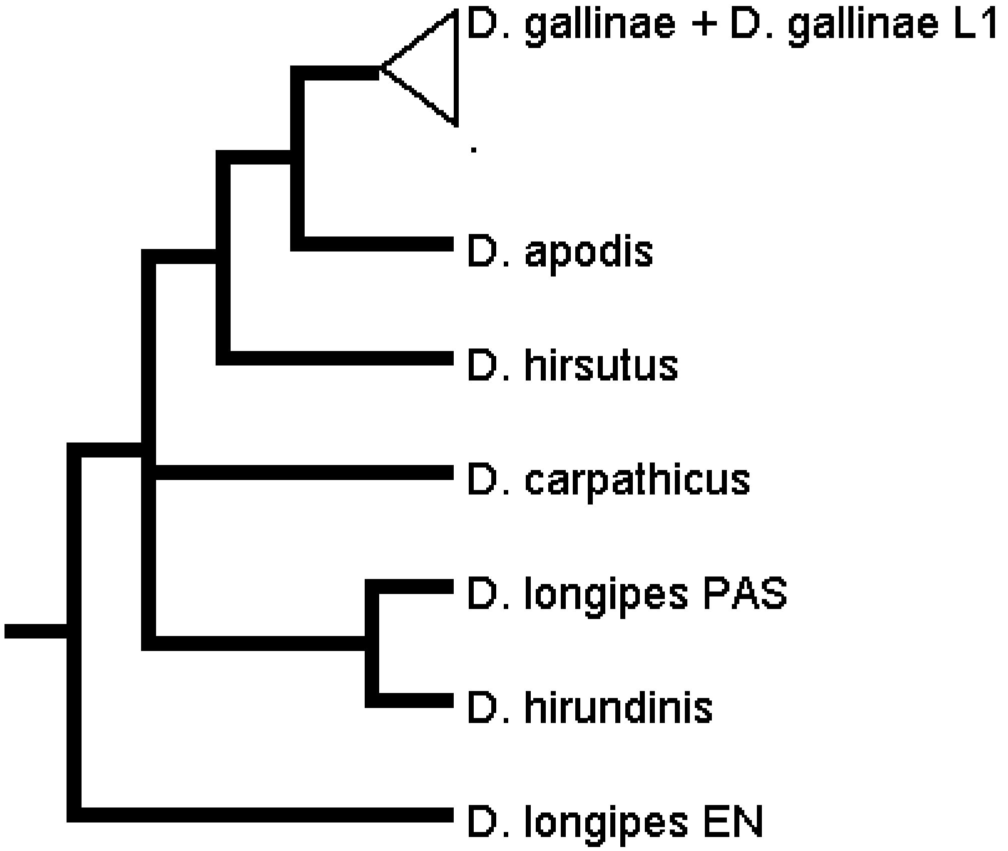
D. gallinae + D. gallinae L1
D. apodis
D. hirsutus
D. carpathicus
D. longipes PAS
D. hirundinis
D. longipes EN

Top8

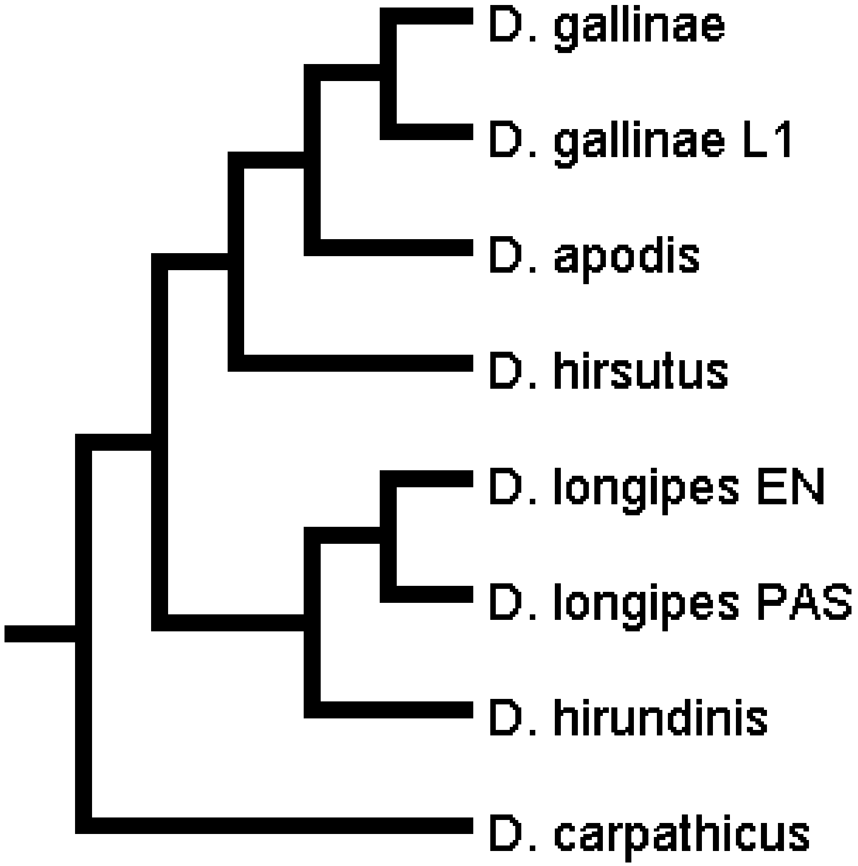
D. gallinae
D. gallinae L1
D. apodis
D. hirsutus
D. longipes EN
D. longipes PAS
D. hirundinis
D. carpathicus

Top9

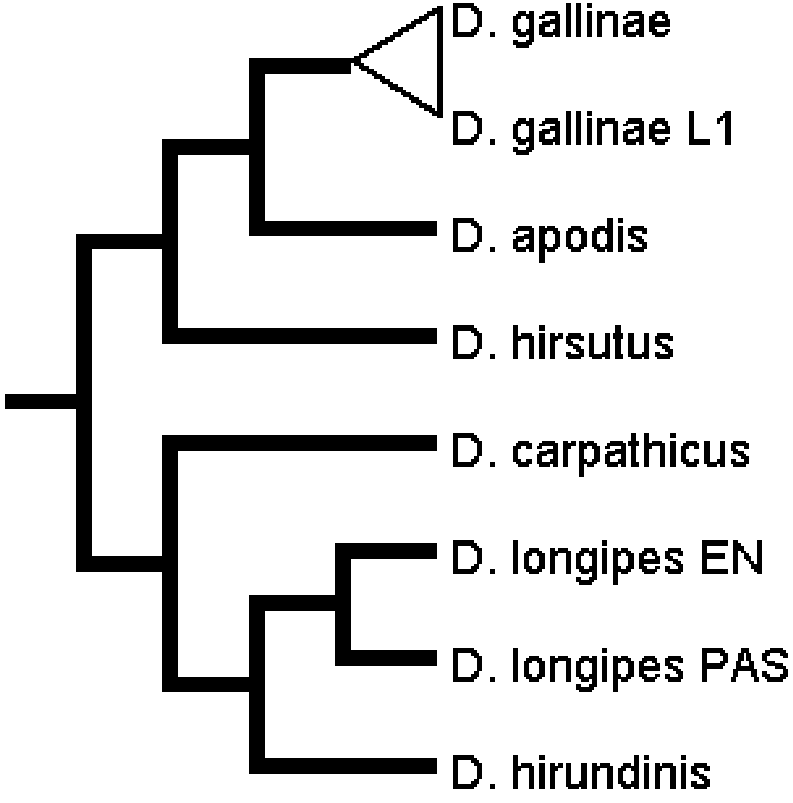

D. gallinae
D. gallinae L1
D. apodis
D. hirsutus
D. carpathicus
D. longipes EN
D. longipes PAS
D. hirundinis

Top10

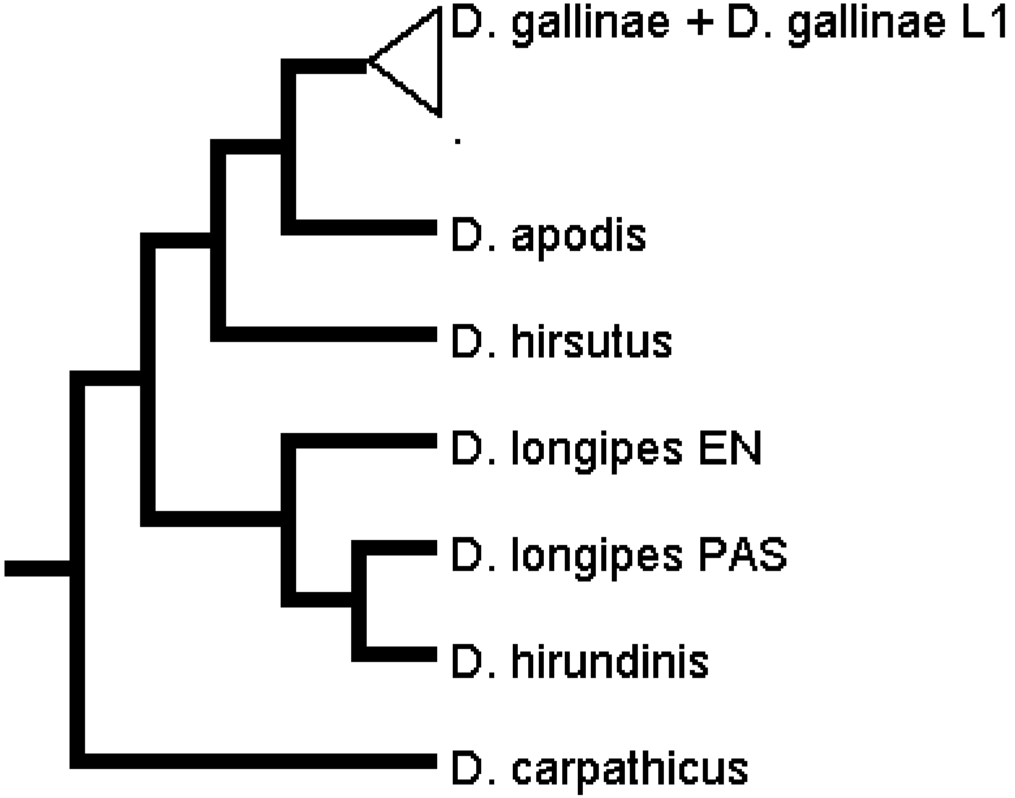
D. gallinae + D. gallinae L1
.
D. apodis
D. hirsutus
D. longipes EN
D. longipes PAS
D. hirundinis
D. carpathicus

Top11

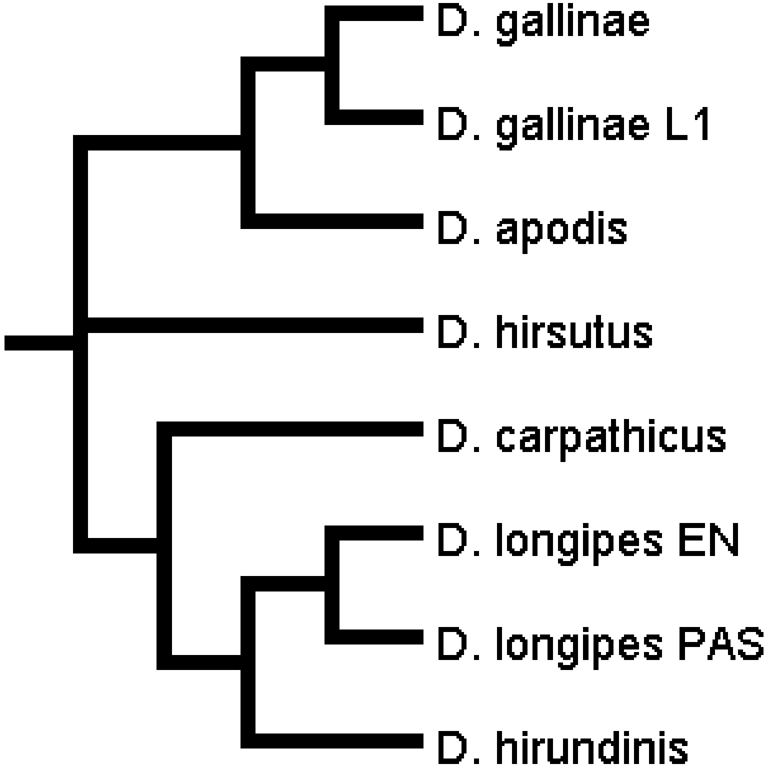

D. gallinae
D. gallinae L1
D. apodis
D. hirsutus
D. carpathicus
D. longipes EN
D. longipes PAS
D. hirundinis

Top12

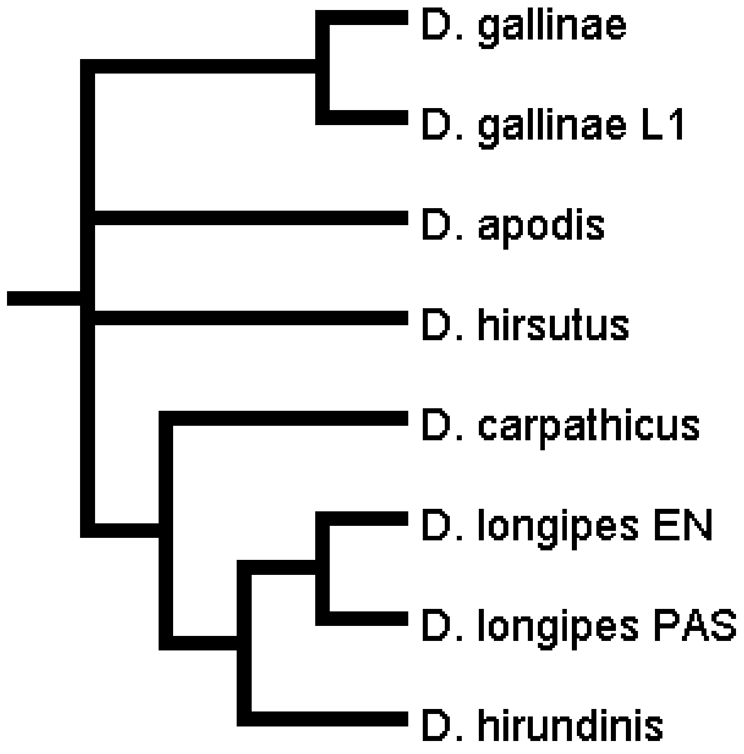
D. gallinae
D. gallinae L1
D. apodis
D. hirsutus
D. carpathicus
D. longipes EN
D. longipes PAS
D. hirundinis

Top13

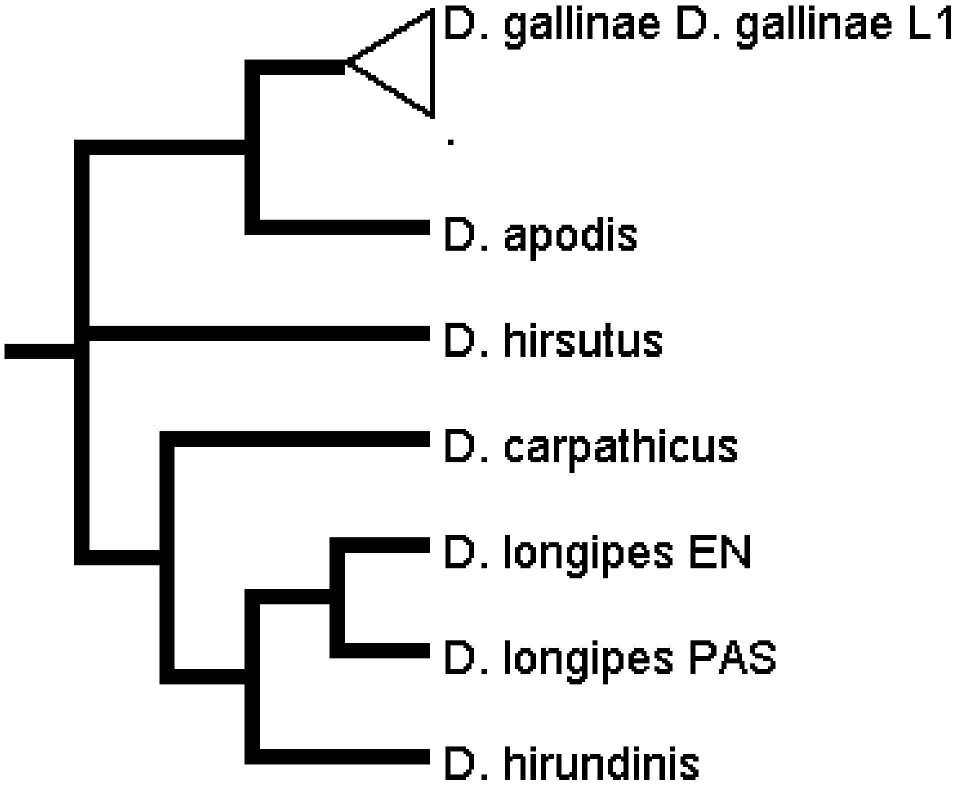
D. gallinae D. gallinae L1
D. apodis
D. hirsutus
D. carpathicus
D. longipes EN
D. longipes PAS
D. hirundinis

Top15

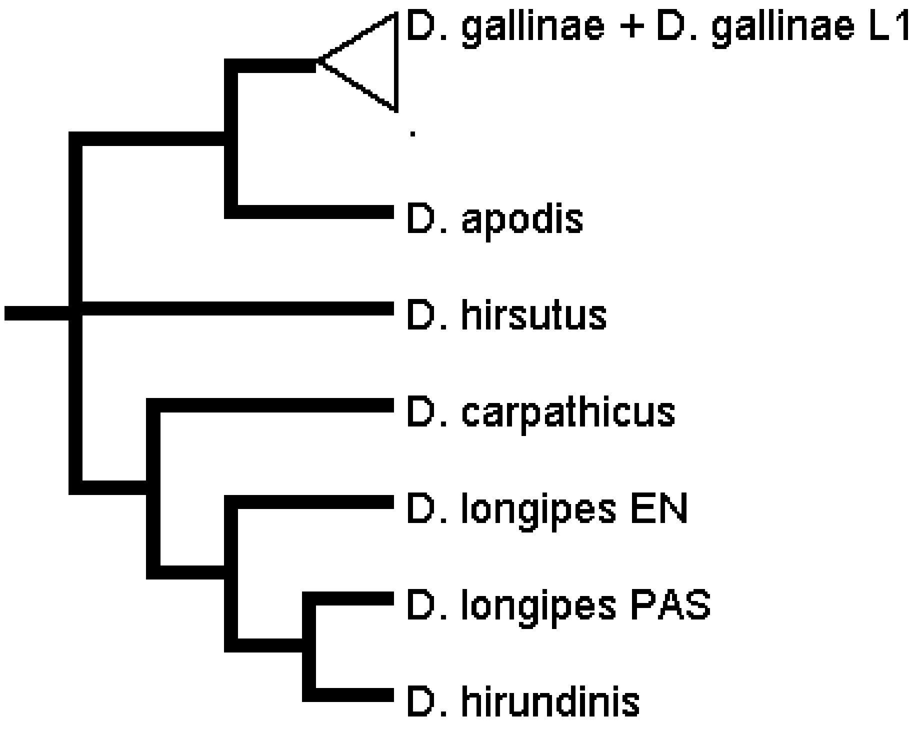
D. gallinae + D. gallinae L1
D. apodis
D. hirsutus
D. carpathicus
D. longipes EN
D. longipes PAS
D. hirundinis

Top16

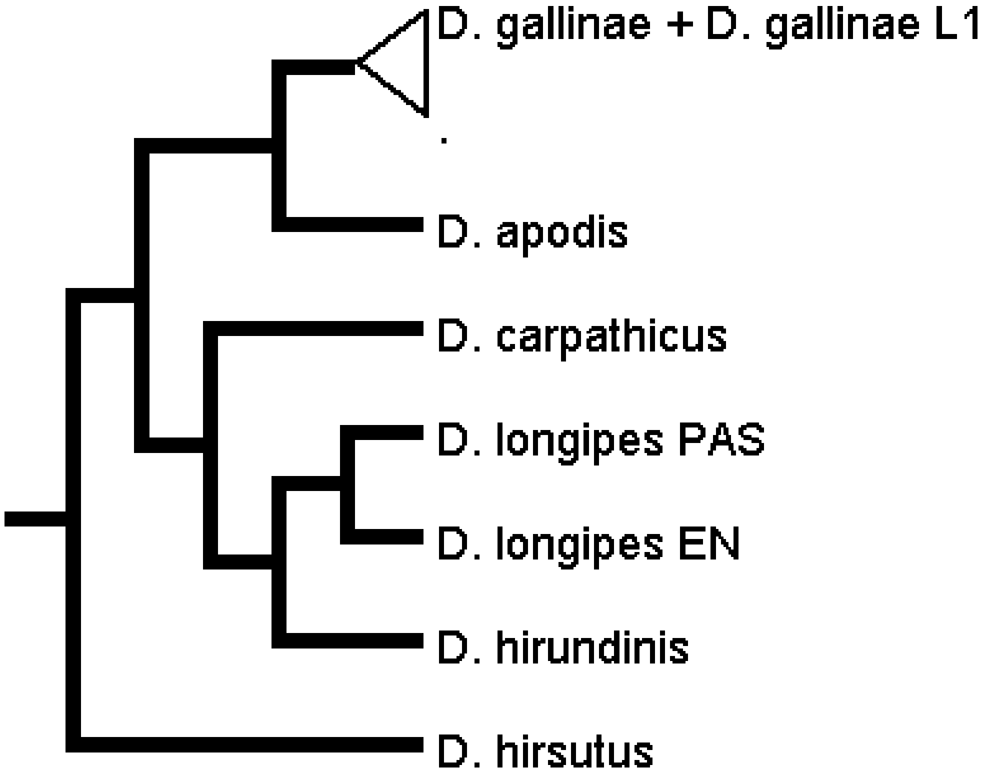
D. gallinae + D. gallinae L1
.
D. apodis
D. carpathicus
D. longipes PAS
D. longipes EN
D. hirundinis
D. hirsutus

Top17

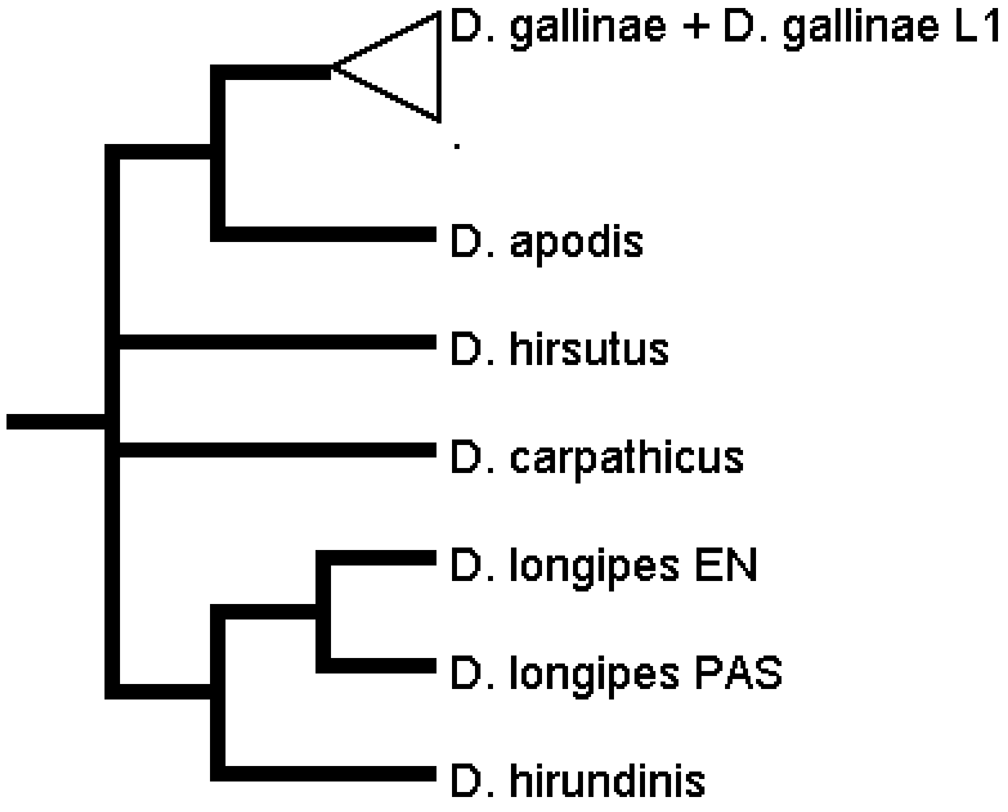
D. gallinae + D. gallinae L1
D. apodis
D. hirsutus
D. carpathicus
D. longipes EN
D. longipes PAS
D. hirundinis

Top18

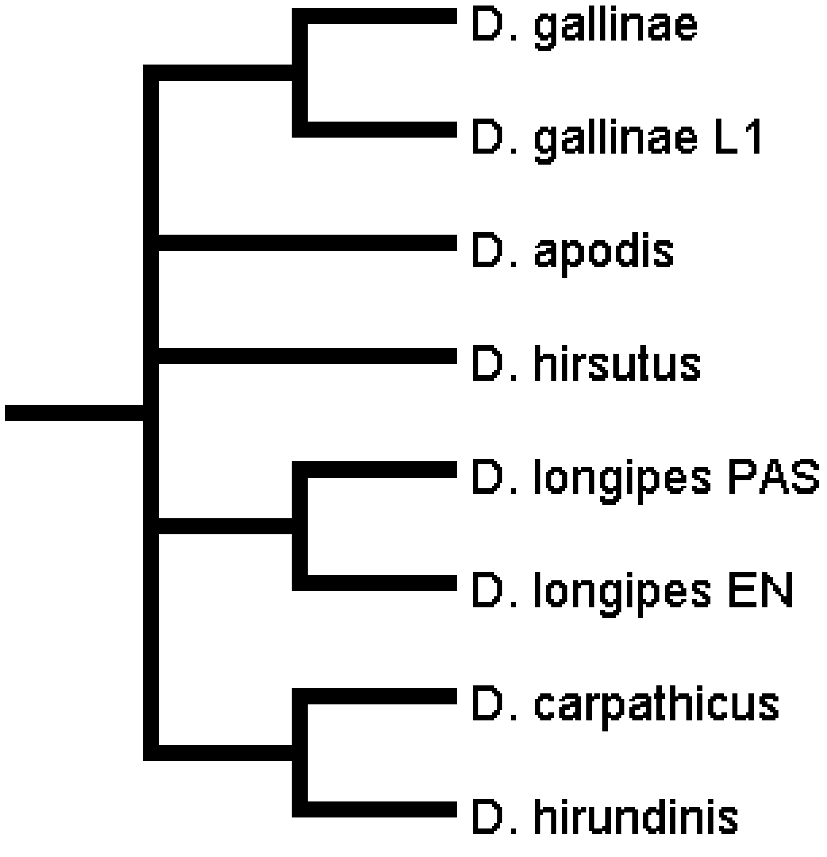

D. gallinae
D. gallinae L1
D. apodis
D. hirsutus
D. longipes PAS
D. longipes EN
D. carpathicus
D. hirundinis

Top19

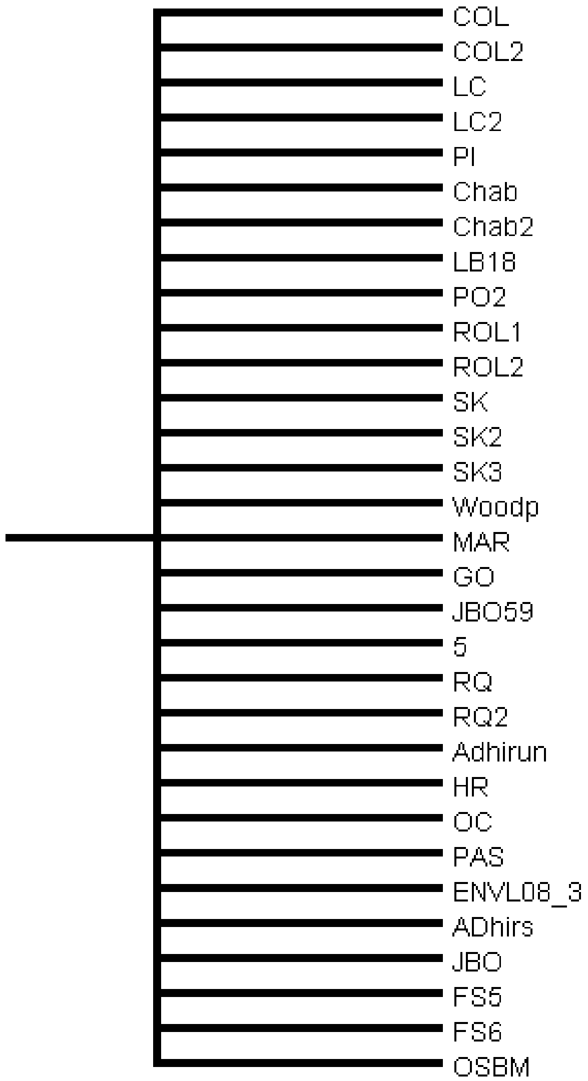
COL
COL2
LC
LC2
PI
Chab
Chab2
LB18
PO2
ROL1
ROL2
SK
SK2
SK3
Woodp
MAR
GO
JBO59
5
RQ
RQ2
Adhirun
HR
OC
PAS
ENVL08_3
ADhirs
JBO
FS5
FS6
OSBM

Top20

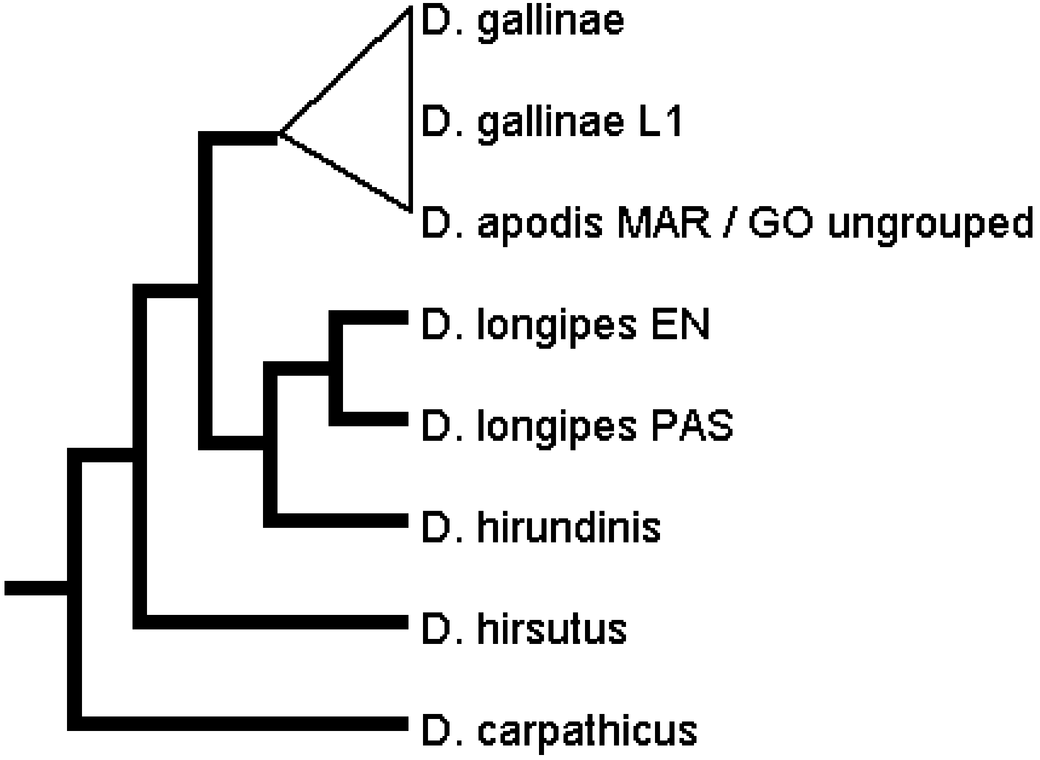
D. gallinae
D. gallinae L1
D. apodis MAR / GO ungrouped
D. longipes EN
D. longipes PAS
D. hirundinis
D. hirsutus
D. carpathicus

Top21

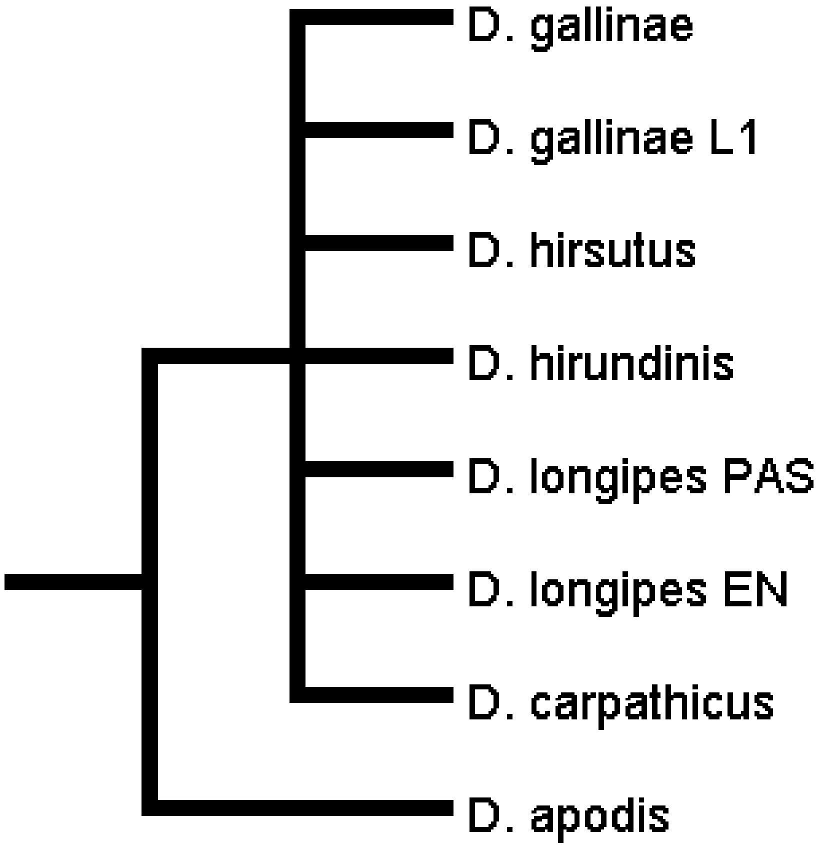
D. gallinae
D. gallinae L1
D. hirsutus
D. hirundinis
D. longipes PAS
D. longipes EN
D. carpathicus
D. apodis

Top22

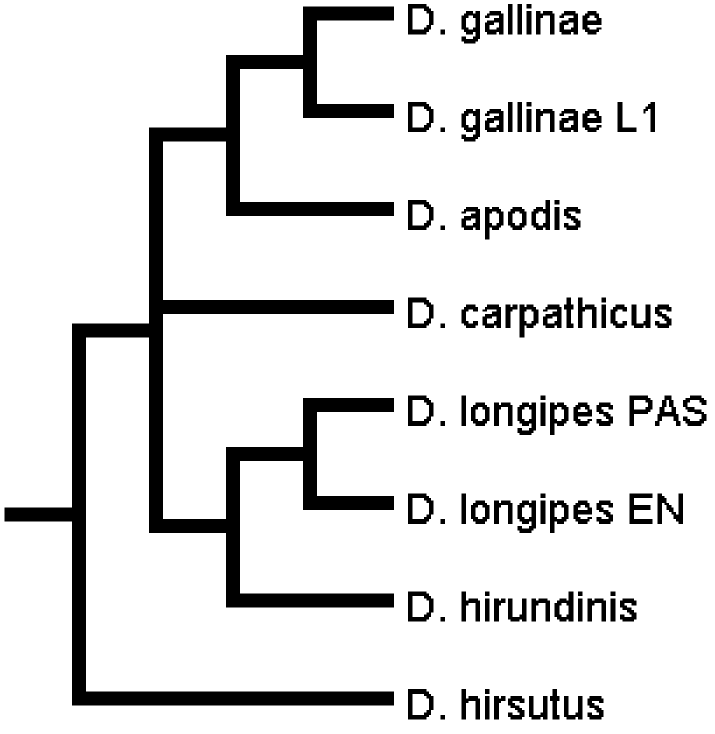
D. gallinae
D. gallinae L1
D. apodis
D. carpathicus
D. longipes PAS
D. longipes EN
D. hirundinis
D. hirsutus

Top23

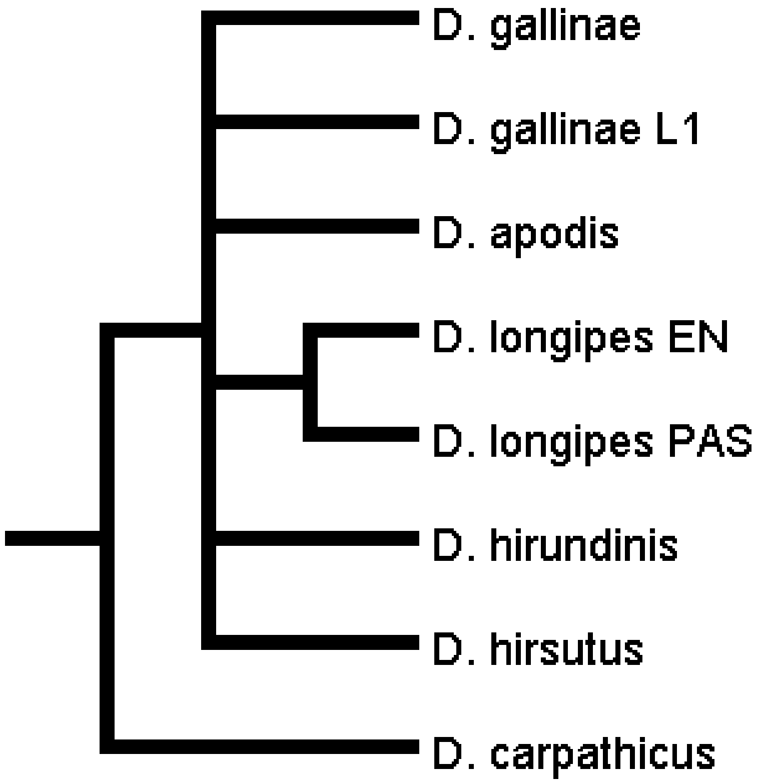
D. gallinae
D. gallinae L1
D. apodis
D. longipes EN
D. longipes PAS
D. hirundinis
D. hirsutus
D. carpathicus

Top24

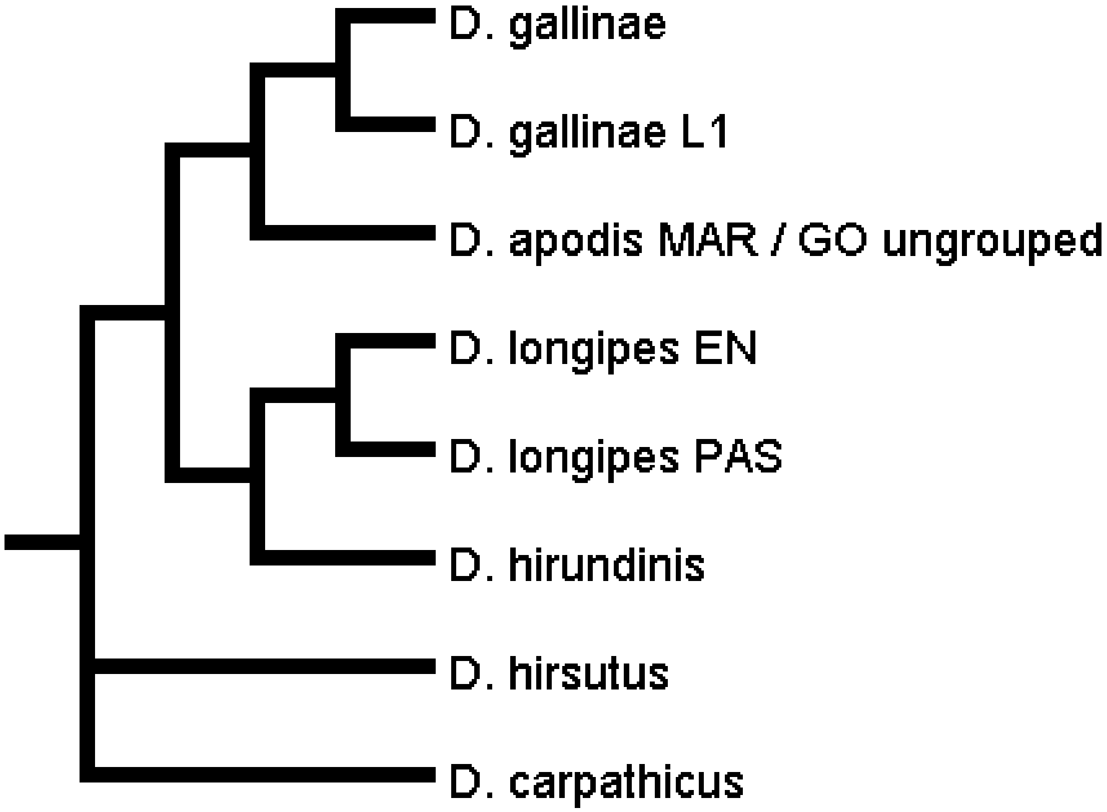
D. gallinae
D. gallinae L1
D. apodis MAR / GO ungrouped
D. longipes EN
D. longipes PAS
D. hirundinis
D. hirsutus
D. carpathicus

Top25

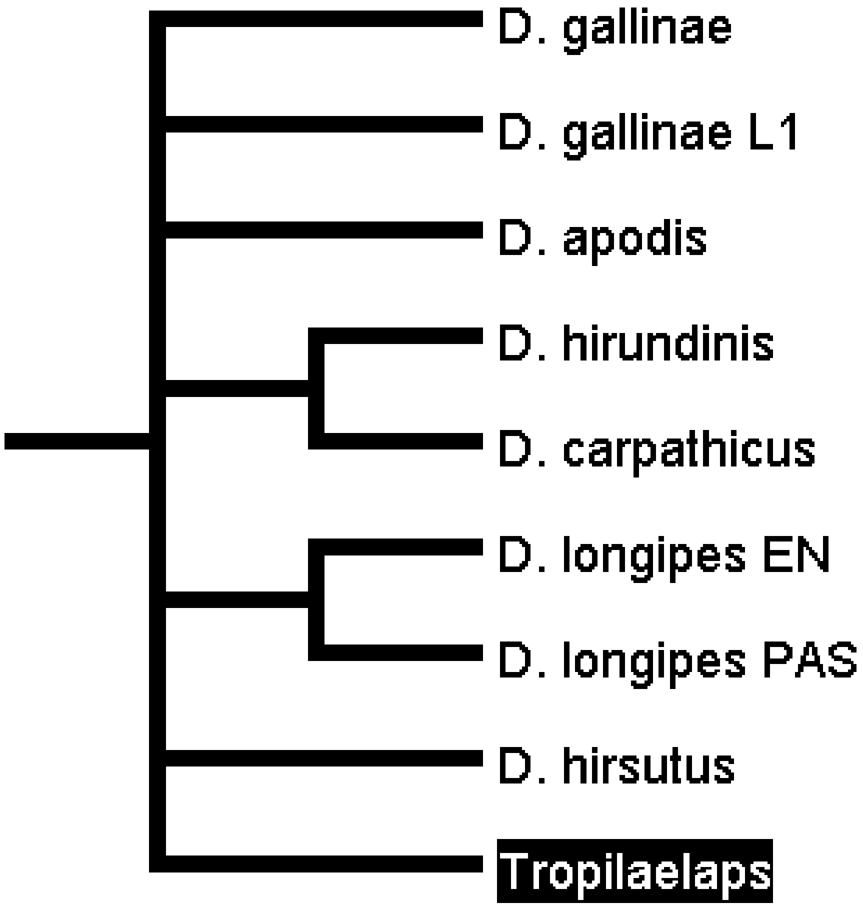
D. gallinae
D. gallinae L1
D. apodis
D. hirundinis
D. carpathicus
D. longipes EN
D. longipes PAS
D. hirsutus
Tropilaelaps

Top26

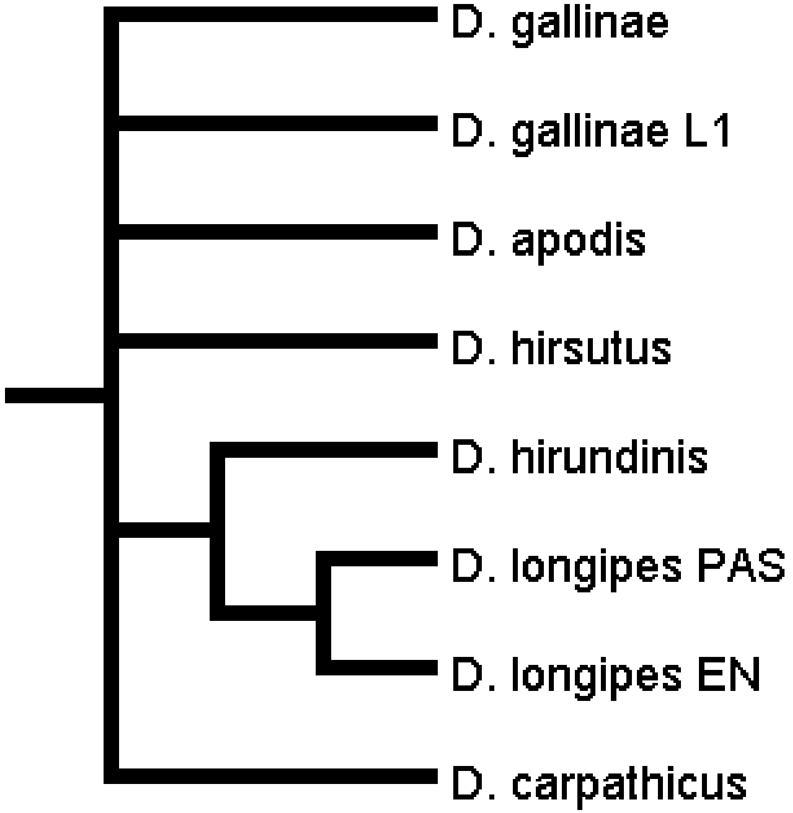
D. gallinae
D. gallinae L1
D. apodis
D. hirsutus
D. hirundinis
D. longipes PAS
D. longipes EN
D. carpathicus

Top27

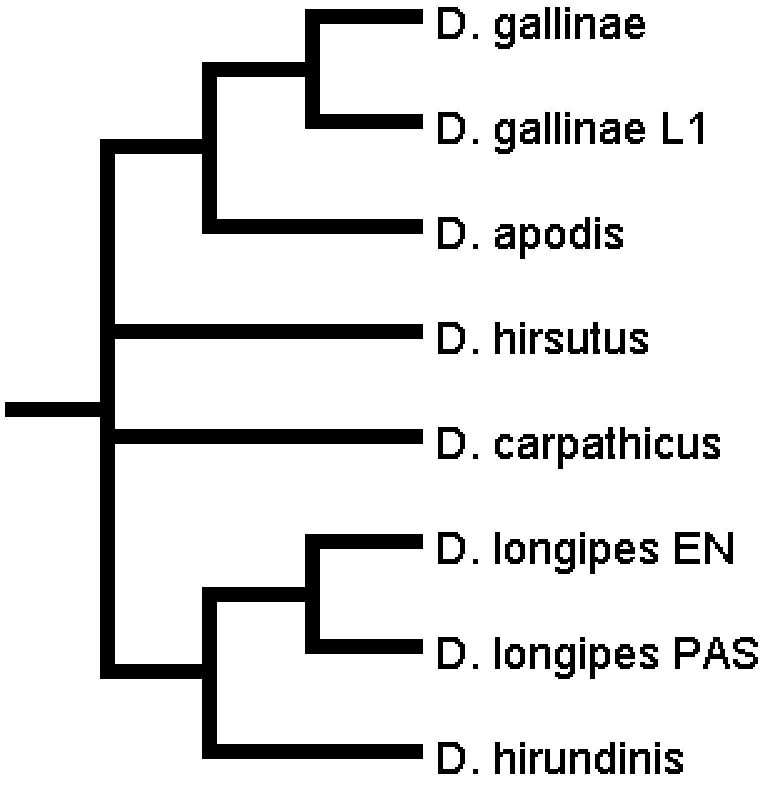
D. gallinae
D. gallinae L1
D. apodis
D. hirsutus
D. carpathicus
D. longipes EN
D. longipes PAS
D. hirundinis

Top28

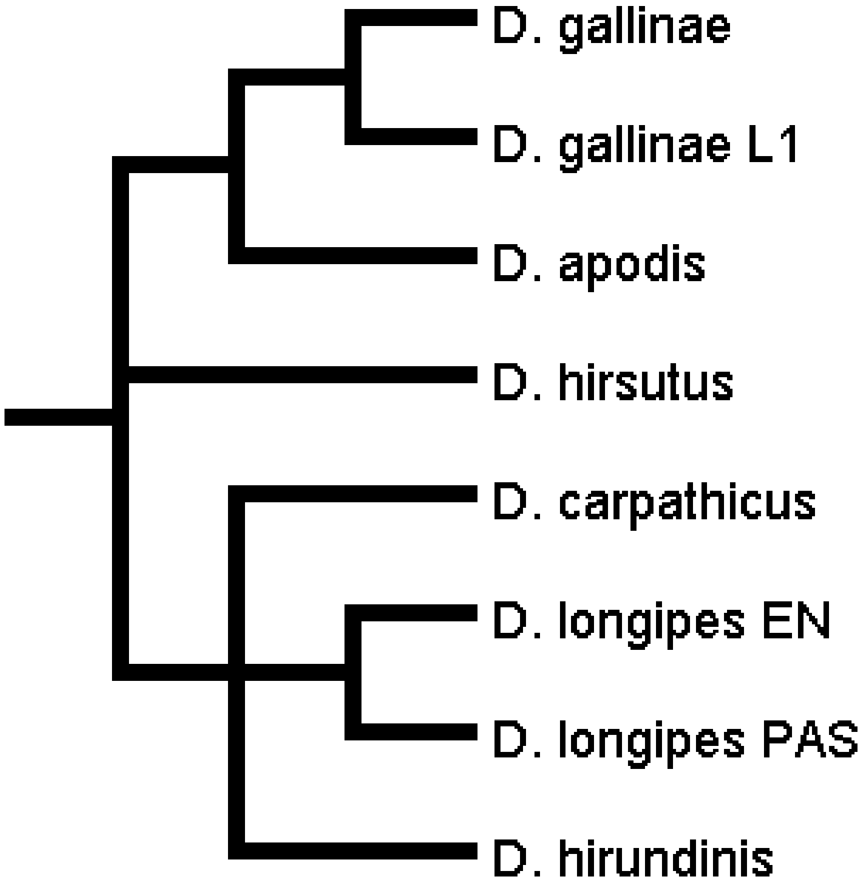
D. gallinae
D. gallinae L1
D. apodis
D. hirsutus
D. carpathicus
D. longipes EN
D. longipes PAS
D. hirundinis

Top29

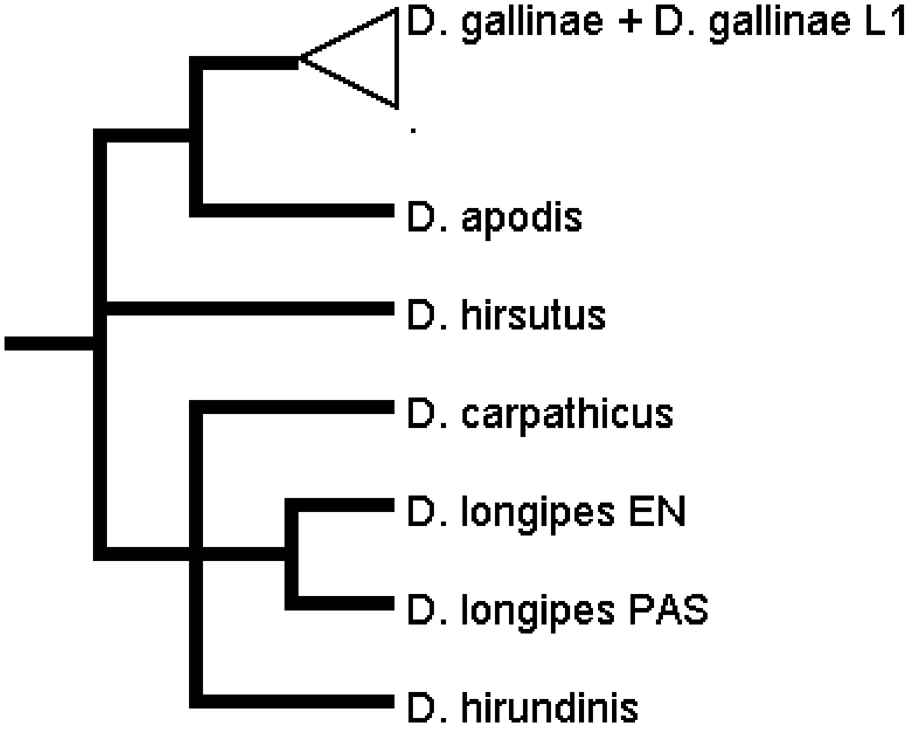
D. gallinae + D. gallinae L1
.
D. apodis
D. hirsutus
D. carpathicus
D. longipes EN
D. longipes PAS
D. hirundinis

Top31

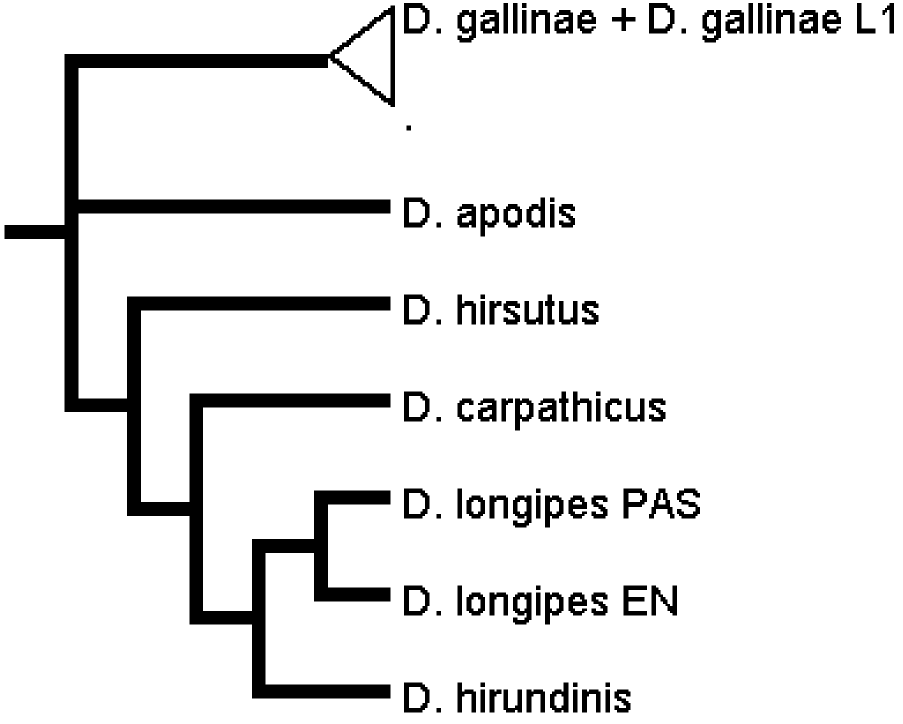
D. gallinae + D. gallinae L1
D. apodis
D. hirsutus
D. carpathicus
D. longipes PAS
D. longipes EN
D. hirundinis

Top32

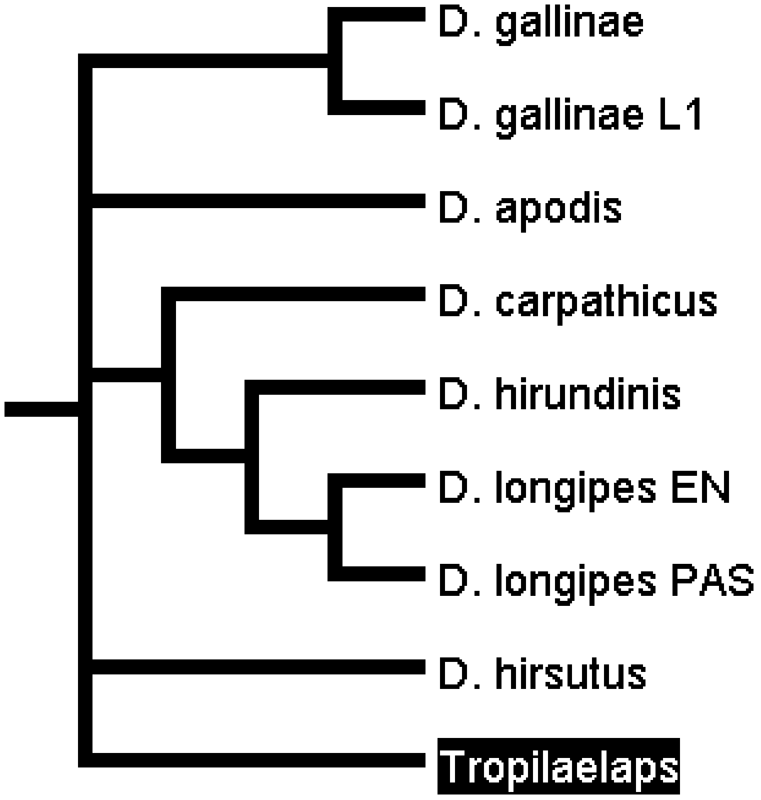
D. gallinae
D. gallinae L1
D. apodis
D. carpathicus
D. hirundinis
D. longipes EN
D. longipes PAS
D. hirsutus
Tropilaelaps

Top33

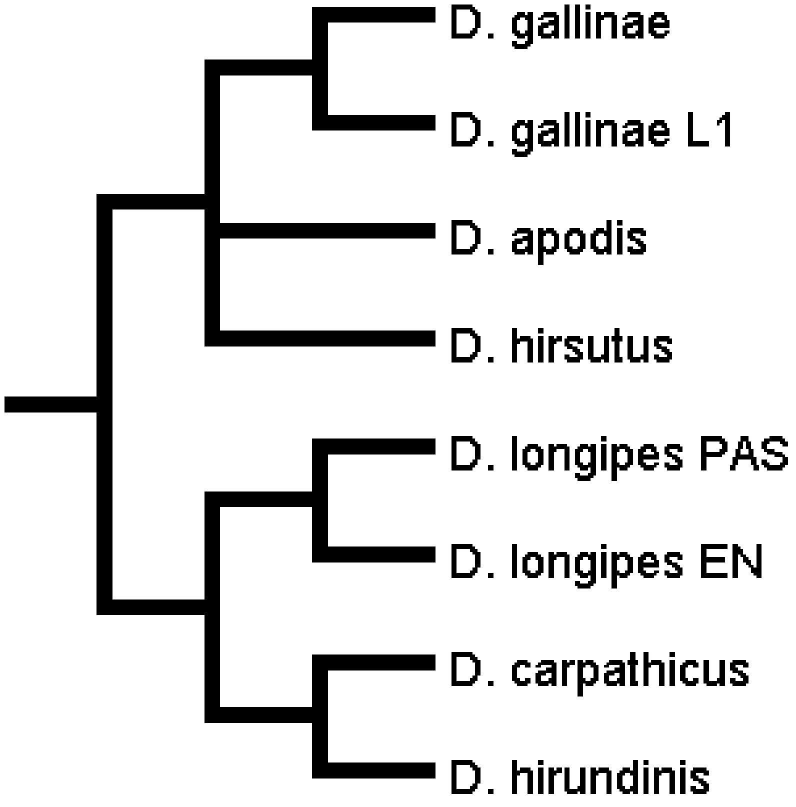
D. gallinae
D. gallinae L1
D. apodis
D. hirsutus
D. longipes PAS
D. longipes EN
D. carpathicus
D. hirundinis

Top35

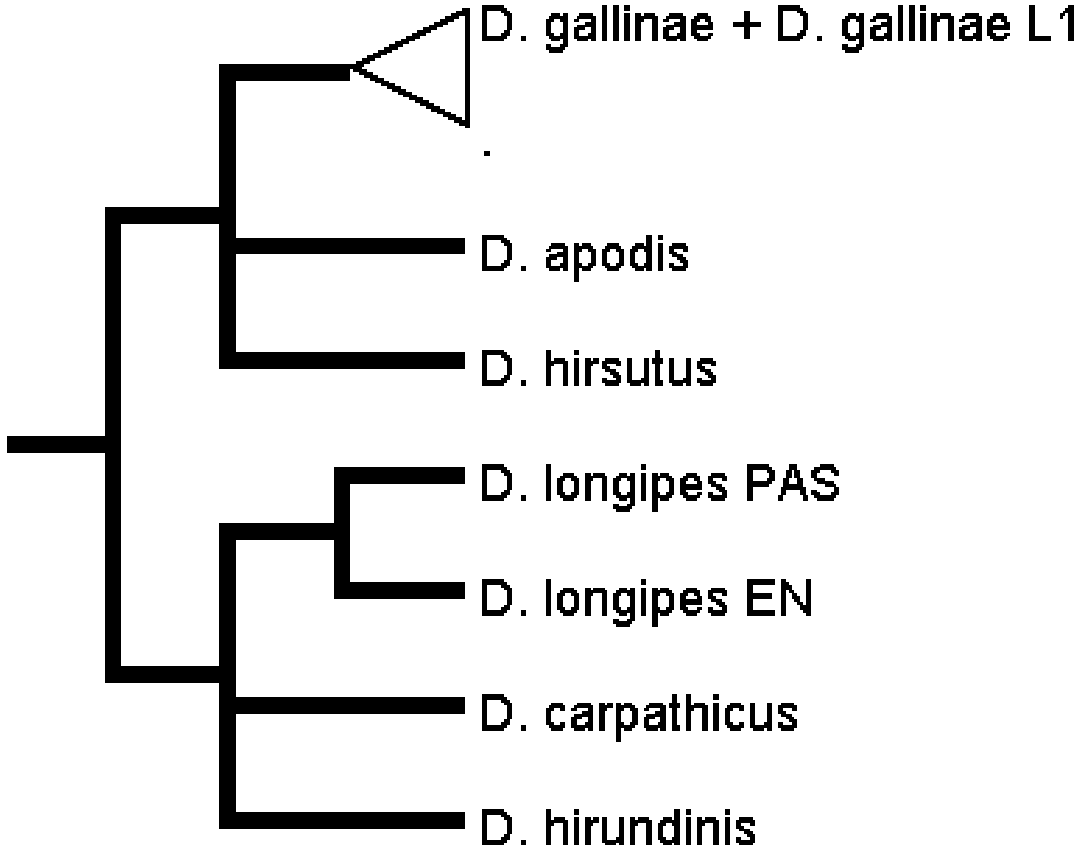
D. gallinae + D. gallinae L1
.
D. apodis
D. hirsutus
D. longipes PAS
D. longipes EN
D. carpathicus
D. hirundinis

Top36

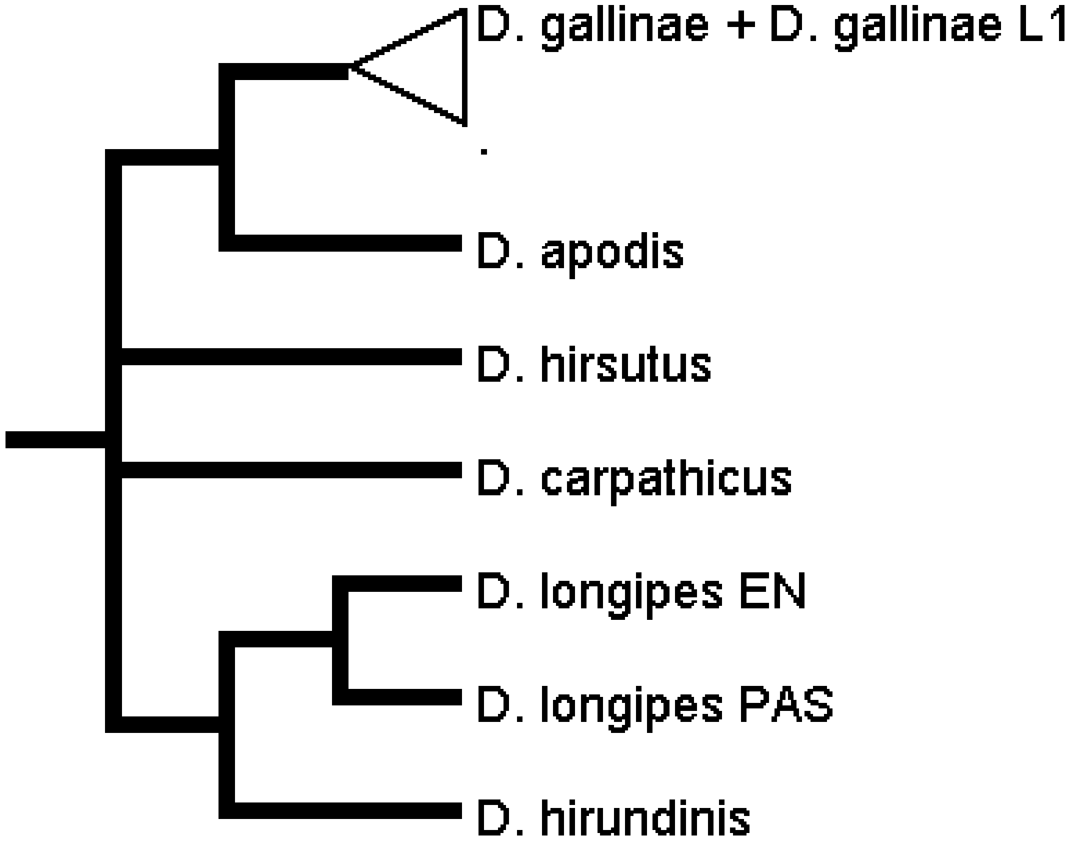
D. gallinae + D. gallinae L1
.
D. apodis
D. hirsutus
D. carpathicus
D. longipes EN
D. longipes PAS
D. hirundinis

Top37

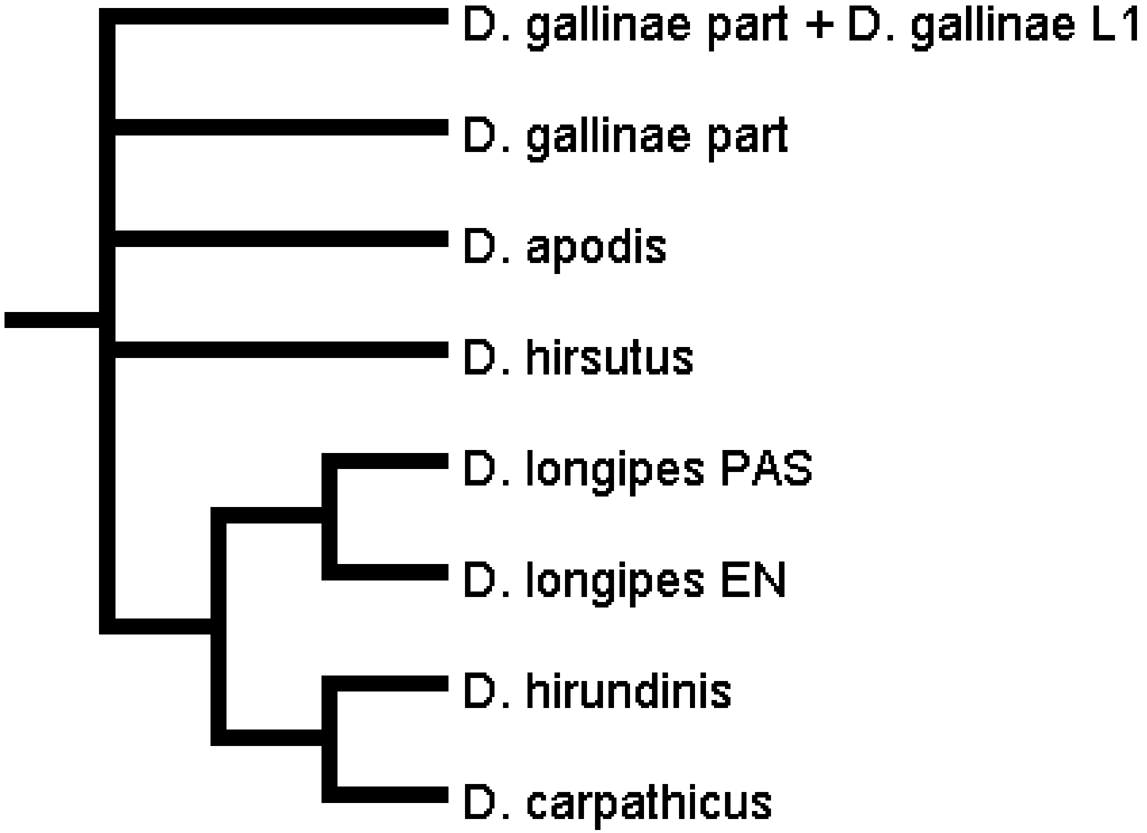
D. gallinae part + D. gallinae L1
D. gallinae part
D. apodis
D. hirsutus
D. longipes PAS
D. longipes EN
D. hirundinis
D. carpathicus

Top38

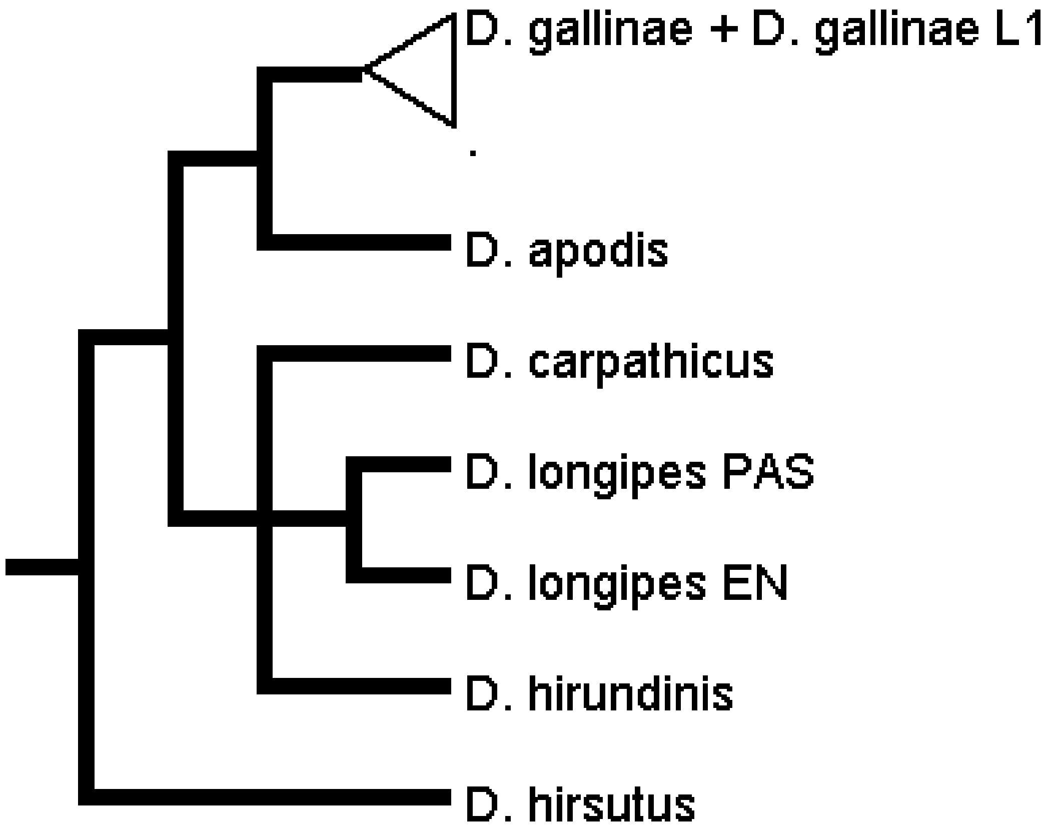
D. gallinae + D. gallinae L1
.
D. apodis
D. carpathicus
D. longipes PAS
D. longipes EN
D. hirundinis
D. hirsutus

Top39

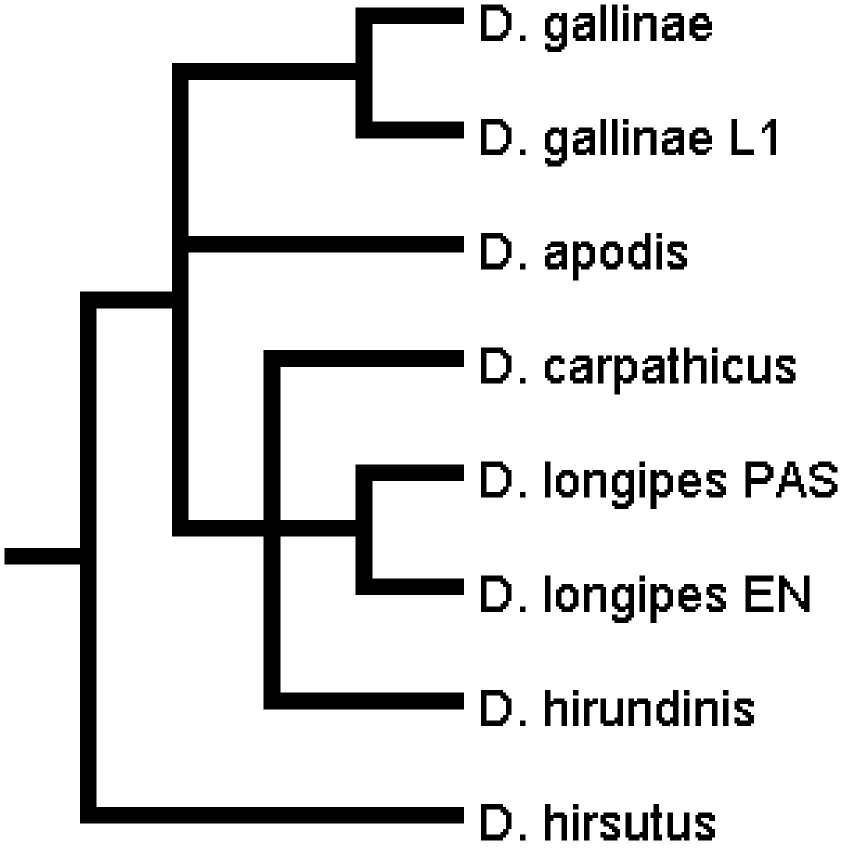
D. gallinae
D. gallinae L1
D. apodis
D. carpathicus
D. longipes PAS
D. longipes EN
D. hirundinis
D. hirsutus

Top40

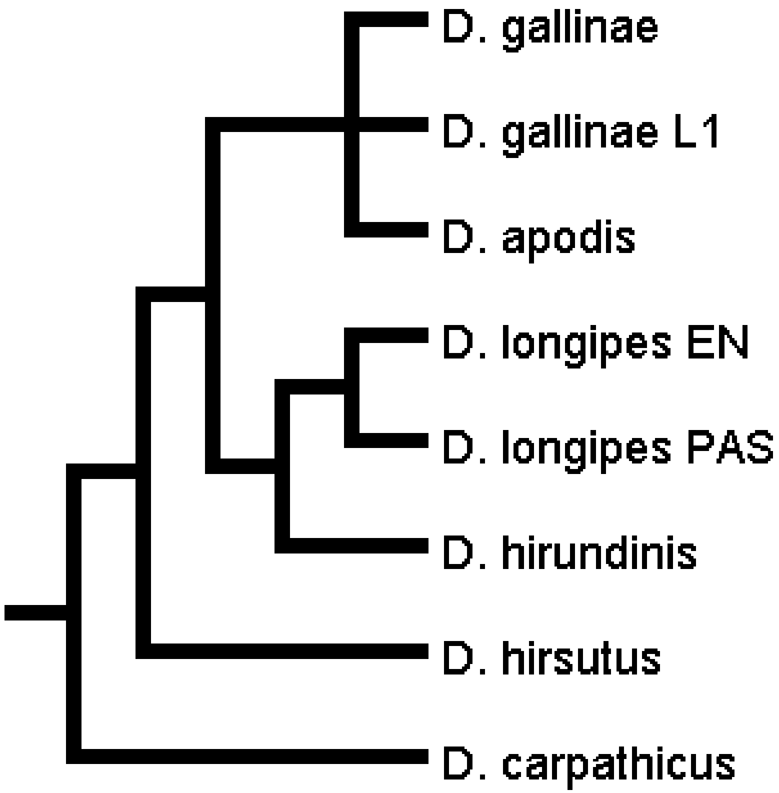
D. gallinae
D. gallinae L1
D. apodis
D. longipes EN
D. longipes PAS
D. hirundinis
D. hirsutus
D. carpathicus

Top41

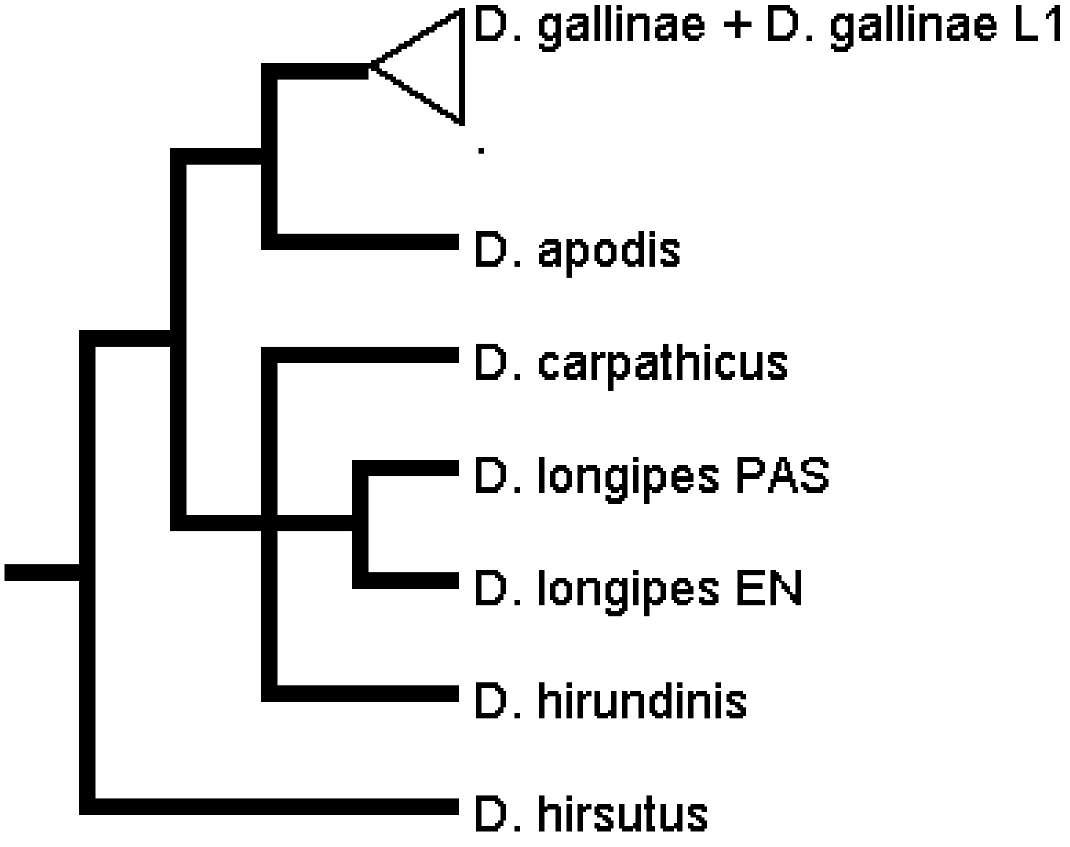
D. gallinae + D. gallinae L1
D. apodis
D. carpathicus
D. longipes PAS
D. longipes EN
D. hirundinis
D. hirsutus

Top42

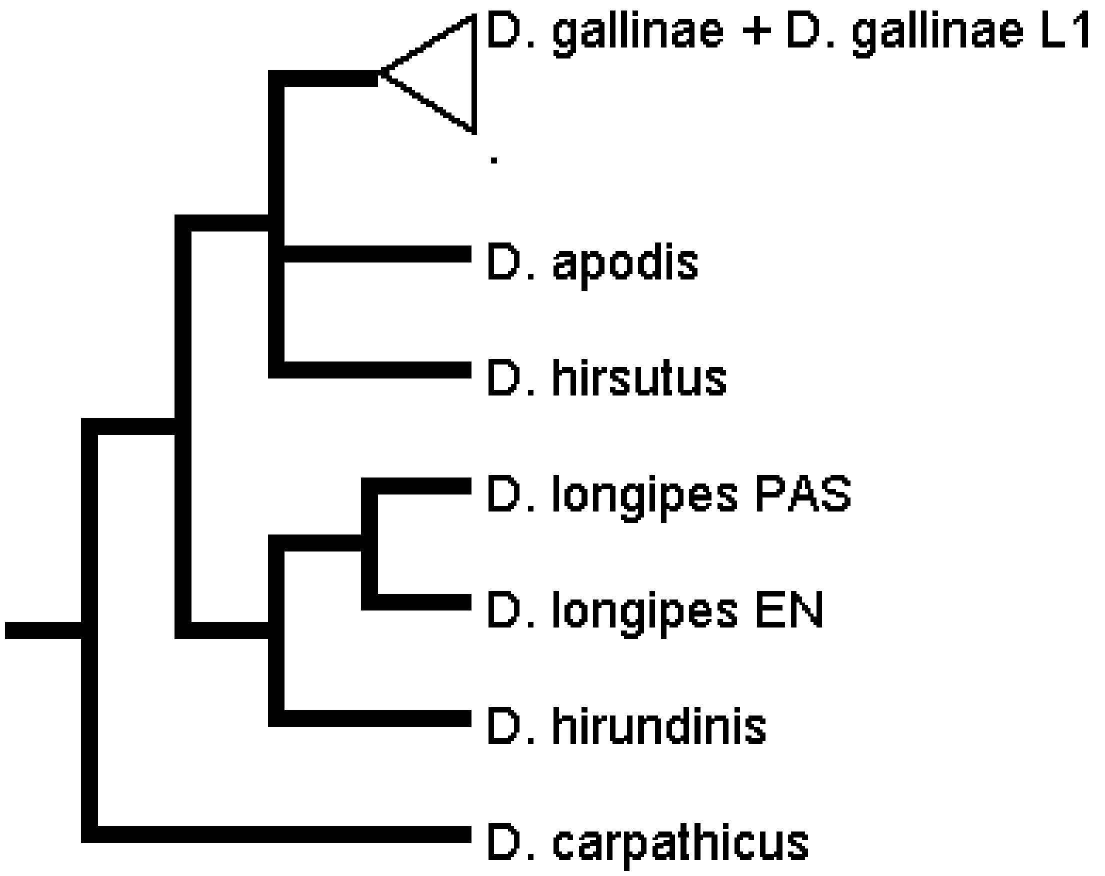
D. gallinae + D. gallinae L1
D. apodis
D. hirsutus
D. longipes PAS
D. longipes EN
D. hirundinis
D. carpathicus

Top43

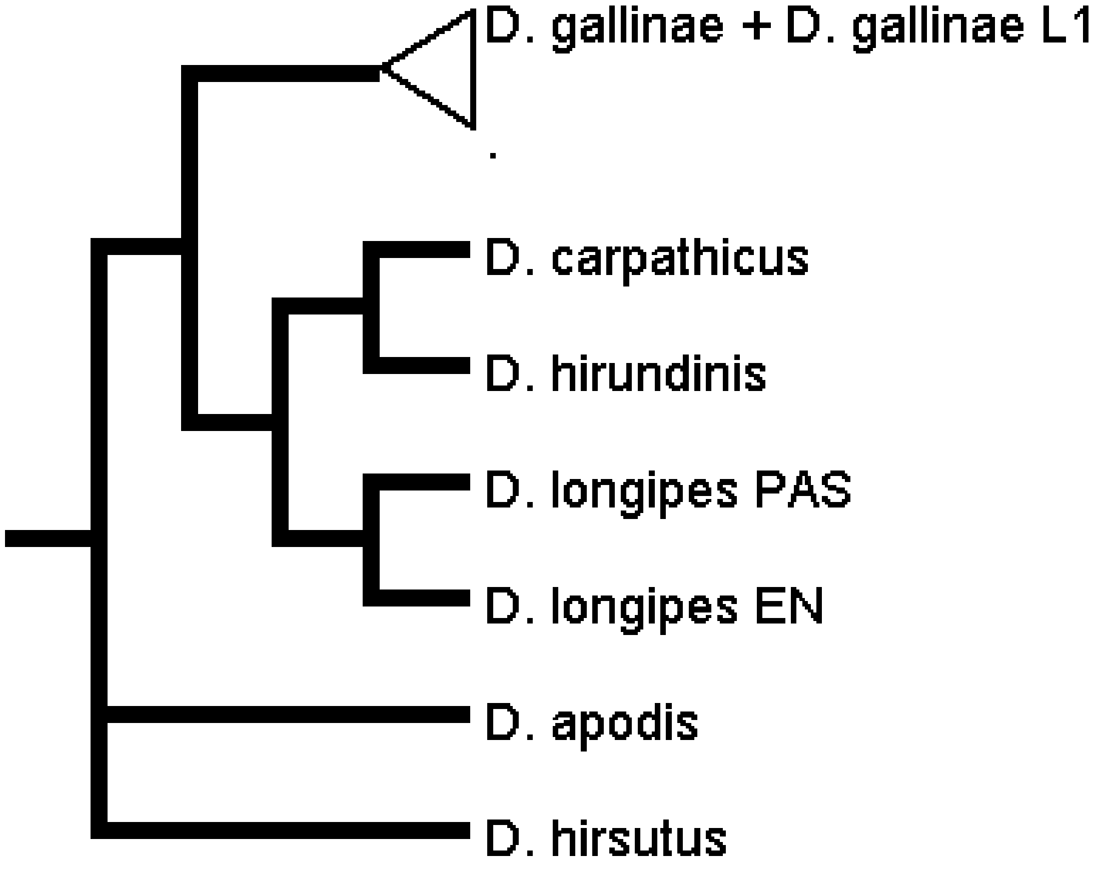
D. gallinae + D. gallinae L1
.
D. carpathicus
D. hirundinis
D. longipes PAS
D. longipes EN
D. apodis
D. hirsutus

Top44

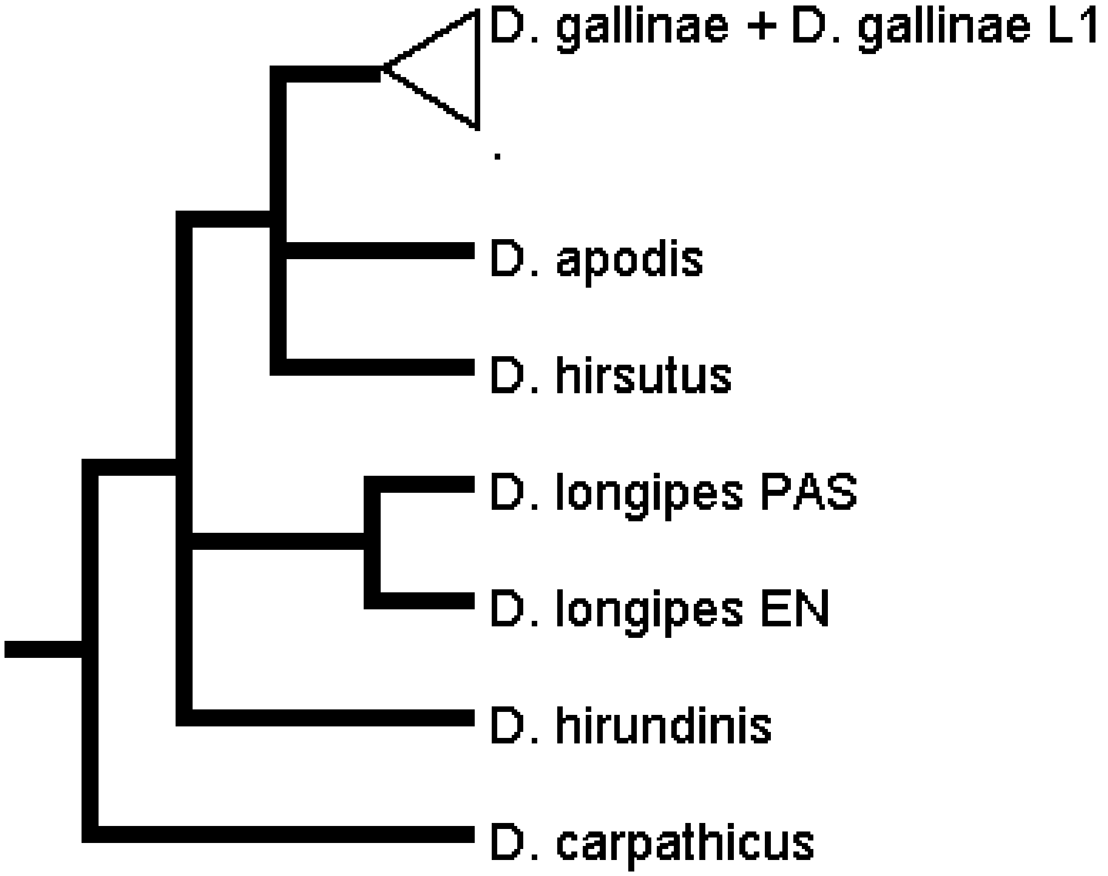
D. gallinae + D. gallinae L1
D. apodis
D. hirsutus
D. longipes PAS
D. longipes EN
D. hirundinis
D. carpathicus

Top45

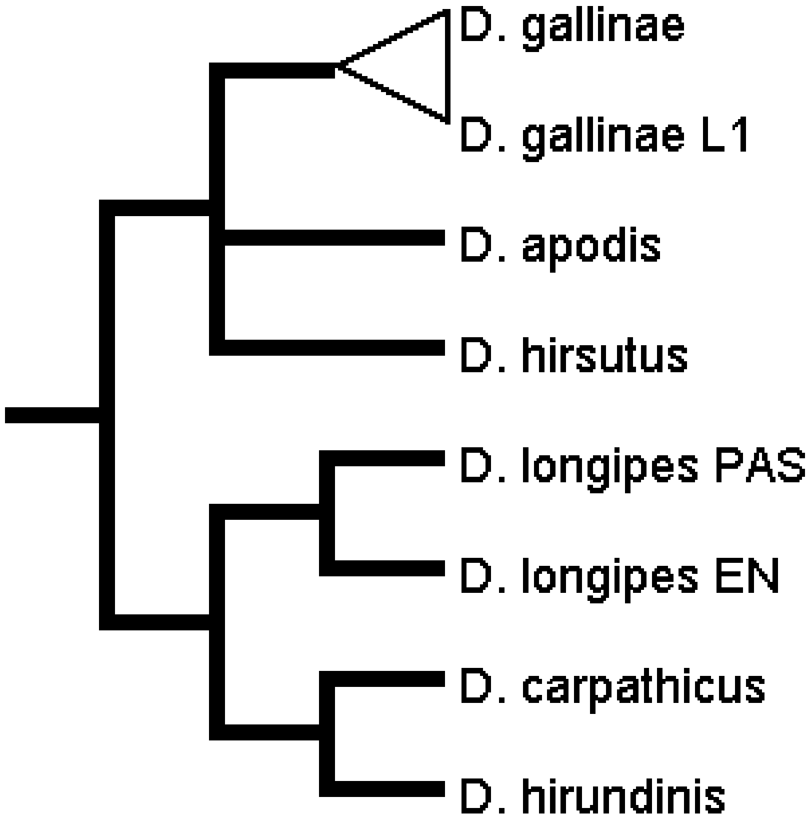

D. gallinae
D. gallinae L1
D. apodis
D. hirsutus
D. longipes PAS
D. longipes EN
D. carpathicus
D. hirundinis

Top46

Frequency of occurrence of topologies obtained in the different combinations tested in multi-gene analyses ordered according to the level of resolution. Solid bars represent the percentage of occurrence of topologies in MP analyses treating with gaps as the fifth state, open bars with gaps as missing data. Level of tree resolution was assessed considering *Dermanyssus* exclusively as follows: 0 = a single node (monophyly of *Dermanyssus*), 1 = specific entities delineated, 2 = 1 + one internal node, 3 = 2 + one internal nodes, and so on until 9 = specific entities delineated + all interrelationships resolved.

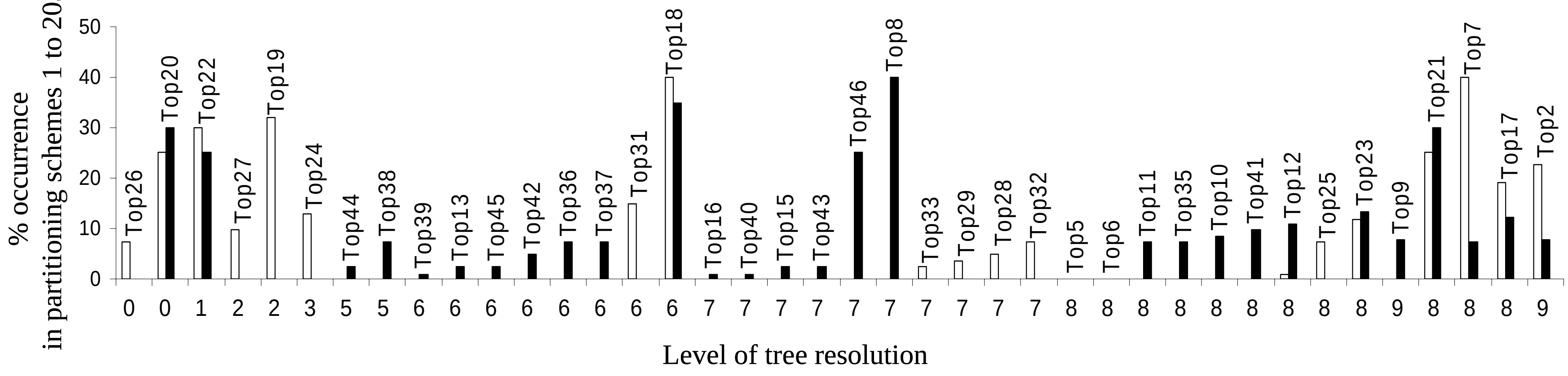

Supplement: Supplementary file 5 — Appendix 5 (The different topologies obtained at the specific level within Dermanyssus in multi-gene analyses and in multi-isolate analyses) [file app5.pdf]
