# Supplementary material for: Diversity of Phylogenetic Information According to the Locus and the Taxonomic Level: An Example from a Parasitic Mesostigmatid Mite Genus
Source: Int J Mol Sci. 2010 Apr 13;11(4):1704–34. doi: 10.3390/ijms11041704 (PMC2871134; doi:10.3390/ijms11041704)

Appendix 6. Haplotypic topologies obtained with COI and Tropomyosin sequences (for correspondence between haplotypes and isolates, see Appendix 1).

Topologies obtained using the Maximum parsimony criterion, PAUP4.0. Bootstrap support values listed above nodes before slash (/) and relative Bremex indices listed after slash. a, b and c. Tropomyosin based topologies. d. COI based topologies. (a) Gaps treated as a fifth state. Strict consensus of 264 most parsimonious trees (L=1288 CI=0.8238 RI=0.9320). (b) Gaps treated as missing data. Strict consensus of 1000 most parsimonious trees (L = 607; C I= 0.8023; RI = 0.9263). (c) Indels only, encoded following appendix. Strict consensus of 1000 most parsimonious trees (L = 80; CI = 0.7250; RI = 0.9450). (d) COI based topologies. Strict consensus of the 434 most parsimonious trees (L = 749; CI = 0.5340; RI = 0.8452).

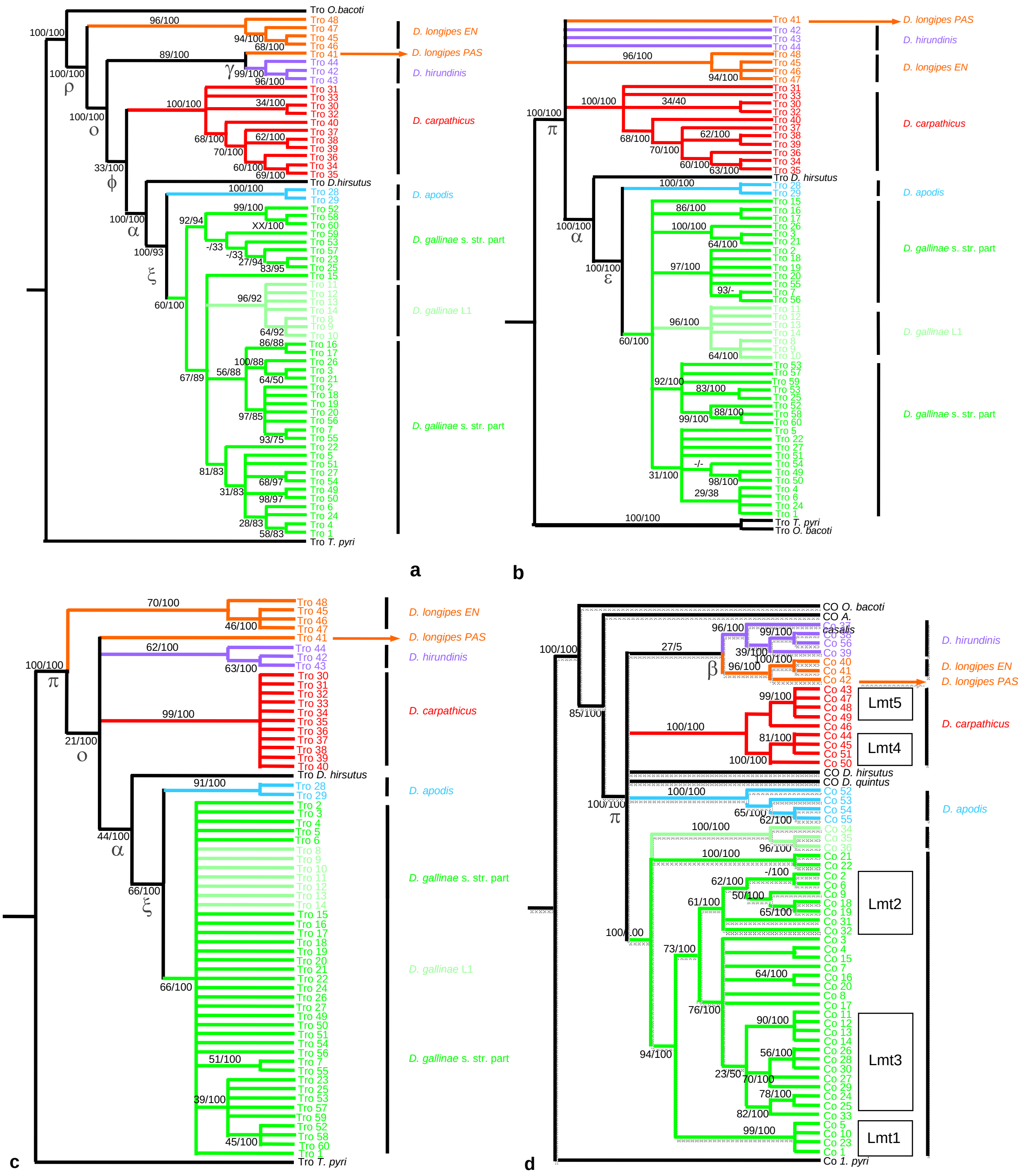

Supplement: Supplementary file 6 — Appendix 6 (MP topologies obtained in Step 2 not shown in Figure 4) [file app6.pdf]
